# Decoding Single Molecule Time Traces with Dynamic Disorder

**DOI:** 10.1371/journal.pcbi.1005286

**Published:** 2016-12-27

**Authors:** Wonseok Hwang, Il-Buem Lee, Seok-Cheol Hong, Changbong Hyeon

**Affiliations:** 1 Korea Institute for Advanced Study, Seoul, Republic of Korea; 2 Department of Physics, Korea University, Seoul, Republic of Korea; Max Planck Institute for Biophysical Chemistry, GERMANY

## Abstract

Single molecule time trajectories of biomolecules provide glimpses into complex folding landscapes that are difficult to visualize using conventional ensemble measurements. Recent experiments and theoretical analyses have highlighted dynamic disorder in certain classes of biomolecules, whose dynamic pattern of conformational transitions is affected by slower transition dynamics of internal state hidden in a low dimensional projection. A systematic means to analyze such data is, however, currently not well developed. Here we report a new algorithm—Variational Bayes-double chain Markov model (VB-DCMM)—to analyze single molecule time trajectories that display dynamic disorder. The proposed analysis employing VB-DCMM allows us to detect the presence of dynamic disorder, if any, in each trajectory, identify the number of internal states, and estimate transition rates between the internal states as well as the rates of conformational transition within each internal state. Applying VB-DCMM algorithm to single molecule FRET data of H-DNA in 100 mM-Na^+^ solution, followed by data clustering, we show that at least 6 kinetic paths linking 4 distinct internal states are required to correctly interpret the duplex-triplex transitions of H-DNA.

## Author Summary

We have developed a new algorithm to better decode single molecule data with dynamic disorder. Our new algorithm, which represents a substantial improvement over other methodologies, can detect the presence of dynamic disorder in each trajectory and quantify the kinetic characteristics of underlying energy landscape. As a model system, we applied our algorithm to the single molecule FRET time traces of H-DNA. While duplex-triplex transitions of H-DNA are conventionally interpreted in terms of two-state kinetics, slowly varying dynamic patterns corresponding to hidden internal states can also be identified from the individual time traces. Our algorithm reveals that at least 4 distinct internal states are required to correctly interpret the data.

## Introduction

Recent technological advances in single molecule experiments on biomolecules have provided an unprecedented chance to investigate dynamics of proteins and nucleic acids at single molecule (SM) level, which has previously been elusive in conventional experiments [1–7]. Folding/unfolding pathways gleaned from individual SM trajectories indicate rugged folding landscapes inherent to biomolecules [4, 8, 9]. Long time trajectories from SM measurements, which now can be extended more than hundreds seconds, allow us to address how a rugged conformational landscape is sampled over time [7, 10, 11]. One of the striking findings from these measurements is that even under the same folding condition, conformational dynamics of individual molecules differ substantially from one another while still maintaining their biological functions. Cofactor-induced conformational transitions of *T*. ribozymes [12], Holliday junctions [13], TPP-riboswitch [14], and preQ_1_-riboswitch [15] are the recent seminal examples that exhibit molecular heterogeneity at equilibrium. The variation in the velocities of individual RecBCD helicase motors along the dsDNA [16] is a good example of the molecular heterogeneity out of equilibrium, driven by ATP hydrolysis. Together with other reports [17–28], these could be merely a subset of more widespread, yet unrecognized cases that exhibit dynamical heterogeneity in SM time traces.

The chance of conformational frustration increases with the system size (*N*_sys_). For a given *N*_sys_, the time for conformational sampling (*τ*_*sample*_) is expected to scale as $\tau_{sample}∼e^{N_{sys}}$ [29]. Suppose that *T*_*obs*_, which is in practice limited by several factors [30–32], is long enough to observe many (more than hundreds) transitions along a trace generated from SM measurement. Two distinct scenarios arise depending on the length of *τ*_*sample*_ relative to *T*_*obs*_, (i) If the sampling time is shorter than *T*_*obs*_ (*τ*_*sample*_ ≪ *T*_*obs*_), then the conformational space of biomolecule is fully sampled. In this case, the ergodicity of the system is ensured such that for any molecule *α* (or time trace *α*) the time average of an observable *O*_*α*_, ${\langle O\rangle }_{T}=\frac{1}{T_{obs}}\int_{0}^{T_{obs}}O_{\alpha }\left(\tau \right)\;d\tau$, is equivalent to the ensemble average of *O*_*α*_(*t*) over all *α*’s (1 ≤ *α* ≤ *N*_*ens*_) at any moment *t*, $\frac{1}{N_{ens}}\sum_{\alpha =1}^{N_{ens}}O_{\alpha }\left(t\right)={\langle O\rangle }_{ens}$, i.e., 〈*O*〉_*T*_ = 〈*O*〉_*ens*_; thus thermodynamic properties of the system can be read out by analyzing a single time trace. (ii) In contrast, if *τ*_*sample*_ ≫ *T*_*obs*_ is satisfied due to ruggedness of conformational space characterised with a number of deep local basins of attraction, then each time trace can sample only a local region of the conformational space. In this case, dynamic pattern from each time trace would look different, and a change in the dynamic pattern from one time interval to another would be observed only occasionally.

**Fig 1:**
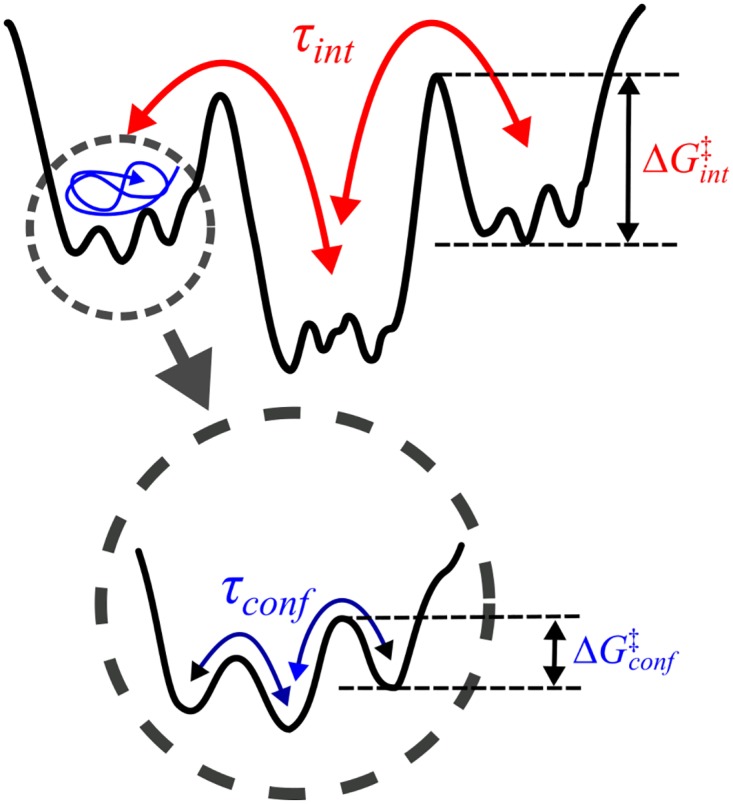
A rugged energy-landscape with hierarchical structure and an emergence of multiple time scales of transitions. *τ*_*int*_ is the transition time between different superbasins of attraction whereas *τ*_*conf*_ is the time scale of conformational dynamics of molecule *within* each basin. Due to large difference in kinetic barriers ($\Delta G_{int}^{‡}\gg \Delta G_{conf}^{‡}$), *τ*_*int*_ ≫ *τ*_*conf*_.

To be more precise about the second scenario (*τ*_*sample*_ ≫ *T*_*obs*_), suppose that the average time scale for each local basin of attraction to be “sampled” by the conformational dynamics of molecule is *τ*_*conf*_ and that the time for the molecule to make transitions between different superbasins of attraction is *τ*_*int*_ (Fig 1). In principle the relaxation rates and energy barrier heights of biomolecules span continuous spectra. So, the clear time scale separation may not always be waranteed. However, to be able to grasp the presence of dynamic disorder, if any, in SM time traces straightforwardly, a separation between two distinct time scales is required such that *τ*_*conf*_ ≪ *τ*_*int*_ (or $\Delta G_{conf}^{‡}\ll \Delta G_{int}^{‡}$). If *τ*_*conf*_ and *τ*_*int*_ were comparable (or the spectra of relaxation rates were uniform and continuous), an algorithm we will propose here as well as others could hardly be of any help to conceive a concrete landscape model as the one illustrated in Fig 1. Therefore, here we consider *τ*_*conf*_ and *τ*_*int*_ as two disparate time scales as illustrated in Fig 1. *τ*_*conf*_ is the time at which the time average of an observable ${\langle O\rangle }_{\tau }=\frac{1}{\tau }\int_{0}^{\tau }O\left(t\right)dt$ reaches its steady state value when *τ* > *τ*_*conf*_, corresponding to a time scale in which to fully sample the local basin of attraction. Alternatively, *τ*_*conf*_ is limited by a kinetic barrier with the greatest $\Delta G_{conf}^{‡}$ within the local basin of attraction, so that $\tau_{conf}\;≳\;e^{\Delta G_{conf}^{‡}/k_{B}T}$. On the other hand, *τ*_*int*_ is the transition time that is expected to scale with the height of kinetic barriers ($\Delta G_{int}^{‡}$) between the two superbasins as $\tau_{int}∼e^{\Delta G_{int}^{‡}/k_{B}T}$. When measurements are conducted with a finite duration of observation time (*T*_*obs*_), we can conceive two entirely different dynamic patterns depending on the relationship between *τ*_*conf*_, *τ*_*int*_, and *T*_*obs*_:

- *τ*_*conf*_ ≪ *T*_*obs*_ ≪ *τ*_*int*_: The interconversion time between distinct basins of attractions is far longer than the observation time. The dynamic patterns from individual trajectories that sample distinct basin of attraction are expected to differ from each other. Since *T*_*obs*_ ≪ *τ*_*int*_, there is few chance to observe an exchange of dynamic pattern in a single time trace, which corresponds to a case with quenched disorder that each SM time trace looks entirely different. Such cases are reported in Holliday junction [13], *T*. ribozyme [12], and RecBCD [16].
- *τ*_*conf*_ ≪ *τ*_*int*_ ≲*T*_*obs*_: The interconversion time between basins of attraction is shorter than or comparable to the observation time. In this case, it is possible to observe a few rounds (∼*T*_*obs*_/*τ*_*int*_) of pattern exchanges in a single time trace. Such SM time traces are called to have a dynamic disorder [15, 28, 33–36].

While the most interesting and physically relevant question to ask about the heterogeneity in single molecule time traces is its molecular origin, detection and quantification of such heterogeneity should precede such question for a further analysis. For SM time traces with quenched disorder, it is relatively straightforward to analyze as one can use the criterion of ergodicity and partition each time trace into its dynamic subensembles [13]. It is, however, more challenging to analyze time traces with dynamic disorder.

In the ion-channel community, ion currents across a single ion-channel measured with patch-clamp technique often demonstrate time series that switch between multiple dynamic patterns, and such a phenomenon is called ‘mode-switching’ [37] or ‘modal gating’ [38]. An algorithm (aggregated Markov model, AMM) developed by ion-channel community to analyze time series exhibiting dynamics disorder is in principle of use, but when applied to our synthetic data, we found that the algorithm tends to overpredict the transitions between hidden states (see S25 Fig and discussion related to it below). Thus, here we have developed a more reliable and systematic algorithm—Variational Bayes-Double Chain Markov Model (VB-DCMM)—which combined variational Bayes method with Double Chain Markov Model (DCMM) [39–43], to analyze SM time traces with dynamic disorder in which dynamic pattern of conformational transition changes at much longer time scale than apparent conformational fluctuations due to a slower transition of a *hidden* variable.

We first explain the algorithm for VB-DCMM, and next apply our VB-DCMM method to synthetic data as a blind test to show that our method can accurately identify the hidden internal states and determine the kinetic rate constants associated with the data. The results from our analysis using VB-DCMM are reliable as long as a clear separation in time scales exists between the apparent conformational transition (*τ*_*conf*_) and the interconversion times (*τ*_*int*_).

**Fig 2:**
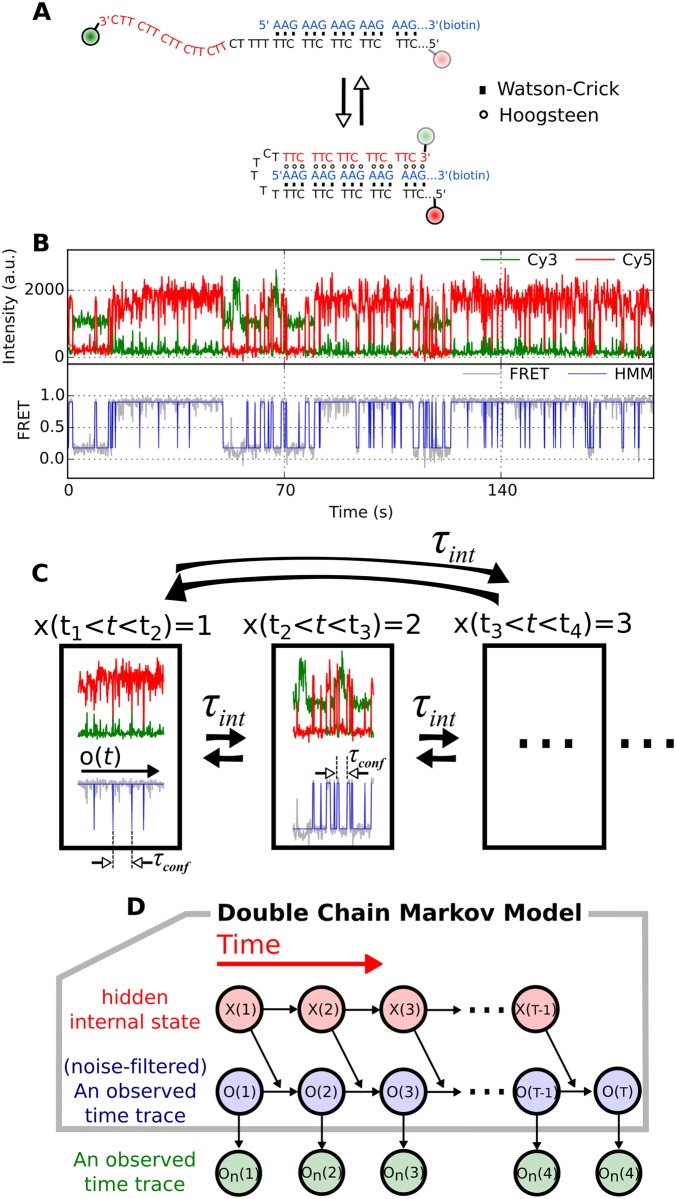
Duplex-triplex transitions of H-DNA with dynamic disorder. (A) Illustration of H-DNA dynamics. The sequences in blue and black form duplex via Watson-Crick base pairing; the sequences in red extended from 3’-end region of the black sequence can pair with the sequences in blue via Hoogsteen base pairs to form the triplex helix. (B) A time trace of H-DNA displaying dynamic disorder. (Top) The fluorescence signals from Cy3 (green) and Cy5 (red) dyes. (Bottom) FRET signal (gray) was calculated using the signals from Cy3 and Cy5. Blue line is the noise-filtered FRET signal obtained using HMM. The low-FRET (~0.1) and high-FRET state (~0.9) correspond to the duplex and triplex states, respectively. The dynamic pattern of the time trace changes occasionally from one time interval to another. For example, the transitions from low to high FRET state around 70 s are much slower compared with those around 140 s. (C) The model for H-DNA dynamics with dynamic disorder. Hierarchical transitions, (1) transitions within *x*(*t*) = *i*, and (2) interconversion between *x*(*t*) = *i* and *x*(*t*′) = *j* (*i* ≠ *j*), can be described using Double Chain Markov Model (DCMM). (D) Graphical representation of DCMM. *x*(*t*), *o*(*t*), and *o_n_*(*t*) represent internal state, noise-filtered observable (blue line in (B)), and the original observable at time t (gray line in (B)), respectively. The black arrows signify how each state is determined by others. For example, the state of observable at time *t*, *o*(*t*) is determined by the previous observable state at time *t* − 1, o(*t* − 1), and the state of the previous internal state, *x*(*t* − 1).

As a prototypical example of single molecule time traces with dynamic disorder, data from H-DNA [44, 45] that undergoes duplex-to-triplex conformational transitions (Fig 2A) are analyzed. A kinetic pattern of two-state like conformational transitions between duplex (low FRET ∼ 0.1) and triplex form of H-DNA (high FRET ∼ 0.9) observed in one time interval changes to another pattern in the next time interval (Fig 2B). DCMM models this peculiar dynamic pattern of H-DNA in Fig 2B by assuming a slowly varying dynamics of a hidden internal state. Fig 2C illustrates how the dynamic pattern of the original time trace of *observable state*, *o*_*n*_(*t*) (gray traces in Fig 2C), changes with the *internal state* *x*(*t*) at a given time *t*. The dynamic pattern of *o*_*n*_(*t*), displaying multiple transitions, is slave to the slowly changing value of *x*(*t*). DCMM implements this idea into an algorithm and allows us to extract the information of *x*(*t*) from *o*_*n*_(*t*). Finally, we apply VB-DCMM to an ensemble of H-DNA time traces obtained from smFRET experiments and show that the dynamics of H-DNA at [Na^+^] = 100 mM should be modeled using at least 4 large basins of attraction.

## Algorithm

Here, we provide a general overview of the VB-DCMM algorithm, defining terms and parameters. More technical details of derivation and implementation of the algorithm are given in the Supplementary Information.

### Modeling time series with dynamic disorder

Markov chain approach is ubiquitously used in modeling biological systems. For example, reversible conformational transitions of biomolecules probed by single molecule fluorescence resonance energy transfer (smFRET) or force spectroscopy are often modeled as a homogeneous Markov process in which the transition rates between experimentally discernible conformational states are uniquely decided. To decipher time series with dynamic disorder that change their dynamic pattern from one time interval to another we assume that there are hidden “internal states”, each of which determines the rate of conformational transitions. A signature of the transition between internal states, which gives rise to dynamic disorder in time series, are difficult to detect using the value of FRET efficiency or end-to-end distance alone when the values observed along the time series are indiscernible even if the internal state is altered. By assuming that the transition between internal states is described by a homogeneous Markov process, and that transition between observable (in this study, FRET) follows non-homogeneous Markov process, whose transition rates are slaved to the internal state at each time, we model time trajectories made of these two layers of Markov chains. This algorithm corresponds to the Double Chain Markov Model (DCMM) [39–43] (Fig 2C and 2D).

DCMM is characterized by the following model parameters: (i) Transition matrix ***A*** for homogeneous Markov chain, which describes the transition probability between the *K*-distinct internal states along the time series (***x*** = (*x*(1), *x*(2), …, *x*(*t*), ⋯, *x*(*T* − 1))). Here *K* is a total number of internal states in the model, and *x*(*t*), specifying internal state at time *t*, takes one of the values between 1 and *K*. *T* is the total observation time. The internal state at time *t*+1 (*x*(*t* + 1)) is determined by the previous internal state at time *t* (*x*(*t*)), whose transition to *x*(*t* + 1) is determined by a *K* × *K* Markov transition matrix ***A*** as $P\left(x\left(t+1\right)=\mu \right)=\sum_{\nu =1}^{K}A_{\mu ,\nu }P\left(x\left(t\right)=\nu \right)$ where *P*(*x*(*t*) = *ν*) denotes the probability of *x*(*t*) being in the *ν*-th internal state; (ii) *K* transition matrices ***B***^(*μ*)^ with *μ* ∈ {1, 2, …, *K*} for non-homogeneous Markov chain describes the transition probability between the observable states along the time series (***o*** = (*o*(1), *o*(2), …, *o*(*T*))). *o*(*t*) specifies the state of the observable among *N* possible states {1, 2, …, *N*} at time *t*. Transition from *o*(*t*) to *o*(*t* + 1) is determined by an *N* × *N* transition matrix ***B***^*x*(*t*)^(*t*), the matrix elements of which are slave to the value of *x*(*t*)(= *μ* ∈ {1, 2, …, *K*}).

For example, if there are two (*K* = 2) internal states, and each internal state has three (*N* = 3) observables in a given time trace recorded with time resolution Δ*t*, then two transition matrices for ***o*** with *μ* = 1, 2 can be considered (i.e., ***B***^(1)^ and ***B***^(2)^):

$$B^{\left(\mu \right)}=\left(\begin{array}{ccc} k_{1\to 1}^{\left(\mu \right)}\Delta t & k_{1\to 2}^{\left(\mu \right)}\Delta t & k_{1\to 3}^{\left(\mu \right)}\Delta t\\ k_{2\to 1}^{\left(\mu \right)}\Delta t & k_{2\to 2}^{\left(\mu \right)}\Delta t & k_{2\to 3}^{\left(\mu \right)}\Delta t\\ k_{3\to 1}^{\left(\mu \right)}\Delta t & k_{3\to 2}^{\left(\mu \right)}\Delta t & k_{3\to 3}^{\left(\mu \right)}\Delta t \end{array}\right).$$

Next, the transition matrix ***A*** for the interconversion between two internal states is:

$$A=\left(\begin{array}{cc} \gamma^{\left(1)\to (1\right)}\Delta t & \gamma^{\left(1)\to (2\right)}\Delta t\\ \gamma^{\left(2)\to (1\right)}\Delta t & \gamma^{\left(2)\to (2\right)}\Delta t \end{array}\right).$$

In the above matrices, the matrix elements must satisfy, $\sum_{j=1}^{3}k_{i\to j}^{\left(\mu \right)}\Delta t=1$ for each *i* = 1, 2, 3 in ***B***^(*μ*)^, and *γ*^(1)→(1)^Δ*t* + *γ*^(1)→(2)^Δ*t* = *γ*^(2)→(1)^Δ*t* + *γ*^(2)→(2)^Δ*t* = 1 in ***A***. More detailed descriptions about DCMM are available in the original papers [39–43] particularly in ref. [39] (see also **SI**). A similar but more general version of DCMM, which can accommodate inputs variables as well as multiple number of internal state sequences, has been suggested by extending the factorial hidden Markov model [46, 47].

### Determining the number of internal states

DCMM can estimate the transition matrices ***A*** and ***B***^(*μ*)^ quantitatively, and hence determine the most probable sequence of internal state and associated kinetic rates, $\left\{k_{a\to b}^{\left(\mu \right)}\right\}$ and {*γ*^(*μ*)→(*ν*)^}. However, the likelihood (the probability of observing data for given model parameters), maximized by DCMM, *P*(***o***|***π***, ***A***, ***B***) where ***π*** ≡ (*π*_1_, *π*_2_, …, *π*_*K*_) with *π*_*μ*_ = *P*(*x*(1) = *μ*|***o***, ***A***, ***B***), is prone to increase when more number of parameters are used in the model. DCMM can select the best set of parameters for a given model, but not suited to select the best model (i.e., cannot determine the optimal number of internal states *K* for a given time trace). To overcome this limitation, often used is the maximum evidence method, where the *evidence* (*P*(***o***|*K*), also called marginal likelihood) is defined as the conditional probability of observing data (***o***) for a given model (*K*), so that

P(o|K)=∫P(o|λ)P(λ|K)dλ (1)

where ***λ*** ≡ (***π***, ***A***, ***B***) represents the parameter space. In this method, the penalty against model complexity is naturally incorporated during the calculation, allowing to select the best model (see **SI**). By calculating the evidence for each different model (different *K*, the number of internal states in data), one can select the best model with an optimal number of internal states that maximizes the evidence. The calculation of the evidence, however, involves a massive computational cost to explore the entire parameter space for a given model.

### Variational Bayes double chain Markov model

To alleviate the computational cost in employing the maximum evidence method in Eq 1, we employ the Variational Bayes [48], a method that effectively uses a mean-field approximation. The method has previously been used to determine the number of observable states (FRET states) from smFRET data [49–51], the number of diffusive states from single molecule tracking data [52], and the number of DNA-protein conformations from tethered particle motion data [53]. It has also been used inside the empirical Bayes method which can analyze several smFRET time series simultaneously [54, 55]. In our study, the variational Bayes method combined with DCMM (VB-DCMM) was used to analyze single molecule time traces with dynamic disorder. The analytical expression of the lower bound of the *evidence* (*F*), offered by VB-DCMM, makes clear where the model penalty comes from, thus providing guidelines to choose the prior parameters to incorporate a prior knowledge of data (see **SI**). Once prior parameters are selected, VB-DCMM iteratively increases the lower bound of log(*evidence*)(= log *P*(***o***|*K*)) by identifying a better approximation to the true probability distribution.

$$\begin{array}{ll} \text{log }P(o|K) & =\int q\left(Z\right)\text{log }P(o|K)dZ\\ & =F\left[q\right]+D_{KL}(q||p)\geq F\left[q^{*}\right]. \end{array}$$ (2)

where *q*(***Z***) is an arbitrary probability distribution of a set of variables, ***Z***(≡ (***x***, ***λ***)) consisting of parameters and hidden variables of model,

$$F\left[q\right]\equiv \int q\left(Z\right)\log\left(P(o,Z|K)/q(Z)\right)$$

and

$$D_{KL}\left(q||p\right)\equiv \int q\left(Z\right)\log\left(q(Z)/P(Z|o,K)\right)\geq 0,$$

where *D*_*KL*_(*q*||*p*) is the Kullback-Leibler divergence of *q*(***Z***) from *P*(***Z***|***o***, *K*), which we want to minimize. Once the solution from the algorithm converges, the approximate value of log *P*(***o***|K*)(≃ *F*[q*]) and the (locally) best model parameters (a set of the best kinetic rates), π*, A* and B*, which determines all the rate constants to describe the given time traces ($\left\{k_{a\to b}^{\left(\mu \right)}\right\}$ and {*γ*^(*μ*)→(*ν*)^}), are acquired from an approximated probability distribution (See SI for the mathematical details). The performance of VB-DCMM is quite robust over a wide variation of prior parameters (S21 and S22 Figs).

### Implementation of the algorithm

The observable sequence ***o*** is obtained by filtering the noise in the experimental data (***o***_*n*_) using Hidden Markov Model (HMM) following a similar procedure as the previous studies [49, 56] using a custom code written based on the code from Sagemath software [57]. Next, the ***o*** is analyzed using VB-DCMM to select the best model and to estimate the best model parameters. The optimal sequence of internal states ***x*** is determined by using Viterbi algorithm [39]. All the implementations and data analysis are done by using our custom code. VB-DCMM is freely available at “https://github.com/TBiophysG/VBDCMM”

## Results and Discussion

### Validation of VB-DCMM

**Fig 3:**
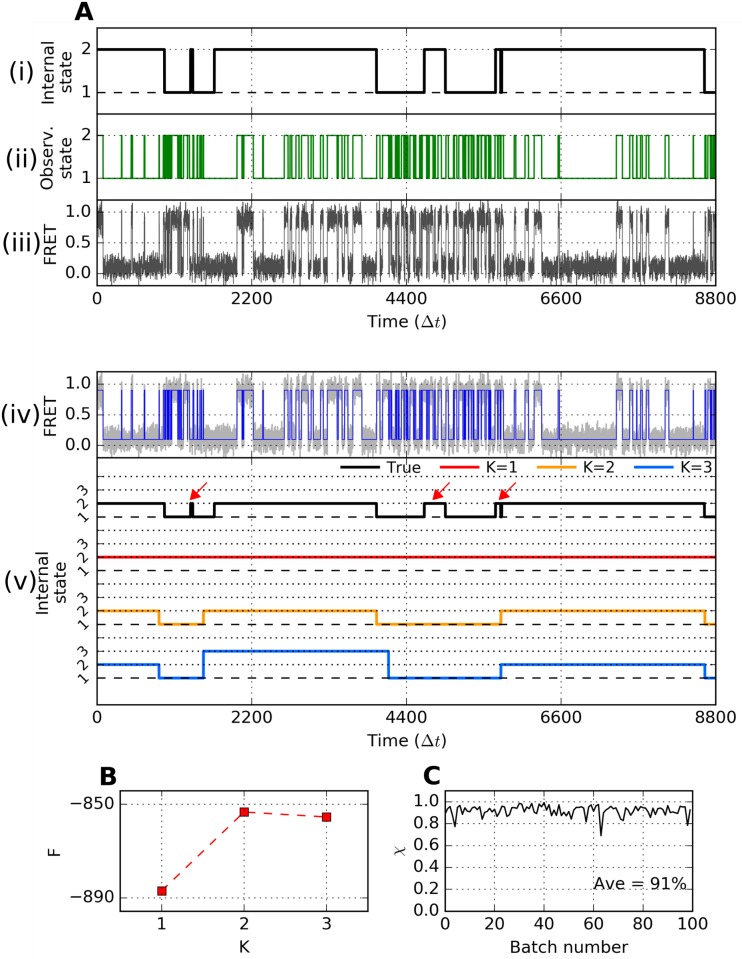
Validation of VB-DCMM on synthetic data. (A) (i) A time trace of internal state generated with *γ*^(1)→(2)^Δ*t* = γ^(2)→(1)^Δ*t* = 0.001. (ii) An observable time trace generated based on the trace of internal state in (i) by using internal state-dependent parameters $k_{L\to H}^{\left(1\right)}\Delta t=k_{H\to L}^{\left(1\right)}\Delta t=0.05$, $k_{L\to H}^{\left(2\right)}\Delta t=0.00625$, $k_{L\to H}^{\left(2\right)}\Delta t=0.025$. (iii) An synthetic FRET data with Gaussian noise overlaid on the trace in (ii). (iv) Noised filtered FRET state by HMM (blue line). (v) Traces of internal state with different *K*, estimated using VB-DCMM on the noise-filtered FRET trace from (iv) (black line is the true internal state trace while red, orange, and blue are internal state estimated from the model with *K* = 1, 2, and 3, respectively. The indices of internal state were determined by comparing ***B***^(*μ*)^ estimated for each internal state with ***B***^(*μ*),true^ which is used to generate the synthetic data). (B) Estimated lower bound of the evidence function *F*(*K*) of DCMM models with *K* = 1, 2, and 3. (C) Accuracy of detecting internal states. The overlap function *χ* calculated for 100 synthetic FRET traces generated under the identical condition used for generating the trace of internal state shown in (A).

To first validate the efficacy of VB-DCMM in identifying internal states in a given SM time trace, we applied VB-DCMM algorithm on synthetic data that mimic a SM time trajectory with dynamic disorder (see Methods). To generate a synthetic SM time trajectory, we first produce a time trajectory specifying the value of internal state from *t* = 1 to *t* = *T* − 1. The time trajectory of internal state is represented with a symbol ***x*** ≡ (*x*(1), *x*(2), ⋯, *x*(*t*), ⋯, *x*(*T* − 1)). When the total number of distinct internal states in the model is *K*, one of the values in {1, 2, ⋯, *K*} is assigned to *x*(*t*). Thus, for *K* = 2 a typical time trajectory of internal state ***x*** looks like (1, 1, 1, ⋯, 1, 1, 2, 2, 2, ⋯, 2, 2, 1, 1, ⋯, 1, 1), (1, 1, 1, ⋯, 1, 2, 2, ⋯, 2, 2), (2, 2, ⋯, 2, 1, 1, ⋯, 1, 1, 2, 2, ⋯, 2, 2, 2), etc. The time trajectory given in Fig 3A(i) is an example generated with *K* = 2 and *T* = 8801. Next, similar to the structure of ***x***, the time trajectory of noiseless observables is represented using ***o*** ≡ (*o*(1), *o*(2), ⋯, *o*(*t*), ⋯, *o*(*T*)). In Fig 3A(ii), a trajectory of ***o*** is shown, also demonstrating the influence of ***x*** on ***o***. Finally, Gaussian noise was added on ***o*** and the range of signal was adjusted to produce the final trajectory ***o***_*n*_ ≡ (*o*_*n*_(1), *o*_*n*_(2), ⋯, *o*_*n*_(*T*)) which now resembles a time trajectory of SM FRET signal (Fig 3A(iii)).

Deciphering the information of internal states from an observed time trace involves solving an inverse problem, i.e., decoding ***o***_*n*_ to obtain ***x***. To decode the trace of internal states from the synthetic data, we follow a 3-step procedure: (1) Filter the noise from ***o***_*n*_ to obtain ***o*** using Hidden Markov Model (HMM) [56] (Fig 3A(iv), blue line); (2) Analyze ***o*** by applying VB-DCMM algorithm with different models 1, 2, …, *K* (again, *K* is the total number of internal states assumed in each model); (3) To select the best model we calculated the conditional probability of observing data for a given model parameter *K*, *P*(***o***|*K*), which is often called *evidence* or *marginal likelihood* in machine learning community (Eq 2) [48]. Calculation of *P*(***o***|*K*) is conducted using the Variational Bayes (VB) method, which gives the lower bound of log *P*(***o***|*K*) denoted by *F*(*K*). Details of the evidence function *F*(*K*) and approximation procedure are provided in the Supplementary Information (SI). Finally, we select the best model K* which maximizes *F*(*K*), i.e., K* = arg max *F*(*K*).

To be specific, in order to identify the best model parameter *K* for the time trace ***o***(*t*) given in Fig 3A(iv), we varied *K* from 1 to 3. The most probable trace of internal states, $x_{\left(K\right)}^{\text{model}}$, was calculated for each model with *K* = 1 (red), *K* = 2 (orange), *K* = 3 (blue) (see Fig 3A(v)). The evidence *F*(*K*) calculated using VB method was maximized at *K* = K* = 2, and the resulting time trace of the internal states, $x_{\left(K^{*}=2\right)}^{\text{model}}$, most closely recovers the trajectory of ***x*** (black trace in Fig 3A(v)) except at the time interval where the transitions of *x*(*t*) between 1 and 2 occur only transiently or at the boundaries of transitions (red arrows on Fig 3A(v)). This result shows that VB-DCMM can avoid the over-fitting problem that other methods based on maximum likelihood are often fraught with [48].

### Conditions required for an accurate recovery of internal states

VB-DCMM detects a signature of change in internal state (*x*) from a given observable time trace (***o***) by evaluating the statistical difference in transition rates. Thus, in the absence of an enough number of transitions in the trace ***o***, the algorithm becomes less reliable. For example, we obtained *F*(*K* = 2) ≈ *F*(*K* = 3) although *F*(2) ≫ *F*(3) is more desirable (Fig 3B. See another example in S1 Fig). This is due to the lack of statistics in transition events in this particular test trace given in Fig 3A. For example, when only a part of the time trace is selected and analyzed using HMM, the estimated rates of transition from high (*H*) to low (*L*) FRET value are $k_{H\to L}^{est}\;\Delta t=0.016$ in 1500 ≲ *t* ≲ 4000, and $k_{H\to L}^{est}\;\Delta t=0.026$ in 5700 ≲ *t* ≲ 8700. Thus, in (*K* = 3)-model the two time intervals, originally generated by using the same kinetic parameter ($k_{H\to L}^{\left(2\right)}\;\Delta t=0.025$), are determined to be distinct from each other (blue trace in Fig 3A(v)). By contrast, in (*K* = 2)-model, $k_{H\to L}^{est}\;\Delta t=0.020$ was estimated over these two time intervals. This type of statistical error is unavoidable for a small *T*_*obs*_. A more systematic evaluation on the accuracy of the algorithm as a function of *T*_*obs*_ and transition rate between distinct internal states will be discussed in the next section.

To assess the accuracy of the best model $x_{\left(K^{*}\right)}^{\text{model}}$ predicted by VB-DCMM against the solution ***x***, the following overlap function can be used.

$$\begin{array}{c} \chi =\frac{1}{T-1}\sum_{t=1}^{T-1}\delta_{x\left(t\right),x_{\left(K^{*}\right)}^{\text{model}}\left(t\right)} \end{array}$$ (3)

where *δ*_*i*,*j*_ is the Kronecker delta and *T* = *T*_*obs*_/Δ*t* is the total number of data in the traces (Δ*t* denotes the temporal resolution of the data). For 100 synthetic time traces, generated under the identical parameters used for producing the time trace in Fig 3A, we found that *χ* ≈ 0.9 on average (Fig 3C). Note, however, that *x*(*t*), only available for the case of “synthetic data”. Thus, to assess the accuracy of our method against a real time trace from SM experiments, we devised other metrics.

For a given time trace with dynamic disorder, our algorithm quantifies the kinetic features of the time trace in terms of the transition rate between the observable states *a* and *b* within the *μ*-th internal state $k_{a\to b}^{\left(\mu \right)}$ and the transition rate from the *μ*-th internal state to *ν*-th internal state *γ*^(*μ*)→(*ν*)^ (1 ≤ *μ*, *ν* ≤ *K*, 1 ≤ *a*, *b* ≤ *N*. Here, *μ* is the index for internal state whereas *a* and *b* are indices for observable (In FRET displaying low/high two state transitions, these states correspond to the low and high FRET values). *K* is the total number of hidden internal states, and *N* denotes the total number of observables). To be able to extract the information of multiple internal states reliably from a time trace using VB-DCMM, two general conditions are required for the time trace being analyzed.

1. A large time scale separation should be present in the kinetics within each internal state, i.e., $k_{a\to b}^{\left(\mu \right)}$ and $k_{a\to b}^{\left(\nu \right)}$ (*μ* ≠ *ν*) should be disparate.
2. There should be a clear time scale separation between intra-basins and inter-basin transitions (i.e., *τ*_*conf*_ and *τ*_*int*_). More precisely, the intra-basin transition probability $k_{a\to b}^{\left(\mu \right)}\;\Delta t$ should be much *greater* than the transition probability from the *μ*-th to any other internal state ∑_*ν*≠*μ*_ *γ*^(*μ*)→(*ν*)^ Δ*t* (= 1 − *γ*^(*μ*)→(*μ*)^ Δ*t*).

To substantiate the above-mentioned conditions 1 and 2, we define two metrics *D*_conf_ and *D*_int_, which compute the average Hamming-like distances between the distinct rate constants extracted from a given time trace using VB-DCMM analysis:

$$D_{\text{conf}}=\frac{2}{K\left(K-1\right)}\sum_{\begin{array}{c} \mu ,\nu =1\\ \mu >\nu \end{array}}^{K}\frac{1}{N\left(N-1\right)}\sum_{\begin{array}{c} a,b=1\\ a\neq b \end{array}}^{N}\left|\log_{2}\frac{k_{a\to b}^{\left(\mu \right)}}{k_{a\to b}^{\left(\nu \right)}}\right|$$ (4)

and

$$D_{\text{int}}=\frac{1}{K}\overset{K}{\underset{\mu =1}{\sum }}\frac{1}{N\left(N-1\right)}\sum_{\begin{array}{c} a,b=1\\ a\neq b \end{array}}^{N}\left|\log_{2}\frac{k_{a\to b}^{\left(\mu \right)}}{\sum_{\nu \neq \mu }\gamma^{\left(\mu \right)\to \left(\nu \right)}}\right|.$$ (5)

*D*_conf_ measures the dissimilarity between distinct internal states in terms of the intra-basin transition rates. Two distinct internal states (*μ*, *ν* (*μ* ≠ *ν*)) can be better discerned if the intra-basin transition rate of one internal state (say, $k_{a\to b}^{\left(\mu \right)}$) differs greatly from that of other internal state ($k_{a\to b}^{\left(\nu \right)}$), so that $|\log_{2}(k_{a\to b}^{\left(\mu \right)}/k_{a\to b}^{\left(\nu \right)})|$ is maximized. *D*_int_ measures the average number of intra-basin transitions in each internal state using the ratio between the transition probabilities, $k_{a\to b}^{\left(\mu \right)}\;\Delta t$ and ∑_*ν*≠*μ*_ *γ*^(*μ*)→(*ν*)^Δ*t* (= 1 − *γ*^(*μ*)→(*μ*)^ Δ*t*). A greater *D*_int_ ensures a large time scale separation in dynamics between intra-basin and inter-basin transitions, which improves the reliability of our method to decode the internal state from a given time trace. In general, *D*_int_ or *D*_conf_ shows a good correlation with 〈*χ*〉 (see below); thus, one can use (*D*_int_, *D*_conf_) to assess the accuracy of predicted internal states. Note that the metrics *D*_int_ and *D*_conf_ can be estimated for real data, while 〈*χ*〉 can be calculated only against the synthetic data. Since there is a good correlation between (*D*_int_, *D*_conf_) and *χ*, one can evaluate (*D*_int_, *D*_conf_), alternative to *χ*, to assess the reliability of a predicted result of $x_{\left(K^{*}\right)}^{\text{model}}\left(t\right)$.

To be more concrete, we applied VB-DCMM algorithm to analyze synthetic data generated with *N* = 2 (transitioning between high and low FRET values) and *K* = 2 (two internal states; *μ* = 1 and 2) under various scenarios.

**Fig 4:**
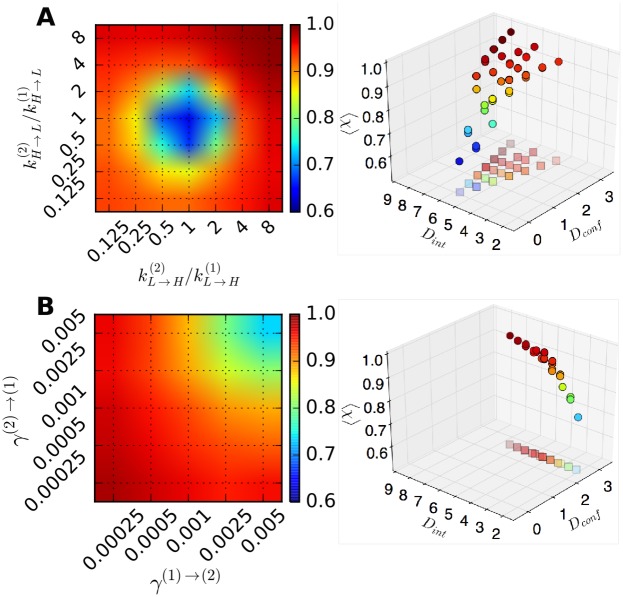
Accuracy of VB-DCMM in detecting internal states under various conditions of $k_{L↔H}^{\left(\mu \right)}$ and *γ*^(1)↔(2)^ with *T*_*obs*_/Δ*t* = 8800. (A) The color bar denotes the accuracy of analysis in terms of 〈*χ*〉 under varying $k_{L\to H}^{\left(2\right)},k_{H\to L}^{\left(2\right)}$ with *K* = 2, $k_{L\to H}^{\left(1\right)}\Delta t=0.05$, $k_{H\to L}^{\left(1\right)}\Delta t=0.05$, and *γ*^(1)→(2)^Δ*t* = *γ*^(2)→(1)^Δ*t* = 0.001. (B) 〈*χ*〉 under varying *γ*^(1)→(2)^ and *γ*^(2)→(1)^ with *K* = 2, $k_{L\to H}^{\left(1\right)}\Delta t=k_{H\to L}^{\left(1\right)}\Delta t=0.05,k_{L\to H}^{\left(2\right)}\Delta t=0.00625,k_{H\to L}^{\left(2\right)}\Delta t=0.0125$. 〈*χ*〉 was calculated by averaging over the results from analysis of 100 traces in each condition. The panels on the right show the relation between the value of 〈*χ*〉 and pairs of *D*_int_ and *D*_conf_ values which are evaluated at varying kinetic parameters. Results from the analysis over the data with the same parameters but different length of time trace (or different number of data points *T*_*obs*_/Δ*t* = 2200, 4400) are provided in S4 Fig.

**Fig 5:**
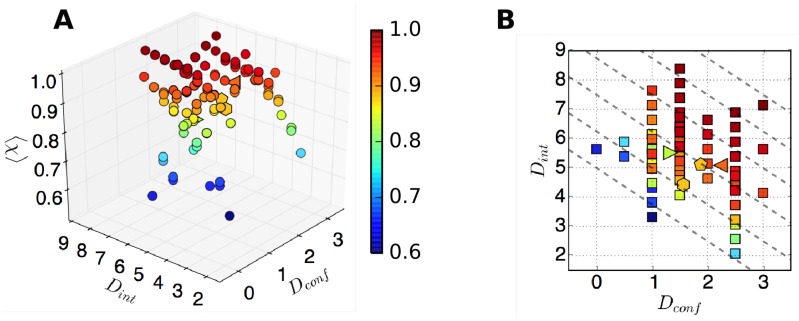
Average accuracy of internal state detection as a function of *D*_conf_, and *D*_int_. To construct this diagram, we employed various synthetic data in Fig 4 (circle, two internal states (*K* = 2), two FRET states (*N* = 2)), S5 Fig (left triangle, *K* = 3, *N* = 2), and S6 Fig (hexagon, *K* = 2, *N* = 3). The right triangle symbol denotes the result from the similar analysis shown in S5 Fig with *K* = 3 but with smaller relative differences in the transition rates, *k*’s. Pentagon represents the result obtained with *K* = 4 and *N* = 2. (A) Color code denotes the accuracy of internal states predictions in terms of 〈*χ*〉, averaged over 100 traces for each condition. (B) The dashed lines corresponding to Δ = *D*_conf_ + 0.8*D*_int_ = 4, 5, … 9 are overlaid on the 2-D scatter plot of 〈*χ*〉(*D*_conf_, *D*_int_) calculated in (A).

- We fixed the transition rates in the state *μ* = 1 as $k_{L\to H}^{\left(1\right)}\;\Delta t=k_{H\to L}^{\left(1\right)}\;\Delta t=0.05$, and varied the rates associated with the state *μ* = 2 over the range of $0.125\leq k_{L\to H}^{\left(2\right)}/k_{L\to H}^{\left(1\right)},\;k_{H\to L}^{\left(2\right)}/k_{H\to L}^{\left(1\right)}\leq 8$ (Fig 4A, left). For the interconversion probability between the two internal states we set *γ*^(1)→(2)^ Δ*t* = *γ*^(2)→(1)^ Δ*t* = 0.001. The accuracy of the model prediction (〈*χ*〉, Eq (3)) is on average greater than 0.9 as long as the transition rates $k_{L↔H}^{\left(\mu \right)}$ and $k_{L↔H}^{\left(\nu \right)}$ (*μ* ≠ *ν*) differ more than the factor of 4. Note that in Fig 4A (left), the value of 〈*χ*〉 is greater for $k_{L\to H}^{\left(2\right)}/k_{L\to H}^{\left(1\right)}$, $k_{H\to L}^{\left(2\right)}/k_{H\to L}^{\left(1\right)}\gg 1$ than for $k_{L\to H}^{\left(2\right)}/k_{L\to H}^{\left(1\right)}$, $k_{H\to L}^{\left(2\right)}/k_{H\to L}^{\left(1\right)}\ll 1$; this is because a statistically sufficient number of transitions make the detection of internal states more reliable. In contrast, when $k_{L\to H}^{\left(2\right)}/k_{L\to H}^{\left(1\right)}$, $k_{H\to L}^{\left(2\right)}/k_{H\to L}^{\left(1\right)}≃1$, i.e. when the kinetics inside the two internal states are essentially identical, it is difficult to discern the two internal states. In this case, *K* = 1 instead of *K* = 2 is effectively the correct number of internal states. Indeed, when *K* = 1 is assumed (i.e., assuming true internal state *x*(*t*) = 1 for all *t* in Eq (3)), the re-calculated 〈*χ*〉 is close to 1 (see S2 Fig).
- To explore the effect of interconversion between distinct internal states on the performance of algorithm, we generated synthetic data with $k_{L\to H}^{\left(1\right)}\;\Delta t=k_{H\to L}^{\left(1\right)}\;\Delta t=0.05$, $k_{L\to H}^{\left(2\right)}/k_{L\to H}^{\left(1\right)}=0.125$, and $k_{H\to L}^{\left(2\right)}/k_{H\to L}^{\left(1\right)}=0.25$ by, this time, varying *γ*^(1)→(2)^ Δ*t* and *γ*^(2)→(1)^ Δ*t* = 0.00025 ∼ 0.005 (Fig 4B, left). The results clearly show that the case with smaller *γ*^(*μ*)→(*ν*)^ results in a higher 〈*χ*〉, which is expected because each internal state can have more number of transitions in the traces ***o*** when the interconversion is slower (Fig 4B). Re-plotting 〈*χ*〉 as a function of *D*_conf_ and *D*_int_ reveals clear dependence of the accuracy on *D*_int_ (Fig 4B, right). Similar trends are observed for other conditions of $k_{L\to H}^{\left(2\right)}/k_{L\to H}^{\left(1\right)}$ and $k_{H\to L}^{\left(2\right)}/k_{H\to L}^{\left(1\right)}$ (S3 Fig).
- Analyses on synthetic data generated using the same input parameters with those in Fig 4, but with a different number of data points in each trace, *T*_*obs*_/Δ*t* = 4400, and 2200 (S4 Fig) show a similar trend as observed in Fig 4 with *T*_*obs*_/Δ*t* = 8800 but with slightly smaller 〈*χ*〉 values.
- Extension of VB-DCMM algorithm to a more complicated case for *K* > 2 (S5 and S14 Figs) or *N* > 2 (S6 and S14 Figs) is straightforward. Application of VB-DCMM to a trajectory in which each internal state trajectory has different *N* is also straightforward (S7 Fig). In the latter case, the data is analyzed by assuming that all internal states have the same number of possible observables, *N*; but the analysis would indicate that transition associated with a small transition rate is essentially disallowed. In all situations considered for various *K* and *N*, VB-DCMM can be used for the reliable recovery of the sequence of true internal states.
- Analyses of synthetic traces show that the accuracy of the algorithm improves with both *D*_conf_ and *D*_int_ (Fig 4 right panels and Fig 5). Thus, these two metrics allow one to judge the reliability of the information on internal states extracted from a given time trace. Alternatively, a single parameter *D*_tot_(= *D*_conf_ + *αD*_int_) with an empirically acquired coefficient *α* ≈ 0.8) can be used to judge the reliability of the extracted information. Note that 〈*χ*〉 remains similar as long as *D*_tot_ remains constant (Fig 5B). Hence, when 〈*χ*〉 is plotted against *D*_tot_, all synthetic data generated using different parameters approximately collapse onto a single universal curve (S8 Fig).
- There are multiple ways of assessing the efficacy of VB-DCMM in decoding the internal states. In addition to 〈*χ*〉, *D*_conf_, *D*_int_, and *D*_tot_ as the possible measures for the assessment, one can also use the statistical property that the dwell times of homogeneous Markov process satisfies $\sqrt{\langle \tau^{2}\rangle -{\langle \tau \rangle }^{2}}/\langle \tau \rangle ∼1$ (see **SI** for details).

### Application of VB-DCMM on H-DNA data

**Fig 6:**
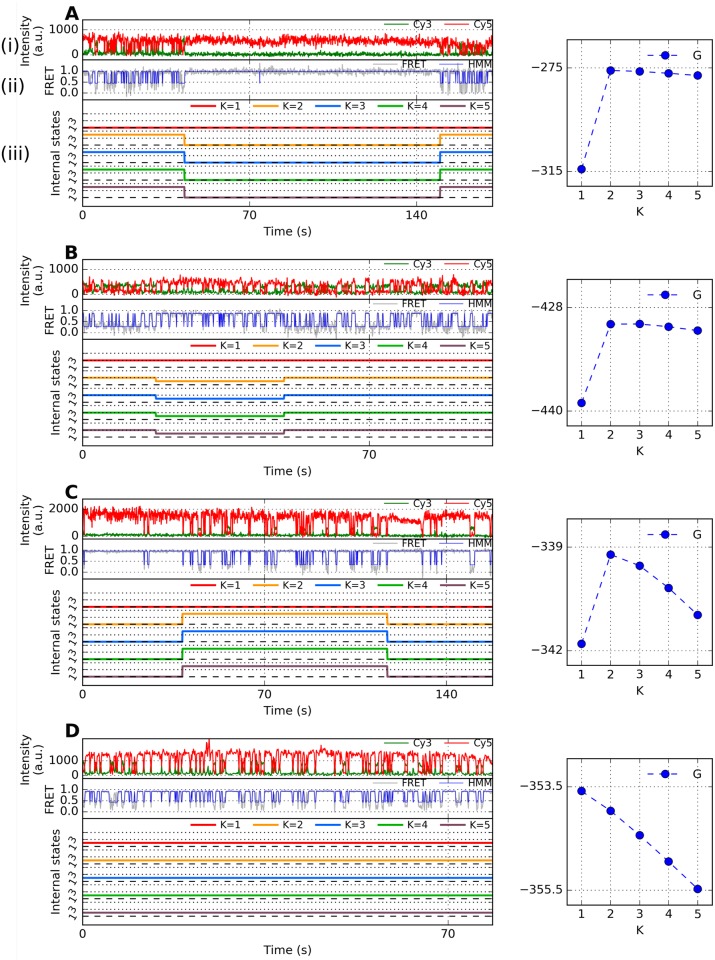
Representative time traces of H-DNA at [Na^+^] = 100 mM and their analysis. (A) (i) Fluorescence signal and (ii) their FRET state. (iii) Internal states estimated for *K* = 1, 2, …, 5. Right panel shows *G*(*K*) (blue circle) where *K*_*obs*_ specifies the number of detected internal states in individual traces (blue). (B, C, D) Other representative time traces and their *G*(*K*) obtained under the same experimental condition.

Now, to analyze the duplex-triplex transitions of H-DNA (Fig 6), we obtain ***o*** by filtering the noise from FRET signal (Fig 6(ii), blue line) and apply the VB-DCMM algorithm to decode the hidden internal state in the signals. Fig 6(iii) shows time series of internal state, $x_{\left(K\right)}^{\text{model}}$, calculated from the VB-DCMM by varying *K* from 1 to 5. It is of note that the number of actually observed internal states in the $x_{\left(K\right)}^{\text{model}}$ for a given input parameter *K* does not change after some *K*_*obs*_(≤ *K*) (*K*_*obs*_ = 2 (Fig 6A), 2 (Fig 6B), 2 (Fig 6C), and 1 (Fig 6D)). (See also other time traces of synthetic data and H-DNA analyzed in SI: S1A Fig (*K*_*obs*_ = 1), S9A Fig (*K*_*obs*_ = 3), S9B Fig (*K*_*obs*_ = 2), S9C Fig (*K*_*obs*_ = 3), S9D Fig (*K*_*obs*_ = 3), S10A Fig (*K*_*obs*_ = 4), S10B Fig (*K*_*obs*_ = 2), S10C Fig (*K*_*obs*_ = 2), S10D Fig (*K*_*obs*_ = 3), and S12 Fig (*K*_*obs*_ = 3)). A similar behavior is also observed when analyzing data using the variational Bayes Gaussian mixture model [48].

To account for the contribution due to degeneracy in labeling the internal states, log *K*! term is conventionally considered in formulating the evidence function *F*(*K*) (See **SI** for the details); however, in our problems, the actual number of degeneracy in labeling internal states should be ${}_{K}C_{K_{obs}}\times K_{obs}!$ instead of *K*!. Therefore, we replace the log *K*! term in *F*(*K*) with log [*K*!/(*K* − *K*_*obs*_)!], and considered a modified evidence function, *G*(*K*), to identify an optimal *K* for a given time trace:

$$G\left(K\right)\equiv F\left(K\right)-\log(K-K_{obs})!$$ (6)

*G*(*K*) shows a clear peak, allowing us to identify the optimal *K*(= K*) with ease (blue circles on the right side of Figs 6, S9 and S10). Use of *G*(*K*) instead of *F*(*K*) in analyzing synthetic data does not alter K* (S12 and S14B Figs).

Among the time traces of H-DNA, traces with more than 3 interconversions between distinct internal states, which enables us to estimate *γ*^(*μ*)→(*ν*)^, are rare, especially when [Na^+^] = 100 mM; thus it is not feasible to get a statistically meaningful scatter plot of (*D*_conf_, *D*_int_) (see S23D, S23E and S23F Fig); however, for those displayed in S23D, S23E and S23F Fig, 〈*D*_tot_〉 ≈ 7 suggests that *χ* ≳ 0.9 (from Fig 5). Therefore, at least the intra-basin rate constants extracted from H-DNA data using VB-DCMM are reliable.

Time traces that have *τ*_*int*_ comparable to experimental observation time ($\tau_{int}\approx T_{obs}$) would exhibit on average no or only a single transition event between distinct internal states. Indeed, we find that only a subset of total number of internal states is sampled by individual time traces due to the limited observation time. For instance, at [NaCl] = 100 mM, our analysis identified K* ≤ 2 in 265 out of 269 traces, and that only 4 time traces display K* > 2 (S11 Fig). Therefore, in order to identify the internal states present in the transition dynamics of H-DNA, clustering analysis is required against the whole ensemble of time trajectories. We provide the procedure of clustering analysis and results in details in the following section.

### Clustering H-DNA data

**Fig 7:**
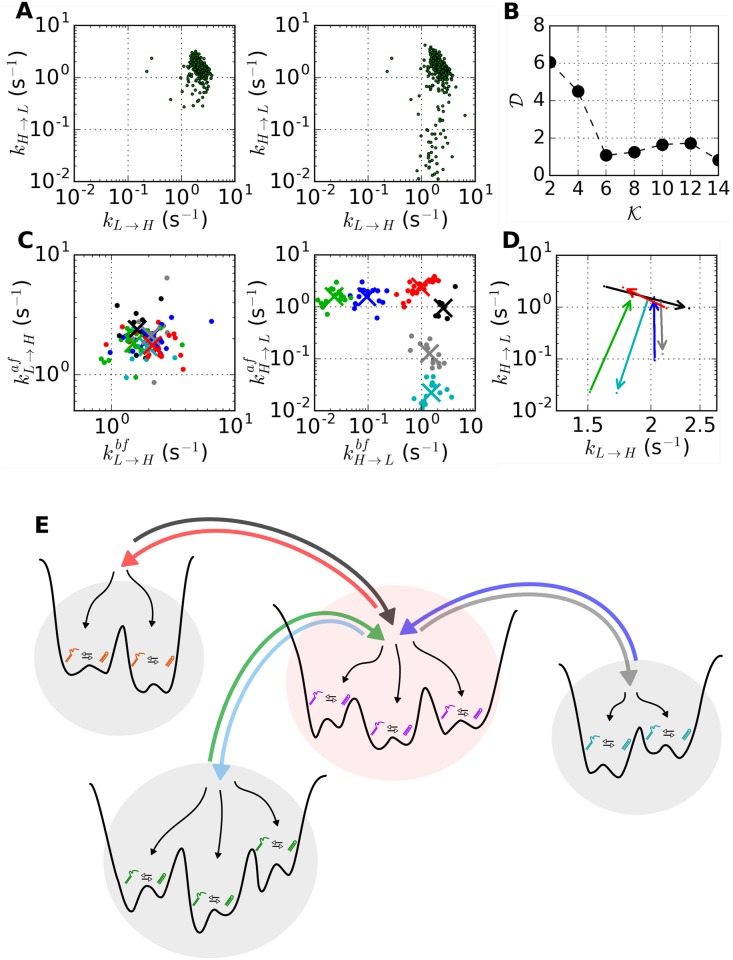
Clustering H-DNA data at [Na^+^] = 100 mM condition. (A) The scatter plots of (*k*_*L*→*H*_, *k*_*H*→*L*_) before (left) and after (right) applying VB-DCMM from [Na^+^] = 100 mM data. (B) The “average pairing distance” D(K) as a function of the number of clusters (K)(see Methods). The minimum value of D(K) is found at K=6. (C) Left: the scatter plot of clustered data projected on $\left(k_{L\to H}^{\text{bf}},k_{L\to H}^{\text{af}}\right)$ plane. Right: scatter plot of clustered data projected on $\left(k_{H\to L}^{\text{bf}},k_{H\to L}^{\text{af}}\right)$ plane. The data belonging to different clusters are depicted in different colors, and the centroid of each cluster is marked with the × symbol. Total 98 data points were used for analysis. (D) The result of the above clustering is represented using 6 kinetic arrows which represent the centroids of each cluster represented in (*k*_*L*→*H*_, *k*_*H*→*L*_) plane. The starting point of the arrow is ($\langle k_{L\to H}^{(\mu ),\text{bf}}\rangle$, $\langle k_{H\to L}^{(\mu ),\text{bf}}\rangle$) whereas the ending point of the arrow is ($\langle k_{L\to H}^{(\mu ),\text{af}}\rangle$, $\langle k_{H\to L}^{(\mu ),\text{af}}\rangle$), where the superscript *μ* represents the index of each cluster *μ* = 1, 2, … 6. The colors used for depicting kinetic arrows are consistent with the data points in (C). (E) A schematic of the conformational landscape of H-DNA.

VB-DCMM algorithm allows us to decompose individual H-DNA time traces with dynamic disorder into multiple “components”, each of which should satisfies the property of *homogeneous Markov chain*. In order to understand the structure of conformational space of H-DNA, the ensemble of components acquired from the VB-DCMM analysis should be clustered into the same kind. To this end, we produce scatter plots of (*k*_*L*→*H*_, *k*_*H*→*L*_), representing the kinetic property of the ensemble of time traces, using the transition rates estimated for individual time traces. The scatter plots of (*k*_*L*→*H*_, *k*_*H*→*L*_) were calculated for the ensemble of H-DNA time traces (i) before (Fig 7A, left) and (ii) after decomposing the individual heterogeneous time traces retaining multiple components into the homogeneous ones (Fig 7A, right). The scatter plot of (*k*_*L*→*H*_, *k*_*H*→*L*_) after the decomposition has a greater dispersion, which is expected since a data point (*k*_*L*→*H*_, *k*_*H*→*L*_) for a time trace with dynamic disorder is a mixture of $\left(k_{L\to H}^{\left(\mu \right)},k_{H\to L}^{\left(\mu \right)}\right)$ with *μ* = 1, 2, … *K*. In the presence of clear distinction between internal states (*μ* ≠ *ν*), the clustering of $\left(k_{L\to H}^{\left(\mu \right)},k_{H\to L}^{\left(\mu \right)}\right)$ would be straightforward, which is indeed the case for the synthetic data (S13A Fig). However, for the H-DNA data, even after the decomposition, the clustering of data on (*k*_*L*→*H*_, *k*_*H*→*L*_) plane (Fig 7A) is not that clear.

To improve the quality of clustering, we extended the clustering of the kinetic data to a higher dimension by considering the kinetic information of internal states that are contiguous (kinetically linked) along time traces. To be specific, for a time trace exhibiting a transition from the *μ*-th to *ν*-th internal state (*μ* ≠ *ν*), one can consider that the inter-basin transition has occurred from the time interval represented by its pair of kinetic rate $\left(k_{L\to H}^{\text{bf}},k_{H\to L}^{\text{bf}}\right)\left[=\left(k_{L\to H}^{\left(\mu \right)},k_{H\to L}^{\left(\mu \right)}\right)\right]$ to the next time interval represented by $\left(k_{L\to H}^{\text{af}},k_{H\to L}^{\text{af}}\right)\left[=\left(k_{L\to H}^{\left(\nu \right)},k_{H\to L}^{\left(\nu \right)}\right)\right]$, where the superscripts, ‘bf’ and ‘af’ denotes ‘before’ and ‘after’ the transition, respectively. Thus, instead of (*k*_*L*→*H*_, *k*_*H*→*L*_), a clustering at a higher dimension can be carried out by measuring the Euclidean distance between a pair of the four-dimensional (4-dim) arrays, $\left(\log k_{L\to H}^{\text{bf}},\log k_{H\to L}^{\text{bf}},\log k_{L\to H}^{\text{af}},\log k_{H\to L}^{\text{af}}\right)$.

In order to cluster the 4-dim arrays we used the k-means clustering algorithm. Application of the algorithm to the H-DNA data at [Na^+^] = 100 mM reveals that the average pairing distance, D(K) (see Methods), is minimized when the number of clusters is 6 (K=6), namely, the model with 6 clusters provides the best interpretation of the data (Fig 7B). Although the model with 14 clusters shows a smaller D, we selected K=6 as the best solution, since for K=14 each of 12 clusters out of 14 has less than 10 data points, which makes the result of clustering statistically less significant (S16 Fig). This results remain qualitatively identical when *L*1 distance (so called “city block” distance) was used instead of “square-euclidean” distance (S19 Fig). Furthermore, the clustering algorithm using “affinity propagation” [58], which considers all the data points as possible exemplars (analogous to centroids in k-means clustering method) and iteratively exchanges messages between them, also gives qualitatively identical results, confirming the robustness of the conclusion on H-DNA dynamics obtained from VB-DCMM and k-mean clustering (see S20 Fig).

We present the result of clustering either (i) by projecting it on the two separate kinetic planes, $\left(k_{L\to H}^{\text{bf}},k_{L\to H}^{\text{af}}\right)$ and $\left(k_{H\to L}^{\text{bf}},k_{H\to L}^{\text{af}}\right)$, which visualize the inter-basin transitions in terms of variable *L* → *H* and *H* → *L* transition rates (see Fig 7C), or (ii) by using “interconversion arrows” linking the kinetic rates of two internal states, $\left[\left(\log k_{L\to H}^{\text{bf}},\log k_{H\to L}^{\text{bf}}\right)\to \left(\log k_{L\to H}^{\text{af}},\log k_{H\to L}^{\text{af}}\right)\right]$ on the (*k*_*L*→*H*_, *k*_*H*→*L*_) plane (Fig 7D). Note that in the scatter plot visualized with $\left(k_{H\to L}^{\text{bf}},k_{H\to L}^{\text{af}}\right)$, the distinction between different clusters is clear (the right panel of Fig 7C). Furthermore, for a system in equilibrium or at least near equilibrium, the interconversion between two internal states, say *μ* and *ν*, should occur in both directions, i.e., *μ* → *ν* and *ν* → *μ*. In the representation (i), a symmetry of $\left(k_{a\to b}^{\left(\mu \right)},k_{a\to b}^{\left(\nu \right)}\right)=\left(k_{a\to b}^{\left(\nu \right)},k_{a\to b}^{\left(\mu \right)}\right)$ is expected in the both panels of Fig 7C; and in the representation (ii), the “arrows”, amounting to the kinetic connectivity between distinct internal states, should be bi-directional. The symmetry of the data plotted in Fig 7C or the bidirectionality of the kinetic arrows confirms the condition of detailed balance being satisfied in the system in equilibrium. Fig 7D depicts 6 kinetic arrows (3 pairs of reversible kinetic arrows) connecting the centroids of $\left(\log k_{L\to H}^{\text{bf}},\log k_{L\to H}^{\text{af}}\right)$ or $\left(\log k_{H\to L}^{\text{bf}},\log k_{H\to L}^{\text{af}}\right)$ data.

Application of the above clustering method to synthetic data with *K* = 3, *N* = 2 (S13 Fig) is straightforward. To check the efficacy of clustering method for a more complicated case, we have tested with synthetic data generated with *K* = 4, *N* = 4, i.e. when there are as many as 4 observable states in each internal state (S14 and S15 Figs). In the case with 4 observable states, total 12 possible intrabasin transitions are conceivable. Thus, the dimension of the array associated with interbasin transition is 24. As long as there is a clear time scale separation, it is expected that the pairing distance D(K=12) shows minimum as there are 12 connection paths between 4 internal states. Indeed, D(K) is minimized at K=12 (S15A Fig).

Lastly, it is noteworthy that the clustering method presented here is not limited to data analysis for systems in equilibrium, but can be extended to systems in nonequilibrium steady state [59] where the individual state-to-state kinetic transition rate is well defined using the reversible Markov process although the condition of detailed balance is no longer anticipated [60, 61]. The symmetry of data point and bidirectionality of kinetic arrows as in Fig 7C and 7D are still of use to cluster the kinetic information generated from a system in nonequilibrium steady states.

### Folding energy landscape of H-DNA

We classified the “components” of a similar kinetic pattern (*k*_*L* → *H*_, *k*_*H* → *L*_) obtained from VB-DCMM into a single cluster which represents a kinetic path linking two independent basins of attraction (or internal states). For example, the kinetic paths in Fig 7D can be best understood by hypothesizing 4 internal states (four basins) linked by 6 kinetic paths. Thus, the conformational transition landscape of H-DNA at [Na^+^] = 100 mM condition consists of 4 internal states with 3 reversible kinetic paths being established as illustrated in Fig 7E. At lower salt concentrations ([Na^+^] = 50 mM (S17 Fig) and [Na^+^] = 26 mM (S18 Fig)), H-DNA transitions slow down and the dispersion of data also increases; however, the overall structure of conformational landscape of H-DNA remains unchanged from the picture suggested in Fig 7E; thus, there is a central superbasin to which three other superbasins are kinetically connected (S17 and S18 Figs).

### Contributions of our work

In comparison to other pre-existing methods, the advantage of our VB-DCMM in decoding dynamic disorder from a given trajectory is highlighted as follows:

(1) Dynamic disorders in single molecule time trajectories are modeled using DCMM by assuming the presence of hidden internal states. While Aggregated Markov Model (AMM), which has been adopted in ion-channel community for time trace analysis of varying current [62–76], can be employed to analyze our data with dynamic disorder, DCMM is better in correctly decoding dynamic disorder than AMM. We found that AMM is prone to overpredict the transition between kinetic patterns (S25 Fig). Our method is more suitable to the data showing persistent dynamic patterns by suppressing unwanted frequent transition between kinetic patterns. Detailed explanations of connection and quantitative comparison between DCMM and AMM are provided in SI and S25 Fig.

(2) In this paper, Bayesian version of DCMM was developed by using variational Bayes (VB) method, which enabled us to determine the number of internal states straightforwardly. Although Bayesian version of DCMM using Markov chain Monte Carlo (MCMC) method has previously been developed for the credit portfolio modeling [77], the idea of Bayesian inference in ref. [77] was used only for the purpose of calculating a posterior distribution of model parameters. To determine the number of hidden states corresponding to the internal states in this study, the authors in ref. [77] used the economic cycle fluctuation model, instead. Our study combining VB with DCMM (i) can determine the number of internal states in a more objective fashion, (ii) offers intuitive way to incorporate prior knowledge, and (iii) is computationally more efficient than MCMC (See SI for details).

(3) We tested VB-DCMM under various conditions, by varying the kinetic rates, the number of observables, the number of hidden states, and prior parameters. New metrics were also devised to quantify the performance of algorithm systematically.

(4) Finally the connection paths (kinetic arrows) between internal states of H-DNA are clustered by using the kinetic components extracted from VB-DCMM and by applying k-means clustering algorithm to high dimensional arrays.

To recapitulate, our entire process of analyzing single molecule data is composed of three stages: (i) noise-filtering using HMM; (ii) decomposition of heterogeneous time traces into the homogeneous components using VB-DCMM; (iii) clustering the decomposed components into the same cluster.

In principle, this three-stage analysis can be made more systematic by combining the noise-filtering and clustering procedure with VB-DCMM. To be more specific, (1) The noise-filtering of observable trace (*o*_*n*_) is processed, independently from the main VB-DCMM algorithm, by using HMM, which has been proved to be reliable in noise-filtering [56], and the maximum number of observables (*N*) are predetermined as an input parameter. Current version of algorithm can be further automated by combining with the Bayesian version of HMM [49], which can determine the number of observables while filtering the noise in data (See Fig 2D). The resulting model will have a similar structure with the modified factorial HMM [46, 47]. (2) The heterogeneous components identified from individual time traces are clustered separately from our main algorithm. It would be also desirable to unify the post-processing step (clustering) with VB-DCMM using empirical Bayes method which has been applied recently to analyze single molecule data [54, 55].

However, it should also be noted that a blind integration of noise-filtering and clustering steps inevitably complicates the implementation of VB-DCMM, as more number of prior parameters are ought to be decided by users. For example, Bayesian implementation of HMM for noise filtering demands manual determination of additional *N*(*N* + 5) prior-parameters [49]. Compared to this, currently VB-DCMM requires users to pre-determine only one prior parameter which characterizes the final transition rate matrix, ***A*** (see the subsection: *Selection of prior parameters* in SI). Moreover, the integration of other methods will obscure the flow of analysis, making it difficult to identify an error-causing step. Keeping each step in the algorithm separate makes the integration of VB-DCMM to other applications more transparent (for example, if noise-filtering by HMM is unsuccessful, other advanced method can be employed [49]). We leave it as our future work to develop an algorithm that integrates the above-mentioned three procedures (noise-filtering, VB-DCMM, and clustering) without increasing complexity or obscuring the flow of analysis.

In decoding SM FRET data, the most notable difference of our VB-DCMM from the previous studies employing the probabilistic models such as maximum likelihood and Bayesian statistics is that VB-DCMM explicitly considers the situation that transition rates can change from one time interval to another within individual time traces. The previous studies [49–51, 56, 78–80] assumed that the transition rates were constant within individual time traces. Also, currently, VB-DCMM is applicable to window-averaging FRET trajectories. It will be of great interest to extend VB-DCMM to analyzing time trajectories in which arrival times for individual photons are available. VB-DCMM is particularly powerful when there is a separation in time scales between *τ*_*int*_ and *τ*_*conf*_.

### Concluding remarks

While the notion of dynamical heterogeneity or broken ergodicity seems better recognized in the research field of nucleic acids [81] than in proteins, which likely arises from more homopolymer-like nature of building block of nucleotides [82], biomolecules in general can have a rugged folding landscape with many local basins of attraction and kinetic barriers with varying heights [83]. Conformational dynamics of biomolecules on rugged landscapes can be heterogeneous, which gives rise to static or dynamic disorder depending on the time scale of observation or the height distribution of kinetic barrier. The presence of heterogeneity or disorder among individual molecules, unveiled by *in vitro* SM experiments could be surprising at first sight; however, it is also important to note that the general hypotheses in the conventional molecular biology towards a single native state have been put forward based on the observations from ensemble experiments where the heterogeneity, if any, is usually masked by the process of ensemble averaging. Given that the complexity of a molecular system increases with the system size (*N*_sys_) as $∼e^{N_{\text{sys}}}$ [29], it should not be too surprising to find such disorder in biomolecules in itself. Cells are equipped with molecular chaperones that can tame misfolding-prone biomolecules with rugged landscapes [84–87]; thus the principle of optimization in biology, if it fails at the level of a molecule in isolation, can be extended further to the molecular system including its environmental factors.

It is not easy to elucidate the molecular origin of disorder in a conclusive manner; yet, it has recently been suspected that interactions of biomolecules with cofactor such as ATP and multivalent metal-ions could be the microscopic causes for those molecules exhibiting dynamical heterogeneity [12, 13, 16, 88, 89]. Modulating the concentration of Mg^2+^ ions from high to low and again to high induced inter-conversions of dynamic patterns in equilibrium conformational fluctuations of *T*. ribozyme [12] and Holliday junctions [13]. Distinct velocities of ATP-empowered individual RecBED helicase motors, which can move progressively along dsDNA by unwinding it into two separate strands, can be reset by introducing a long pause by halting the supply of ATP. For the time trajectories of biomolecules displaying quenched disorder, a method to analyze such data was proposed using a concept from glass physics [13]. Here, to deal with more general scenarios, we have developed a method to analyze single molecule time traces with dynamic disorder.

As demonstrated by testing the VB-DCMM algorithm on synthetic data, the algorithm is quite accurate in decoding dynamic disorder as long as a time trajectory of interest contains multiple time intervals, each of which display kinetic pattern distinct from others. When a clear separation in timescale is present between two distinct kinetic patterns, large value of *D*_conf_, *D*_int_, and *D*_tot_ would be acquired.

While we developed the VB-DCMM algorithm primarily to analyze dynamic disorder in duplex-triplex transitions of H-DNA, the method is applicable to any data in the form of one-dimensional time series with multiple transitions. Together with a further technical advance in SM, which eliminates experimental artifacts as well as extends the measurement time, our algorithm developed here will contribute to better understanding of biomolecules that display heterogeneous dynamics.

## Methods

### Generation of synthetic data

Internal state sequence ***x*** was generated by using Monte Carlo method with a constant transition matrix (homogeneous Markov chain model). The observable sequence ***o*** was generated by using the same method but with the transition matrix that was defined at each time *t* based on the internal state *x*(*t*). Finally, Gaussian noise was added on ***o*** to produce ***o***_*n*_.

### Single-molecule FRET measurements to monitor duplex-triplex transitions of H-DNA

We purchased triplex forming oligonucleotides from Integrated DNA Technologies (Coralville, IA, USA). The oligonucleotides were dissolved in T50 buffer solution (10 mM Tris-HCl, 50 mM NaCl, pH = 7.5) and were heated beyond the melting temperature of DNA duplex (∼ 90°C), and slowly cooled down on a heat block to room temperature over 8 hour to properly hybridize them. The DNA prepared as such is called “H-DNA” here. The sequences of the triplex forming strands (purine-rich and pyrimidine-rich) are: Purine-rich strand: 5’ AAG AAG AAG AAG AAG (Cy5) TGG CGA CGG CAG CGA (Biotin) 3’, Pyrimidine-rich strand: 5’ TCG CTG CCG TCG CCA CTT CTT CTT CTT CTT TTT TCT TCT TCT TCT TCT TC (Cy3) 3’. In the purine-rich strand, the biotin at 3’ terminus is used to attach the H-DNA molecule to a neutravidin-coated cover-glass. The Cy3 and Cy5 dyes in the H-DNA molecule correspond to a donor and an acceptor for FRET measurements, respectively. In order to observe the transition between folded triplex and unfolded DNA, we used the reaction buffer containing 50 mM HEPES(Sigma-Aldrich) and various concentrations of Na^+^ (26, 50, 100 mM). These buffer solutions also contained 2 mM trolox, 10% glucose and gloxy for single-molecule fluorescence experiments. We utilized a home-made TIRF (Total Internal Reflection Fluorescence) microscope to measure the FRET efficiency between donor and acceptor dyes, which reveals the conformational state of the H-DNA molecule. A 532-nm laser (CrystaLaser DPSS, 10 mW) was used to excite donor molecules and fluorescence intensities of both dyes were measured by an EMCCD (Andor iXon DV887, Andor technology). To observe the change of FRET efficiency in real time, we measured the time-lapse FRET traces with the repetition rate of 10 Hz. To study kinetic features of the conformational transition with dynamic disorder, we acquired the FRET time traces for a long period (>100 sec).

### Clustering at a higher dimension

For given *N* and *K*, total *N*(*N* − 1) intra-basin transition rates $k_{a,b}^{\left(\mu \right)}$ (*a*, *b* ∈ {1, 2, ⋯, *N*}, *a* ≠ *b*) are defined in the *μ*-th basin (or *μ*-th internal state) and total *K*(*K* − 1) inter-basin transitions are conceivable. To cluster the kinetic information of H-DNA data obtained from VB-DCMM, we consider the *kinetic arrow*, 2*N*(*N* − 1)-dimensional array of data, which has the structure of $C_{i}\equiv \left(\left\{\log k_{a,b}^{i,\text{bf}}\right\},\left\{\log k_{a,b}^{i,\text{af}}\right\}\right)$ where the subscript *i* denotes an index referring to one of *K*(*K* − 1) possible inter-basin transitions linking two internal states (*μ* ≠ *ν*). For a kinetic scheme made of a network of reversible transitions between *K* internal states, the transition between two internal states should be bidirectional; thus for a given inter-basin transition path *i*, there should be a kinetic path *j* antiparallel to the path *i*, satisfying $∥C_{i}-{\tilde{C}}_{j}∥\;\approx \;0$, where ${\tilde{C}}_{j}\equiv \left(\left\{\log k_{a,b}^{j,\text{af}}\right\},\left\{\log k_{a,b}^{j,\text{bf}}\right\}\right)$. In our problem, the set of all the data generated as an outcome of VB-DCMM can in principle be clustered into the disjoint subsets of size 2 partitioning the K transition paths, {K|1≤K≤K(K-1)}, and one realization of such disjoint subsets will minimize the pairwise sum of Euclidean distances $∥C_{\alpha }-{\tilde{C}}_{\beta }∥$ for all *α* and *β*; however, the method suffers from high computational cost as the possible number of clusters increases rapidly with *N* and *K*.

To alleviate the computational cost for large *N* and *K*, we modified the original method. We first searched the the best partitioning set of data $S^{*}\left(K\right)$ for a given K that minimizes the Euclidean distance between all the pairs of centroids,

$$D^{c}\left(K\right)=\frac{2}{K}\sum_{\left(i,j\right)}{\left(d_{ij}^{c}\right)}^{2}$$ (7)

where $d_{ij}^{c}=∥C_{i}^{c}-{\tilde{C}}_{j}^{c}∥$ with $C_{i}^{c}\equiv \left(\left\{\langle \log k_{a,b}^{i,\text{bf}}\rangle \right\},\left\{\langle \log k_{a,b}^{i,\text{af}}\rangle \right\}\right)$, ${\tilde{C}}_{j}^{c}\equiv \left(\left\{\langle \log k_{a,b}^{j,\text{af}}\rangle \right\},\left\{\langle \log k_{a,b}^{j,\text{bf}}\rangle \right\}\right)$, and 〈…〉 denotes the centroid of clustered data. To obtain the best clustering result for a given K, we conducted k-means clustering using *k*_*means* function from scikit-learn libraries [90] with 20,000 different random initial conditions in each analysis. It is expected that $D^{c}(K)=\frac{2}{K}\sum_{\left(i,j\right)}{\left(d_{ij}^{c}\right)}^{2}\geq \frac{2}{K}\sum_{S^{*}(K)}{\left(d_{ij}^{c}\right)}^{2}$. The summation, ∑_(*i*,*j*)_, signifies that the sum is taken over the disjoint subsets of size 2 partitioning a set {1,…,K} with K being an even number) and $S^{*}\left(K\right)$ is the best partitioning set that minimizes the value of $D^{c}\left(K\right)$ for a given K. For example, provided that there are 4 kinetic arrows made of centroids (*i* = 1, 2, 3, 4), which minimizes $D^{c}$ at K=2 when *i* = 1 is paired with *i* = 3 and *i* = 2 with *i* = 4, then S*(2) = {{1, 3}, {2, 4}} and $D^{c}\left(2\right)=d_{13}^{c}+d_{24}^{c}$.

Next, in order to decide the optimal K, we calculated pairing distance between paired clusters in $S^{*}\left(K\right)$ again, but this time using all the elements in each cluster. The total pairing score

$$D\left(K\right)\equiv \frac{2}{K}\sum_{S^{*}\left(K\right)}\left\langle d_{ij}\right\rangle ,$$ (8)

where the average pairing distance between two clusters *i* and *j* is defined as $\langle d_{ij}\rangle \equiv \frac{1}{M_{i}M_{j}}\sum_{n}^{M_{i}}\sum_{m}^{M_{j}}∥C_{i_{n}}-{\tilde{C}}_{j_{m}}∥$ where $C_{i_{n}}=\left(\left\{\log k_{a,b}^{i_{n},\text{bf}}\right\},\left\{\log k_{a,b}^{i_{n},\text{af}}\right\}\right)$, ${\tilde{C}}_{j_{m}}=\left(\left\{\log k_{a,b}^{j_{m},\text{af}}\right\},\left\{\log k_{a,b}^{j_{m},\text{bf}}\right\}\right)$, and *n* refers to an index for the element in the *i*-th cluster and *m* to an index for the elements in the *j*-th cluster. *M*_*i*_ is the total number of the elements in the *i*-th cluster. Finally, the optimal $K^{*}$, minimizing D(K), is selected, i.e., $K^{*}=\arg\min D(K)$, and the interpretation of data is conducted for the best partitioning set $S^{*}\left(K=K^{*}\right)$.

For H-DNA data at three different Na^+^ concentrations, the optimal $K^{*}$ are determined at $K^{*}=6$ for [Na^+^] = 100 mM (Fig 7A), $K^{*}=10$ for [Na^+^] = 50 mM (S17B Fig), $K^{*}=12$ for [Na^+^] = 26 mM (S18B Fig). This implies that the complexity of conformational space of H-DNA increases at low salt condition (also see the scatter plot of (*k*_*L* → *H*_, *k*_*H* → *L*_) in Figs 7A, S17A and S18A).

The clustering results presented in this study remain robust regardless of the choice of distance metric. K-means clustering using *L*1 distance (“city block”) measure with 20,000 different random initial conditions also was led to qualitatively similar results (S19 Fig). Furthermore, as an alternative clustering algorithm, we also tested “affinity propagation” [58] on our data, and the results remain qualitatively identical (see S20 Fig). In the affinity propagation method, negative square-euclidean distance was employed as a similarity metric (*s*(*i*, *j*) = −||***x***_*i*_ − ***x***_*j*_||^2^) where ***x***_*i*_ denotes the coordinate of the *i*-th data point. The objective of the algorithm is to optimize the factorized probability distribution which approximates the net similarity S, defined as $S∼\prod_{i=1}^{N}e^{s(i,c_{i})}$. Here, *c*_*i*_ is the index of the exemplar of *i*-th data point ***x***_*i*_. For example, if *c*_*i*_ = *k*, ***x***_*k*_ is an exemplar of ***x***_*i*_ and ***x***_*i*_ belongs to the cluster represented by ***x***_*k*_. Multiple iterations of message passing are carried out until convergence is achieved in the result and the best result of clustering is acquired. For implementation, we used *AffinityPropagation* class from *scikit* [90] library with varying “preference” as an input parameter, where the preference denotes the logarithm of probability that *i*-th data point *x*_*i*_ selects itself as an exemplar. Further details of the algorithm are available in Ref. [58].

## Supporting Information

S1 Text Supplementary Information. (PDF)

S1 FigVB-DCMM analysis on synthetic data generated with the following parameters: *K*^true^ = 2, *γ*^(1)→(2)^Δ*t* = γ^(2)→(1)^Δ*t* = 0.001, $k_{L\to H}^{\left(1\right)}\Delta t=k_{H\to L}^{\left(1\right)}\Delta t=0.05$, $k_{L\to H}^{\left(2\right)}\Delta t=k_{H\to L}^{\left(2\right)}\Delta t=0.0025$.(TIF)

S2 Fig Re-calculated accuracy of model prediction (〈*χ*〉) by assuming “*K* = 1”. (TIF)

S3 Fig Systematic validation of VB-DCMM on synthetic data generated under various conditions with *T*_*obs*_/Δ*t* = 8800. (TIF)

S4 Fig Accuracy of VB-DCMM on synthetic data with *T*_*obs*_/Δ*t* = 4400 and 2200. (TIF)

S5 Fig VB-DCMM analysis on synthetic data having three internal states. (TIF)

S6 Fig VB-DCMM analysis on synthetic data having 3 observables. (TIF)

S7 Fig VB-DCMM analysis on synthetic data having 4 observables (*o* = 1, 2, 3, and 4) when internal state *x* = 1, or having 2 (*o* = 1, 3) observables when *x* = 2. (TIF)

S8 Fig Average accuracy of the model prediction 〈*χ*〉 versus *D*_tot_ = *D*_conf_ + 0.8*D*_int_ from various synthetic data. (TIF)

S9 Fig Representative time traces of H-DNA dynamics and their analysis using VB-DCMM at [Na^+^] = 50 mM. (TIF)

S10 Fig Representative time traces of H-DNA dynamics and their analysis using VB-DCMM at [Na^+^] = 26 mM. (TIF)

S11 Fig Representative time traces of H-DNA dynamics which display more than two internal states within the trace at [Na^+^] = 26 mM. (TIF)

S12 FigVB-DCMM analysis on synthetic data generated with the following parameters: *T*_*obs*_/Δ*t* = 4400, *K*^true^ = 2, *γ*^(1)→(2)^Δ*t* = γ^(2)→(1)^Δ*t* = 0.001, $k_{L\to H}^{\left(1\right)}\Delta t=k_{H\to L}^{\left(1\right)}\Delta t=0.05$, $k_{L\to H}^{\left(2\right)}\Delta t=0.00625$, $k_{L\to H}^{\left(2\right)}\Delta t=0.0125$.(TIF)

S13 Fig Clustering synthetic data with three internal states (*K* = 3) with two observable states (*N* = 2). (TIF)

S14 Fig VB-DCMM analysis on synthetic data having four internal states (*K* = 4) with four observable states (*N* = 4). (TIF)

S15 Fig Clustering synthetic data with three internal states (*K* = 4) with four observable states (*N* = 4). (TIF)

S16 Fig Clustering H-DNA data ([Na^+^] = 100 mM). (TIF)

S17 Fig Clustering H-DNA data ([Na^+^] = 50 mM). (TIF)

S18 Fig Clustering H-DNA data ([Na^+^] = 26 mM). (TIF)

S19 Fig H-DNA data ([Na^+^] = 100 mM) analyzed with k-means clustering algorithm using “city block” distance (*L*1-distance). (TIF)

S20 Fig Clustering results of H-DNA data ([Na^+^] = 100 mM) from “affinity propagation” method. (TIF)

S21 Fig Accuracy of the model prediction in terms of 〈*χ*〉 under varying prior parameters *u*_*b*_, and *u*_*bd*_. (TIF)

S22 Fig Accuracy of the model prediction in terms of 〈*χ*〉 under varying prior parameters *u*_*a*_, and *u*_*ad*_. (TIF)

S23 Fig Effect of decomposing the original H-DNA time traces into its homogeneous Markov components. (TIF)

S24 Fig Comparison of *φ*_20_ = *σ*_20_/*μ*_20_ histograms on synthetic data before and after removing dynamic heterogeneity (red) by decomposing the original traces into the pieces according to estimated internal state trace. (TIF)

S25 Fig Comparison between VB-DCMM and sticky-iAMM. (TIF)
