# Supplementary material for: Decoding Single Molecule Time Traces with Dynamic Disorder
Source: PLoS Comput Biol. 2016 Dec 27;12(12):e1005286. doi: 10.1371/journal.pcbi.1005286 (PMC5226833; doi:10.1371/journal.pcbi.1005286)
Supplement: S1 Text — (PDF) [file pcbi.1005286.s001.pdf]

Supplementary Information: Decoding single molecule time traces with dynamic disorder

Wonseok Hwang,[1] Il-Buem Lee,[2] Seok-Cheol Hong,[1,2] and Changbong Hyeon[1,*]
[1]*Korea Institute for Advanced Study, Seoul 02455, Republic of Korea*
[2]*Department of Physics, Korea University, Seoul, 02841, Republic of Korea*
(Dated: December 15, 2016)

Contents

VB-DCMM algorithm: Backgrounds 1
Double Chain Markov Model 1
Maximum Evidence 2
Variational Bayes 2

VB-DCMM: Implementation 3
Derivations 3
Updating $q(\boldsymbol{\lambda})$ ($q(\boldsymbol{\pi})$, $q(\boldsymbol{A})$, and $q(\boldsymbol{B})$). 4
Updating $q(\boldsymbol{x})$. 6
Implementation. 8
Selection of prior parameters 8
Avoiding local minima 8
Computation time 8

Efficacy of VB-DCMM assessed by the law of large number 8

Other approaches 9
Markov Chain Monte Carlo (MCMC) technique 9
Comparison with Infinite Aggregated Markov Model (iAMM) 9

References 10

VB-DCMM ALGORITHM: BACKGROUNDS

We propose a new algorithm (Variational Bayes Double Chain Markov Model (VB-DCMM)) which combines three theoretical frameworks: Double Chain Markov Model (DCMM), maximum evidence, and Variational Bayes.

(1) DCMM consists of two layers of Markov chains. The elements of transition matrix in the Markov chain in the first layer are decided by the Markov chain in the second layer. In the light of analyzing single molecule time traces with dynamic disorder, the first and second layers of Markov chain are straightforwardly related to the transition dynamics along the sequences of hidden internal state ($\boldsymbol{x}$) and observable state ($\boldsymbol{o}$), respectively. While DCMM provides a straightforward conceptual framework to formulate the problem, the method itself, aiming to determine the best parameters for a given model, is not suitable for the best model selection (in our problem, the number of internal states, K).

(2) The maximum evidence enables a comparison between models, allowing us to select the best model; however, its computational cost is too high because the method requires considering the entire parameter space.

(3) To circumvent this difficulty, we incorporated the Variational Bayes technique into the algorithm and calculated an approximate value of the maximum evidence.

Double Chain Markov Model

Double Chain Markov Model (DCMM), first formally introduced by Berchtold [1], is defined with the following elements.

- T: The total length of data.
- K: The total number of internal states.
- N: The total number of observable states.
- $\boldsymbol{A}$: $(K \times K)$-transition matrix for $\boldsymbol{x}$.
- $\boldsymbol{B} = (\boldsymbol{B}^1, \boldsymbol{B}^2, ...\boldsymbol{B}^K)$ where $\boldsymbol{B}^\mu$ denotes $(N \times N)$-transition matrix for $\boldsymbol{o}$ when internal state $x(t) = \mu$.
- $\boldsymbol{x} = (x(1), x(2), \cdots, x(t), \cdots, x(T-1))$: The sequence of internal state. The transition, $x(t-1) \xrightarrow{\boldsymbol{A}} x(t)$, is modeled as a homogeneous Markov process, the rate of which is determined by the transition matrix $\boldsymbol{A}$. The value of the internal state at time t, $x(t) \in \{1, 2, \ldots, \mu, \ldots, K\}$, set the transition rate matrix $\boldsymbol{B}^{x(t)}$ which determines the transition of observable state from $o(t)$ to $o(t+1)$.
- $\boldsymbol{o} = (o(1), o(2), \cdots, o(t), \cdots, o(T))$: The sequence of observable state. The observable state denotes an index assigned to the value of data after filtering noises from experimental data,

such that $o(t) \in \{1, 2, \ldots, N\}$. The transition, $o(t) \xrightarrow{\boldsymbol{B}^{\mu}} o(t+1)$, is modeled as non-homogeneous Markov chain with a transition matrix $\boldsymbol{B}^{\mu}$, whose elements are decided by the internal state of x at time t ($x(t) = \mu$).

- $\boldsymbol{\pi} = (\pi_1, \pi_2, ..., \pi_K)$ where $\pi_\mu = P(x(1) = \mu|\boldsymbol{o}, \boldsymbol{A}, \boldsymbol{B})$ is the conditional probability of having $x(1) = \mu$ for a given $\boldsymbol{o}$, $\boldsymbol{A}$, and $\boldsymbol{B}$.

The probability of observing $\boldsymbol{o}$ and $\boldsymbol{x}$ with a given set of parameters $\boldsymbol{\lambda} = (\boldsymbol{\pi}, \boldsymbol{A}, \boldsymbol{B})$ can be written as

$$P(\boldsymbol{o}, \boldsymbol{x}|\boldsymbol{\lambda}) = \pi_{x(1)} B_{x(1),o(1),o(2)} \prod_{t=1}^{T-2} A_{x(t),x(t+1)} B_{x(t+1),o(t+1),o(t+2)}, \tag{S1}$$

where $A_{i,j} \equiv (\boldsymbol{A})_{ij}$ denotes the (i,j) element of the transition matrix $\boldsymbol{A}$, and $B_{\mu,i,j} \equiv (\boldsymbol{B}^{\mu})_{ij}$ denotes the (i, j) element of the transition matrix $\boldsymbol{B}^{\mu}$. By using Eq. S1 and adapting a similar procedure in Hidden Markov Model (HMM) (Forward-Backward algorithm and Baum-Welch algorithm), it is possible to determine the optimal parameter $\boldsymbol{\lambda}^*$ that (locally) maximizes $P(\boldsymbol{o}|\boldsymbol{\lambda})\,(= \sum_{\boldsymbol{x}} P(\boldsymbol{o}, \boldsymbol{x}|\boldsymbol{\lambda}))$ [1]. For given $\boldsymbol{o}$ and $\boldsymbol{\lambda}^*$, the sequence $\boldsymbol{x}$ for the internal state is determined (Viterbi algorithm) [1].

Maximum Evidence

In contrast to the maximum likelihood method used to identify optimal $\boldsymbol{\lambda}$ maximizing $P(\boldsymbol{o}|\boldsymbol{\lambda})$, the maximum evidence method selects the optimal model (in our case, optimal number of internal states K) maximizing $P(\boldsymbol{o}|\boldsymbol{K})$.

$$P(\boldsymbol{o}|\boldsymbol{K}) = \int P(\boldsymbol{o}|\boldsymbol{\lambda}')P(\boldsymbol{\lambda}'|\boldsymbol{K})d\boldsymbol{\lambda}'. \tag{S2}$$

In the maximum evidence, the likelihood value ($P(\boldsymbol{o}|\boldsymbol{\lambda})$) from an optimal $\boldsymbol{\lambda}$ is reduced by the factor $P(\boldsymbol{\lambda}|\boldsymbol{K})$, which could be smaller in more complex model since there are more freedom in choosing $\boldsymbol{\lambda}$. For $P(\boldsymbol{\lambda}'|\boldsymbol{K}) = \delta(\lambda - \lambda')$, the evidence becomes the likelihood.

Variational Bayes

The maximum evidence is formally suited for model selection, but the computational cost of the method, which requires integrating over the entire parameter space, is too large. To circumvent this difficulty, we combine the variational Bayes method with DCMM.

Let $q(\boldsymbol{Z})$ be an arbitrary probability distribution of a set of variable $\boldsymbol{Z}$ consisting of parameters and hidden variables of model (In DCMM, $\boldsymbol{Z} = (\boldsymbol{x}, \boldsymbol{\lambda})$). Then, from $\int q(\boldsymbol{Z})d\boldsymbol{Z} = 1$, the logarithm of the evidence, i.e., $\log P(\boldsymbol{o}|\boldsymbol{K})$ can be written as [2]

$$\begin{aligned}
\log(P(\boldsymbol{o}|\boldsymbol{K})) &= \int q(\boldsymbol{Z})\ \log(P(\boldsymbol{o}|\boldsymbol{K}))d\boldsymbol{Z} \\
&= \int q(\boldsymbol{Z})\ \log\left(P(\boldsymbol{o}|\boldsymbol{K})\frac{P(\boldsymbol{o}, \boldsymbol{Z}|\mathbf{K})}{q(\boldsymbol{Z})}\frac{q(\boldsymbol{Z})}{P(\boldsymbol{o}, \boldsymbol{Z}|\mathbf{K})}\right)d\boldsymbol{Z} \\
&= \int q(\boldsymbol{Z})\ \log\left(\frac{P(\boldsymbol{o}, \boldsymbol{Z}|\boldsymbol{K})}{q(\boldsymbol{Z})}\right)d\boldsymbol{Z} + \int q(\boldsymbol{Z})\ \log\left(\frac{q(\boldsymbol{Z})}{P(\boldsymbol{Z}|\mathbf{o}, \mathbf{K})}\right)d\boldsymbol{Z} \\
&= F[q] + D_{KL}(q||p)
\end{aligned} \tag{S3}$$

where p denotes $P(\boldsymbol{Z}|\boldsymbol{o}, \boldsymbol{K})$,

$$P(\boldsymbol{Z}|\boldsymbol{o}, \boldsymbol{K}) = P(\boldsymbol{o}, \boldsymbol{Z}|\boldsymbol{K})/P(\boldsymbol{o}|\boldsymbol{K}), \tag{S4}$$

$$F[q] \equiv \int q(\boldsymbol{Z})\ \log\left(\frac{P(\boldsymbol{o}, \boldsymbol{Z}|\boldsymbol{K})}{q(\boldsymbol{Z})}\right)d\boldsymbol{Z}, \tag{S5}$$

and

$$D_{KL}(q||p) \equiv \int q(\boldsymbol{Z}) \, \log \left(\frac{q(\boldsymbol{Z})}{P(\boldsymbol{Z}|\mathbf{o}, \mathbf{K})} \right) d\boldsymbol{Z}. \tag{S6}$$

As Kullback-Leibler divergence always satisfies $D_{KL}(q||p) \geq 0$, the following inequality holds.

$$\log(P(\boldsymbol{o}|\boldsymbol{K})) = F[q] + D_{KL}(q||p) \geq F[q] \tag{S7}$$

The Variational Bayes method aims to maximize the lower bound of $F[q]$ (and thus the lower bound of $\log(P(\boldsymbol{o}|\boldsymbol{K}))$ by refining $q(\boldsymbol{Z})$ iteratively, anticipating that $F[q]$ converges to $\log(P(\boldsymbol{o}|\boldsymbol{K}))$. When $F[q]$ converges to $\log(P(\boldsymbol{o}|\boldsymbol{K}))$, $D_{KL}(q||p)$ converges to 0, indicating that $q(\boldsymbol{Z})$ converges to $P(\boldsymbol{Z}|\mathbf{o}, \mathbf{K})$. Thus the variational method simultaneously find the approximate values of the evidence and $P(\boldsymbol{Z}|\mathbf{o}, \mathbf{K})$, the probability distribution of model parameters and hidden variables of each model for given data. In the light of DCMM, $P(\boldsymbol{o}, \boldsymbol{Z}|\boldsymbol{K})$ is written as

$$\begin{aligned} P(\boldsymbol{o}, \boldsymbol{Z}|\boldsymbol{K}) &= P(\boldsymbol{o}, \boldsymbol{x}, \boldsymbol{\pi}, \boldsymbol{A}, \boldsymbol{B}|\boldsymbol{K}) \\ &= P(\boldsymbol{o}, \boldsymbol{x}|\boldsymbol{\pi}, \boldsymbol{A}, \boldsymbol{B}) P(\boldsymbol{\pi}, \boldsymbol{A}, \boldsymbol{B}|\boldsymbol{K}) \\ &= P(\boldsymbol{o}, \boldsymbol{x}|\boldsymbol{\pi}, \boldsymbol{A}, \boldsymbol{B}) P(\boldsymbol{\pi}|\boldsymbol{K}) P(\boldsymbol{A}|\boldsymbol{K}) P(\boldsymbol{B}|\boldsymbol{K}) \end{aligned} \tag{S8}$$

Dirichlet distributions are used for prior distributions $P(\boldsymbol{\pi}|\boldsymbol{K})$, $P(\boldsymbol{A}|\boldsymbol{K})$, and $P(\boldsymbol{B}|\boldsymbol{K})$ to render $q(\boldsymbol{Z})$ into the same type of function (Dirichlet distribution) as well [3].

$$\begin{aligned} P(\boldsymbol{\pi}|\boldsymbol{K}) &= Dir(\pi_1, \pi_2, ..., \pi_K | u_1^\pi, u_2^\pi, ..., u_K^\pi) \\ &= \frac{\Gamma(u_0^\pi)}{\prod_{\mu=1}^K \Gamma(u_\mu^\pi)} \prod_{\mu=1}^K \pi_\mu^{u_\mu^\pi - 1} \end{aligned} \tag{S9}$$

where u_μ^π ($\mu \geq 1$) refers to a parameter of Dirichlet distribution with $u_0^\pi = \sum_{\mu=1}^K u_\mu^\pi$, $\sum_{\mu=1}^K \pi_\mu = 1$, and $\Gamma(\cdot)$ denotes the gamma function. The superscript π in u_μ^π implies that u_μ^π is the parameter involving the probability π.

$$\begin{aligned} P(\boldsymbol{A}|\boldsymbol{K}) &= \prod_{\mu=1}^K Dir(A_{\mu,1}, A_{\mu,2}, ..., A_{\mu,K} | u_{\mu,1}^A, u_{\mu,2}^A, ..., u_{\mu,K}^A) \\ &= \prod_{\mu=1}^K \frac{\Gamma(u_{\mu,0}^A)}{\prod_{\nu=1}^K \Gamma(u_{\mu,\nu}^A)} \prod_{\nu=1}^K A_{\mu,\nu}^{u_{\mu,\nu}^A - 1} \end{aligned} \tag{S10}$$

where $u_{\mu,0}^A = \sum_{\nu=1}^K u_{\mu,\nu}^A$, $\sum_{\nu=1}^K A_{\mu,\nu} = 1$, and $u_{\mu,\nu}^A$ ($\nu \geq 1$) again refers to a parameter of Dirichlet distribution with the superscript A implying that the parameter is involved with the transition matrix $\boldsymbol{A}$.

$$\begin{aligned} P(\boldsymbol{B}|\boldsymbol{K}) &= \prod_{\mu=1}^K \prod_{i=1}^N Dir(B_{\mu,i,1}, B_{\mu,i,2}, ..., B_{\mu,i,N} | u_{\mu,i,1}^B, u_{\mu,i,2}^B, ..., u_{\mu,i,N}^B) \\ &= \prod_{\mu=1}^K \prod_{i=1}^N \frac{\Gamma(u_{\mu,i,0}^B)}{\prod_{j=1}^N \Gamma(u_{\mu,i,j}^B)} \prod_{j=1}^N B_{\mu,i,j}^{u_{\mu,i,j}^B - 1} \end{aligned} \tag{S11}$$

where $u_{\mu,i,0}^B = \sum_{j=1}^N u_{\mu,i,j}^B$, $\sum_{j=1}^N B_{\mu,i,j} = 1$, and $u_{\mu,i,j}^B$ ($j \geq 1$) again stands for a parameter of Dirichlet distribution involving the transition matrix $\boldsymbol{B}^\mu$.

VB-DCMM: IMPLEMENTATION

Derivations

A factorized form of $q(\boldsymbol{Z}) = q(\boldsymbol{\pi}) q(\boldsymbol{A}) q(\boldsymbol{B}) q(\boldsymbol{x})$ was assumed to find an approximate $q(\boldsymbol{Z})$. As a result, $F[q]$ (Eq.S5) can be expanded in term by term as

$$
\begin{aligned}
F[q] &= \int q(\boldsymbol{Z})\ \log\left(\frac{P(\boldsymbol{o},\boldsymbol{Z}|\boldsymbol{K})}{q(\boldsymbol{Z})}\right) d\boldsymbol{Z} \\
&= \int q(\boldsymbol{\lambda})q(\boldsymbol{x})\Big(\log\frac{P(\boldsymbol{\pi}|\boldsymbol{K})}{q(\boldsymbol{\pi})} + \log\frac{P(\boldsymbol{A}|\boldsymbol{K})}{q(\boldsymbol{A})} + \log\frac{P(\boldsymbol{B}|\boldsymbol{K})}{q(\boldsymbol{B})} + \log\frac{P(\boldsymbol{o},\boldsymbol{x}|\boldsymbol{\pi},\boldsymbol{A},\boldsymbol{B})}{q(\boldsymbol{x})}\Big) d\boldsymbol{\lambda} d\boldsymbol{x}
\end{aligned} \tag{S12}
$$

By substituting Eq.(S1) to Eq.(S12) we obtain

$$
F[q] = F[q(\boldsymbol{\pi})] + F[q(\boldsymbol{A})] + F[q(\boldsymbol{B})] + F[q(\boldsymbol{x})] \tag{S13}
$$

where

$$
\begin{aligned}
F[q(\boldsymbol{\pi})] &= \int q(\boldsymbol{\lambda})q(\boldsymbol{x})\Big(\log\frac{P(\boldsymbol{\pi}|\boldsymbol{K})}{q(\boldsymbol{\pi})} + \log(\pi_{x(1)})\Big) d\boldsymbol{\lambda} d\boldsymbol{x}, \\
F[q(\boldsymbol{A})] &= \int q(\boldsymbol{\lambda})q(\boldsymbol{x})\Big(\log\frac{P(\boldsymbol{A}|\boldsymbol{K})}{q(\boldsymbol{A})} + \sum_{t=1}^{T-2}\log(A_{x(t),x(t+1)})\Big) d\boldsymbol{\lambda} d\boldsymbol{x}, \\
F[q(\boldsymbol{B})] &= \int q(\boldsymbol{\lambda})q(\boldsymbol{x})\Big(\log\frac{P(\boldsymbol{B}|\boldsymbol{K})}{q(\boldsymbol{B})} + \sum_{t=1}^{T-1}\log(B_{x(t),o(t),o(t+1)})\Big) d\boldsymbol{\lambda} d\boldsymbol{x}, \\
F[q(\boldsymbol{x})] &= -\int q(\boldsymbol{x})\log(q(\boldsymbol{x})) d\boldsymbol{x}.
\end{aligned}
$$

Updating $q(\boldsymbol{\lambda})$ ($q(\boldsymbol{\pi})$, $q(\boldsymbol{A})$, and $q(\boldsymbol{B})$).

We first set $q(\boldsymbol{x}) = P(\boldsymbol{x}|\boldsymbol{o},\boldsymbol{\lambda}')$ with given initial values of $\boldsymbol{\lambda}' = (\boldsymbol{\pi}', \boldsymbol{A}', \text{and } \boldsymbol{B}')$. Substitution of Eqs.(S9),(S10),(S11) to $F[q]$ (Eq.(S13)) and integration over $\boldsymbol{x}$ lead to

$$
\begin{aligned}
F[q(\boldsymbol{\pi})] &= \int q(\boldsymbol{\pi})q(\boldsymbol{A})q(\boldsymbol{B})q(\boldsymbol{x})\Big(\log\frac{P(\boldsymbol{\pi}|\boldsymbol{K})}{q(\boldsymbol{\pi})} + \log(\pi_{x(1)})\Big) d\boldsymbol{\pi} d\boldsymbol{A} d\boldsymbol{B} d\boldsymbol{x} \\
&= \int q(\boldsymbol{\pi})q(\boldsymbol{x})\Big(\log\frac{P(\boldsymbol{\pi}|\boldsymbol{K})}{q(\boldsymbol{\pi})} + \log(\pi_{x(1)})\Big) d\boldsymbol{\pi} d\boldsymbol{x} \\
&= \int q(\boldsymbol{\pi})\Big(\log\frac{\prod_{\mu=1}^{K}\pi_\mu^{u_\mu^\pi-1}}{q(\boldsymbol{\pi})} + \sum_{\mu=1}^{K} P(x(1)=\mu|\boldsymbol{o},\boldsymbol{\lambda}')\log(\pi_\mu)\Big) d\boldsymbol{\pi} + const. \\
&= \int q(\boldsymbol{\pi})\Big(\log\frac{\prod_{\mu=1}^{K}\pi_\mu^{u_\mu^\pi-1}}{q(\boldsymbol{\pi})} + \sum_{\mu=1}^{K}\log(\pi_\mu^{P(x(1)=\mu|\boldsymbol{o},\boldsymbol{\lambda}')})\Big) d\boldsymbol{\pi} + const. \\
&= \int q(\boldsymbol{\pi})\Big(\log\frac{\prod_{\mu=1}^{K}\pi_\mu^{u_\mu^\pi+P(x(1)=\mu|\boldsymbol{o},\boldsymbol{\lambda}')-1}}{q(\boldsymbol{\pi})}\Big) d\boldsymbol{\pi} + const.
\end{aligned} \tag{S14}
$$

To derive the equations above, we first use $\int q(\boldsymbol{A})d\boldsymbol{A} = \int q(\boldsymbol{B})d\boldsymbol{B} = 1$, and then replace the $\int q(\boldsymbol{x})\log\pi_{x(1)}d\boldsymbol{x}$ with $\sum_{x(1),x(2),...,x(T-1)} P(x(1),x(2),...,x(T-1)|\boldsymbol{o},\boldsymbol{\lambda}')\log\pi_{x(1)}$ in the second to the third line. The normalization factor of $P(\boldsymbol{\pi}|\boldsymbol{K})$ (Eq.(S9)) is added as a constant term. Finally, by changing the sum of log to multiplication of its arguments and combine all the integrands together, the final result is obtained. By a similar procedure, $F[q(\boldsymbol{A})]$ and $F[q(\boldsymbol{B})]$ can be written as

$$
\begin{aligned}
F[q(\boldsymbol{A})] &= \int q(\boldsymbol{\pi})q(\boldsymbol{A})q(\boldsymbol{B})q(\boldsymbol{x}) \log \frac{P(\boldsymbol{A}|\boldsymbol{K})}{q(\boldsymbol{A})} + \sum_{t=1}^{T-2} \log(A_{x(t),x(t+1)})\Big) d\boldsymbol{\pi} d\boldsymbol{A} d\boldsymbol{B} d\boldsymbol{x} \\
&= \int q(\boldsymbol{A}) \Big(\log \frac{\prod_{\mu,\nu=1}^{K} A_{\mu,\nu}^{u^A_{\mu,\nu}-1}}{q(\boldsymbol{A})} + \sum_{\mu,\nu=1}^{K} \sum_{t=1}^{T-2} P(x(t)=\mu, x(t+1)=\nu|\boldsymbol{o},\boldsymbol{\lambda}')\ \log(A_{\mu,\nu}) \Big) d\boldsymbol{A} + const. \\
&= \int q(\boldsymbol{A}) \Big(\log \frac{\prod_{\mu,\nu=1}^{K} A_{\mu,\nu}^{u^A_{\mu,\nu}+\sum_{t=1}^{T-2} P(x(t)=\mu,x(t+1)=\nu|\boldsymbol{o},\boldsymbol{\lambda}')-1}}{q(\boldsymbol{A})} \Big) d\boldsymbol{A} + const.
\end{aligned} \tag{S15}
$$

$$
\begin{aligned}
F[q(\boldsymbol{B})] &= \int q(\boldsymbol{\pi})q(\boldsymbol{A})q(\boldsymbol{B})q(\boldsymbol{x}) \log \frac{P(\boldsymbol{B}|\boldsymbol{K})}{q(\boldsymbol{B})} + \sum_{t=1}^{T-1} \log(B_{x(t),o(t),o(t+1)})\Big) d\boldsymbol{\pi} d\boldsymbol{A} d\boldsymbol{B} d\boldsymbol{x} \\
&= \int q(\boldsymbol{B}) \Big(\log \frac{\prod_{\mu=1}^{K}\prod_{i,j=1}^{N} B_{\mu,i,j}^{u^B_{\mu,i,j}-1}}{q(\boldsymbol{B})} + \sum_{\mu=1}^{K} \sum_{t=1}^{T-1} P(x(t)=\mu|\boldsymbol{o},\boldsymbol{\lambda}')\ \log(B_{\mu,o(t),o(t+1)}) \Big) d\boldsymbol{B} + const. \\
&= \int q(\boldsymbol{B}) \Big(\log \frac{\prod_{\mu=1}^{K}\prod_{i,j=1}^{N} B_{\mu,i,j}^{u^B_{\mu,i,j}-1}}{q(\boldsymbol{B})} + \sum_{\mu=1}^{K} \sum_{i,j=1}^{N} \sum_{\substack{t=1 \\ o(t)=i,o(t+1)=j}}^{T-1} P(x(t)=\mu|\boldsymbol{o},\boldsymbol{\lambda}')\ \log(B_{\mu,o(t),o(t+1)}) \Big) d\boldsymbol{B} + const. \\
&= \int q(\boldsymbol{B}) \Big(\log \frac{\prod_{\mu=1}^{K}\prod_{i,j=1}^{N} B_{\mu,i,j}^{u^B_{\mu,i,j}+\sum_{t=1,o(t)=i,o(t+1)=j}^{T-1} P(x(t)=\mu|\boldsymbol{o},\boldsymbol{\lambda}')-1}}{q(\boldsymbol{B})} \Big) d\boldsymbol{B} + const.
\end{aligned} \tag{S16}
$$

After combining the above three equations together we get

$$
\begin{aligned}
F[q] &= \int q(\boldsymbol{\pi}) \log \Big(\frac{\prod_{\mu=1}^{K} \pi_{\mu}^{W^{\pi}_{\mu}-1}}{q(\boldsymbol{\pi})} \Big) d\boldsymbol{\pi} \\
&\quad + \int q(\boldsymbol{A}) \log \Big(\frac{\prod_{\mu=1,\nu=1}^{K} A_{\mu,\nu}^{W^A_{\mu,l}-1}}{q(\boldsymbol{A})} \Big) d\boldsymbol{A} \\
&\quad + \int q(\boldsymbol{B}) \log \Big(\frac{\prod_{\mu=1}^{K}\prod_{i=1,j=1}^{N} (B_{\mu,i,j})^{W^B_{\mu,i,j}-1}}{q(\boldsymbol{B})}` \Big) d\boldsymbol{B} \\
&\quad + const. \\
&= - D_{KL}(q(\boldsymbol{\pi})||Dir(\pi_1,\pi_2,...,\pi_K|W^{\pi}_1, W^{\pi}_2, ..., W^{\pi}_K)) \\
&\quad - D_{KL}(q(\boldsymbol{A})|| \prod_{\mu=1}^{K} Dir(A_{\mu,1}, A_{\mu,2}, ..., A_{\mu,K}|W^A_{\mu,1}, W^A_{\mu,2}, ..., W^A_{\mu,K})) \\
&\quad - D_{KL}(q(\boldsymbol{B})|| \prod_{\mu=1}^{K}\prod_{i=1}^{N} Dir(B_{\mu,i,1}, B_{\mu,i,2}, ..., B_{\mu,i,L}|W^B_{\mu,i,1}, W^B_{\mu,i,2}, ..., W^B_{\mu,i,L})) \\
&\quad + const.
\end{aligned} \tag{S17}
$$

where

$$
\begin{aligned}
W^{\pi}_{\mu} &= u^{\pi}_{\mu} + P(x(1)=\mu|\boldsymbol{o},\boldsymbol{\lambda}'), \\
W^A_{\mu,\nu} &= u^A_{\mu,\nu} + \sum_{t=1}^{T-2} P(x(t)=\mu, x(t+1)=\nu|\boldsymbol{o},\boldsymbol{\lambda}'), \\
W^B_{\mu.i,j} &= u^B_{\mu,i,j} + \sum_{\substack{t=1 \\ o(t)=i,o(t+1)=j}}^{T-1} P(x(t)=\mu|\boldsymbol{o},\boldsymbol{\lambda}').
\end{aligned}
$$

Now by setting $q(\boldsymbol{\pi}), q(\boldsymbol{A})$ and $q(\boldsymbol{B})$ equal to Dirichlet distributions with new parameter W, we can increase $F[q]$ as $-D_{KL}(\cdot) \leq 0$. $P(x(1)=\mu|\boldsymbol{o},\boldsymbol{\lambda}')$ and $P(x(t)=\mu, x(t+1)=\nu|\boldsymbol{o},\boldsymbol{\lambda}')$ can be calculated efficiently by using Forward-Backward algorithm [1].

Updating $q(\boldsymbol{x})$.

Now we integrate $F[q]$ over $\boldsymbol{\pi}$, $\boldsymbol{A}$, and $\boldsymbol{B}$ with fixed (and updated) $q(\boldsymbol{\pi})$, $q(\boldsymbol{A})$, and $q(\boldsymbol{B})$ to optimize $q(\boldsymbol{x})$. From Eq.(S12), $F[q(\boldsymbol{\pi})]$ can be written as

$$
\begin{aligned}
F[q(\boldsymbol{\pi})] &= \int q(\boldsymbol{\pi})q(\boldsymbol{A})q(\boldsymbol{B})q(\boldsymbol{x})\Big(\log\frac{P(\boldsymbol{\pi}|\boldsymbol{K})}{q(\boldsymbol{\pi})} + \log(\pi_{x(1)})\Big) d\boldsymbol{\pi} d\boldsymbol{A} d\boldsymbol{B} d\boldsymbol{x} \\
&= \int q(\boldsymbol{\pi})q(\boldsymbol{x})\Big(\log\frac{P(\boldsymbol{\pi}|\boldsymbol{K})}{q(\boldsymbol{\pi})} + \log(\pi_{x(1)})\Big) d\boldsymbol{\pi} d\boldsymbol{x} \\
&= \int q(\boldsymbol{\pi})q(\boldsymbol{x})\Big(\log(\pi_{x(1)})\Big) d\boldsymbol{\pi} d\boldsymbol{x} + const.
\end{aligned}
\tag{S18}
$$

We first use $\int q(\boldsymbol{A})d\boldsymbol{A} = \int q(\boldsymbol{B})d\boldsymbol{B} = 1$ as the integrand does not depend on $\boldsymbol{A}$ and $\boldsymbol{B}$. As $\log\frac{P(\boldsymbol{\pi}|\boldsymbol{K})}{q(\boldsymbol{\pi})}$ does not depend on $\boldsymbol{x}$, the result of integration of this term can be written as a constant (*const.*). By similar procedure, $F[q(\boldsymbol{A})]$ and $F[q(\boldsymbol{B})]$ are written as

$$
\begin{aligned}
F[q(\boldsymbol{A})] &= \int\int q(\boldsymbol{\pi})q(\boldsymbol{A})q(\boldsymbol{B})q(\boldsymbol{x})\Big(\log\frac{P(\boldsymbol{A}|\boldsymbol{K})}{q(\boldsymbol{A})} + \sum_{t=1}^{T-2}\log(A_{x(t),x(t+1)})\Big) d\boldsymbol{\pi} d\boldsymbol{A} d\boldsymbol{B} d\boldsymbol{x} \\
&= \int q(\boldsymbol{A})q(\boldsymbol{x})\Big(\sum_{t=1}^{T-2}\log(A_{x(t),x(t+1)})\Big) d\boldsymbol{A} d\boldsymbol{x} + const.
\end{aligned}
\tag{S19}
$$

$$
\begin{aligned}
F[q(\boldsymbol{B})] &= \int q(\boldsymbol{\pi})q(\boldsymbol{A})q(\boldsymbol{B})q(\boldsymbol{x})\Big(\log\frac{P(\boldsymbol{B}|\boldsymbol{K})}{q(\boldsymbol{B})} + \sum_{t=1}^{T-1}\log(B_{x(t),o(t),o(t+1)})\Big) d\boldsymbol{\pi} d\boldsymbol{A} d\boldsymbol{B} d\boldsymbol{x} \\
&= \int q(\boldsymbol{B})q(\boldsymbol{x})\Big(\sum_{t=1}^{T-1}\log(B_{x(t),o(t),o(t+1)})\Big) d\boldsymbol{B} d\boldsymbol{x} + const.
\end{aligned}
\tag{S20}
$$

By combining Eq.(S18-S20), we get

$$
\begin{aligned}
F[q] =& \int q(\boldsymbol{x})\Big(\int q(\boldsymbol{\pi})\log(\pi_{x(1)})d\boldsymbol{\pi} + \int q(\boldsymbol{A})\sum_{t=1}^{T-2}\log(A_{x(t),x(t+1)})d\boldsymbol{A} \\
&+ \int q(\boldsymbol{B})\sum_{t=1}^{T-1}\log(B_{x(t),o_t,o(t+1)})d\boldsymbol{B} - \log(q(\boldsymbol{x}))\Big) d\boldsymbol{x} \\
&+ const. \\
=& \int q(\boldsymbol{x})\log\left(\frac{\pi''_{x(1)}B''_{x(1),o(1),o(2)}\prod_{t=1}^{T-2}A''_{x(t),x(t+1)}B''_{x(t+1),o(t+1),o(t+2)}}{q(\boldsymbol{x})}\right) d\boldsymbol{x} + const.
\end{aligned}
\tag{S21}
$$

where

$$\log \pi''_{x(1)} = \int q(\boldsymbol{\pi}) \log(\pi_{x(1)}) d\boldsymbol{\pi} = \psi(W^{\pi}_{x(1)}) - \psi(\sum_{k=1}^{K} W^{\pi}_{k}),$$

$$\log A''_{x(t),x(t+1)} = \int q(\boldsymbol{A}) \log(A_{x(t),x(t+1)}) d\boldsymbol{A} = \psi(W^{A}_{x(t),x(t+1)}) - \psi(\sum_{k=1}^{K} W^{A}_{x(t),k}),$$

$$\log B''_{x(t),o(t),o(t+1)} = \int q(\boldsymbol{B}) \log(B_{x(t),o(t),o(t+1)}) d\boldsymbol{B} = \psi(W^{B}_{x(t),o(t),o(t+1)}) - \psi(\sum_{j=1}^{N} W^{B}_{x(t),o(t),j}).$$

Here, $\psi(\cdot)$ denotes the digamma function ($\psi(x) = \frac{d}{dx}\log\Gamma(x)$). Now that $F[q]$ again has a form of $-D_{KL}(\cdot) + const.$, $F[q]$ can be maximized by minimizing the $D_{KL}(\cdot)$ term, which is achieved by setting

$$q''(\boldsymbol{x}) = \frac{\pi''_{x(1)} B''_{x(1),o(1),o(2)} \prod_{t=1}^{T-2} A''_{x(t),x(t+1)} B''_{x(t+1),o(t+1),o(t+2)}}{P(\boldsymbol{o}|\boldsymbol{\pi}'', \boldsymbol{A}'', \boldsymbol{B}'')} \tag{S22}$$

Note that, the numerator of the equation above is equal to $P(\boldsymbol{o}, \boldsymbol{x}|\boldsymbol{\pi}'', \boldsymbol{A}'', \boldsymbol{B}'')$ implying $q''(\boldsymbol{x}) = P(\boldsymbol{x}|\boldsymbol{o}, \boldsymbol{\pi}'', \boldsymbol{A}'', \boldsymbol{B}'')$.

With $q(\boldsymbol{x})$ and by replacing $\boldsymbol{\lambda}' = (\boldsymbol{\pi}', \boldsymbol{A}', \boldsymbol{B}')$ with $\boldsymbol{\lambda}'' = (\boldsymbol{\pi}'', \boldsymbol{A}'', \boldsymbol{B}'')$, one can further update $q(\boldsymbol{\pi}), q(\boldsymbol{A})$, and $q(\boldsymbol{B})$. These procedures are iterated until the value of $F[q]$ converges to a desired precision.

Finally, the converged $F[q]$ can be calculated by substituting the converged argument $q = q^*$ and parameters $\boldsymbol{\pi}^*, \boldsymbol{A}^*, \boldsymbol{B}^*$ into Eq.(S12).

$$\begin{aligned} F[q^*] = &- D_{KL}(Dir(W^{\boldsymbol{\pi}*})||Dir(u^{\boldsymbol{\pi}})) \\ &- D_{KL}(Dir(W^{\boldsymbol{A}*})||Dir(u^{\boldsymbol{A}})) \\ &- D_{KL}(Dir(W^{\boldsymbol{B}*})||Dir(u^{\boldsymbol{B}})) \\ &+ \log\ P(\boldsymbol{o}|\boldsymbol{\pi^*}, \boldsymbol{A^*}, \boldsymbol{B^*}) \\ &+ \log K! \end{aligned} \tag{S23}$$

The first three terms, $-D_{KL}(\cdot)$, correspond to penalties against the model complexity. The fourth term corresponds to the likelihood, which generally increases with K. The final $\log K!$ term is added to account for the symmetry of model [2]. K is the number of possible internal states in the model. Degeneracy arises from the freedom of permutating the labels. For example, if two internal states $x = 1, 2$ are found from VB-DCMM, a new model with $x = 2, 4$ and $\boldsymbol{B}^{x_{new}=2}$ ($= \boldsymbol{B}^{x=1}$), $\boldsymbol{B}^{x_{new}=4}$ ($= \boldsymbol{B}^{x=2}$) can also be a possible solution with an equal probability. Thus, overall evidence should be calculated with the sum of all possible cases that can be obtained from the permutation of labels for internal states. Thus a corrected evidence should be multiplied by $K!$, which results in introducing the additional factor $\log K!$ to $\log evidence$. In the analysis of real single molecule data, the number of observed internal states K_{obs} is not generally identical to the parameter K. In this case, the actual number of degenercy in labeling internal states should be ${}_K C_{K_{obs}} \times K_{obs}!$ instead of $K!$. To take this effect into account in calculating evidence function, we modified the original evidence function into the following form:

$$G(K) \equiv F(K) - \log{(K - K_{obs})!}. \tag{S24}$$

According to Eq. (S3), the increase of lower bound of $F[q]$ accompanies the decrease of $D_{KL}(q||p)$. Thus, it is expected that after multiple iterations, $F[q]$ (or $G[q]$) converges to $F[q^*]$ which satisfies $F[q] < F[q^*] \simeq \log P(\boldsymbol{o}|\boldsymbol{K})$). This implies that $D_{KL}(q^*||p) \simeq 0$. From

$$P(\boldsymbol{Z}|\boldsymbol{o}, \boldsymbol{K}) = P(\boldsymbol{\pi}|\boldsymbol{K})P(\boldsymbol{A}|\boldsymbol{K})P(\boldsymbol{B}|\boldsymbol{K})P(\boldsymbol{x}|\boldsymbol{o}, \boldsymbol{\pi}, \boldsymbol{A}, \boldsymbol{B}),$$

and $q(\boldsymbol{Z}) = q(\boldsymbol{x})q(\boldsymbol{\pi})q(\boldsymbol{A})q(\boldsymbol{B})$, it follows that

$$\begin{aligned} D_{KL}(q^*||p) = &D_{KL}(q^*(\boldsymbol{\pi})||P(\boldsymbol{\pi}|\boldsymbol{K})) \\ &+ D_{KL}(q^*(\boldsymbol{A})||P(\boldsymbol{A}|\boldsymbol{K})) \\ &+ D_{KL}(q^*(\boldsymbol{B})||P(\boldsymbol{B}|\boldsymbol{K})) \\ &+ D_{KL}(q^*(\boldsymbol{x})||P(\boldsymbol{x}|\boldsymbol{o}, \boldsymbol{\pi}, \boldsymbol{A}, \boldsymbol{B})). \end{aligned} \tag{S25}$$

Thus, $D_{KL}(q^*||p) \simeq 0$ implies $q^*(\boldsymbol{\pi}) \simeq P(\boldsymbol{\pi}|\boldsymbol{K})$, $q^*(\boldsymbol{A}) \simeq P(\boldsymbol{A}|\boldsymbol{K})$, $q^*(\boldsymbol{B}) \simeq P(\boldsymbol{B}|\boldsymbol{K})$, and

$$D_{KL}(q^*(\boldsymbol{x})||P(\boldsymbol{x}|\boldsymbol{o},\boldsymbol{\pi},\boldsymbol{A},\boldsymbol{B})) = \int d\boldsymbol{x}d\boldsymbol{\pi}d\boldsymbol{A}d\boldsymbol{B}\ q^*(\boldsymbol{x})\log\left(\frac{q^*(\boldsymbol{x})}{P(\boldsymbol{x}|\boldsymbol{o},\boldsymbol{\pi},\boldsymbol{A},\boldsymbol{B})}\right) \simeq 0. \tag{S26}$$

Eq. S26 also implies that, $q^*(\boldsymbol{x}) = P(\boldsymbol{x}|\boldsymbol{o},\boldsymbol{\pi}^*\boldsymbol{A}^*\boldsymbol{B}^*) \simeq P(\boldsymbol{x}|\boldsymbol{o},\boldsymbol{\pi},\boldsymbol{A},\boldsymbol{B})$. Finally, $\boldsymbol{\pi}^*,\boldsymbol{A}^*,\boldsymbol{B}^*$, which provide us with a set of rate constants (e.g. $\{k^{(\mu)}_{a\to b}\}$, $\{\gamma^{(\mu)\to(\nu)}\}$), are interpreted as the estimated model parameters.

Implementation.

Selection of prior parameters

The likelihood, $\log P(\boldsymbol{o}|\boldsymbol{\pi^*},\boldsymbol{A^*},\boldsymbol{B^*})$ in Eq.(S23) generally increases with K. Other terms, $-D_{KL}(\cdot)$, are always negative, which imposes a penalty against the model with a higher K. As the difference between two Dirichlet distributions vanishes when the posterior value W is equal to the prior parameter u and is minimized when the ratios between the element of W and that of u are identical (for example when $W^A_{i,j}/W^A_{i,k} = u^A_{i,j}/u^A_{i,k}$), Eq.(S23) provides a natural guideline for selecting the prior parameters. We have selected the prior parameters using the following rule.

- $u^{\pi}_{\mu} = 1$
- $u^A_{\mu,\nu} = 1$ for $\mu \neq \nu$
- $u^A_{\mu,\nu} =$ (transition rate (with $\Delta t = 1$) using a visual estimation)$^{-1}$.
- Perform Hidden Markov Analysis assuming $K = 1$ to construct a transition matrix $\boldsymbol{B}^h$ of homogeneous Markov process.
- Set $u^B_{\mu,i,j} = B^h_{i,j}/\min(\{B^h_{i,1}, B^h_{i,2}, ..., B^h_{i,N}\})$ for all μ.

For example, when roughly one internal-state transition is observed in the trace with $T_{obs}/\Delta t$= 2000, we set $u^A_{\mu,\nu} = 1/0.001 = 1000$. If $\boldsymbol{B}^h = \begin{pmatrix}0.93 & 0.07\\ 0.05 & 0.95\end{pmatrix}$, then $(\boldsymbol{u}^B)^{\mu} = \begin{pmatrix}0.93/0.07 & 1\\ 1 & 0.95/0.05\end{pmatrix} = \begin{pmatrix}13 & 1\\ 1 & 19\end{pmatrix}$ for all μ. The results do not depend critically on the choice of prior parameters as long as they are in a reasonable range (Fig S21 Fig, S22 Fig).

Avoiding local minima

To avoid local minimum, the evidence was calculated 20 times for each model with random initial parameters and the result with a larger evidence was selected. Initial values for transition matrices were generated by using Dirichlet distribution: $\boldsymbol{A}$ with parameters $u_a = 0.3, u_{ad} = 200$; $\boldsymbol{B}$ with parameters $u_b = 1, u_{ad} = 20$. u_{ad}, u_{bd} are used to generate the diagonal elements of transition matrices.

Computation time

Computation time depends on the length of data, the number of models to be tested, and the number of repeat (to avoid local minimum). For example, the analysis of one time trace with $T_{obs}/\Delta t$= 4400, K=1, 2, and 3, and 20 repeats takes $\sim$ 3 min whereas the same test but with $T_{obs}/\Delta t$= 8800 takes $\sim$6 min on Macbook pro 13 (3 GHz intel core i7). Linear dependence of analysis time on $T_{obs}/\Delta t$ is expected because each implementation requires execution of DCMM. The running time scales linearly with the length of data as it involves a similar procedure of parameter estimation as HMM [1]. F converged usually after 10 iterations in our test conditions except the case when poor guess for u_a, u_{ad}, u_b, u_{bd} was used on purpose while testing the algorithm (S21 Fig, S22 Fig). All the implementations of algorithm and data analysis were conducted by using our custom-code written in python with the following libraries: Matplotlib [4], Numpy [5], Scipy [6], IPython [7], Scikit-learn [8] and Cython [9].

EFFICACY OF VB-DCMM ASSESSED BY THE LAW OF LARGE NUMBER

To assess the efficacy of VB-DCMM in identifying dynamic disorder (hidden internal state) of a given time trace, we divided an ensemble of heterogeneous time traces into shorter homogeneous traces by using the information of internal states in $x^{\text{model}}(t)$, and calculated the distribution of $\varphi_{20} \equiv \sigma_{20}/\mu_{20}$ of dwell times, where the subscript 20 means that 20 consecutive data of dwell times along the time traces are used in evaluating the standard deviation ($\sigma^2_{20} =$

$\frac{1}{20}\sum_{i=1}^{20}(\tau_i - \mu_{20})^2$) and the mean ($\mu_{20} = \frac{1}{20}\sum_{i=1}^{20}\tau_i$). It is expected that $\varphi_{20} = 1$ for the time traces generated from a completely homogeneous Markov process; however, $\varphi_{20} > 1$ when it is evaluated at the boundaries where different internal states coexist. Thus the distribution of φ_{20} will be sharply defined as $P(\varphi_{20}) \sim \delta(\varphi_{20} - 1)$ if a heterogeneous trace is correctly decomposed into several pieces of homogeneous traces, so that each piece contains only one internal state. Indeed, after the decomposition of original time trace the histogram of φ_{20} become narrower and more Gaussian like (S23 Fig A–C). Test on synthetic data generated using $K = 3$ also shows a similar trend (S24 Fig). Next we analyzed H-DNA traces with more than 3 interconversion events between internal states (S23 Fig D–F). (D_{conf}, D_{int}) values of these traces are in the region where the synthetic traces displaying $\langle\chi\rangle \sim 0.9$.

In Markov model, the transition probability from an observable state a to b is estimated as $w_{a\to b} = k_{a\to b}\,\Delta t = n_{a\to b}/\sum_b n_{a\to b}$ ($n_{a\to b}$ is the actual number of transitions from a to b observed from a given trace) and the ratio between the standard deviation ($\sigma_{n_{a\to b}} = \sqrt{\langle(\delta n_{a\to b})^2\rangle} = \sqrt{\langle n_{a\to b}\rangle}$) and mean ($\mu_{a\to b} = \langle n_{a\to b}\rangle$) of the number of transitions $n_{a\to b}$ satisfies $\varphi_{n_{a\to b}} = \sigma_{n_{a\to b}}/\mu_{n_{a\to b}} = 1/\sqrt{\langle n_{a\to b}\rangle} \sim 1/\sqrt{n_{a\to b}}$. Thus, we expect $\varphi_{k_{a\to b}} \sim \varphi_{n_{a\to b}} \sim 1/\sqrt{n_{a\to b}} \sim 1/\sqrt{\tau_{int}/\tau_{conf}}$. Since ∼4 fold difference in $k^{(\mu)}_{a\to b}$ and $k^{(\nu)}_{a\to b}$ (with $\mu \neq \nu$) is sufficient for the reliable detection of internal states (Fig. 4A, S1 Fig), VB-DCMM is expected to work for $\varphi_{k_{a\to b}} \sim \varphi_{n_{a\to b}} \lesssim 1/4$ which leads to a requirement of time scale separation between τ_{int} and τ_{conf} as $\tau_{int}/\tau_{conf} \gtrsim 16$ (or $D_{\text{int}} \gtrsim 4$ (Eq. (5))). Indeed when all synthetic data were plotted with two metrics D_{conf} and D_{int}, all the data with $D_{\text{int}} \gtrsim 4$ show high $\langle\chi\rangle$ for $D_{\text{conf}} \gtrsim 2$ (Eq. (4)) (Fig. 5). Large D_{conf} is important for the internal states to be discernible, whereas large D_{int} is required for accurate estimation of k. The performance of algorithm relies on these two factors.

OTHER APPROACHES

Markov Chain Monte Carlo (MCMC) technique

As an alternative way of calculating the evidence, Bayesian version of DCMM using MCMC method has previously been developed for credit portfolio modeling [10]. They, however, used Bayesian inference to calculate posterior distribution of model parameters, instead of selecting a model with optimal number of internal states, and determined the number of internal states based on well-accepted economic cycle fluctuation model. This approach is not applicable when solid knowledge on internal states is not available. Also, they have used constant value for all prior parameters without investigating the effect of prior parameters on the analysis. Furthermore, unlike VB-DCMM, MCMC method does not offer analytical expression for the evidence, which makes it difficult to select prior parameters (or to incorporate prior information).

Comparison with Infinite Aggregated Markov Model (iAMM)

There has been a study developing a method that can detect the presence of hidden states by analyzing the dynamic pattern of single ion channel data using (sticky) infinite aggregated Markov model (iAMM) with nonparametric Bayesian method [11, 12]. VB-DCMM differs from iAMM in several ways and sometimes can be more advantageous: (1) iAMM uses Markov chain Monte Carlo method whereas VB-DCMM employs the variational Bayes method which is computationally less expensive. (2) In iAMM, the aggregated Markov model (AMM) is the basic structure in which only a single Markov Chain exists. The model aims to detect distinct transition rates from a signal layer of signal. In fact, one can map DCMM onto the structure of AMM by *flattening* the two layers of states in DCMM (internal and observable states) into a sequence of one state. For example, a data structure of DCMM retaining two internal states X_1, X_2 and two observables O_1, O_2 can be mapped onto four states in AMM as follows: $Z_1 = (X_1, O_1), Z_2 = (X_1, O_2), Z_3 = (X_2, O_1)$, and $Z_4 = (X_2, O_2)$ (S25 Fig A). While the transition between two dynamic patterns in DCMM are more strictly regulated, so that the transition rates $k_{Z_1\to Z_4}, k_{Z_4\to Z_1}, k_{Z_2\to Z_3}$, and $k_{Z_3\to Z_2}$ are practically zero as the transitions of observables are slaved to the internal state, AMM does not impose such condition. Although AMM could be more flexible in accommodating possible transitions, and could accurately predict the sequence of internal states under very carefully selected the prior parameters (see S25 Fig C), we found that the results obtained from iAMM analysis against our synthetic data was highly sensitive to the prior parameters being selected (S25 Fig D), and that in the most of prior parameters, the traces predicted by iAMM ($z^{\text{iAMM}}(t)$), predicting unwanted frequent transitions between the states, do not match with the synthetic data ($z(t)$), which gives rise to a low χ value ($\chi = \frac{1}{T}\sum_{t=1}^{T}\delta_{z(t),z^{\text{iAMM}}(t)}$).

The persistent dynamic pattern as shown in H-DNA and preQ$_1$-riboswitch [13] dynamics can be better modeled with VB-DCMM whose result is not sensitive to the choice of prior parameters (S21 Fig, S22 Fig).

* hyeoncb@kias.re.kr

[1] A. Berchtold, Technical Report, Washington Univ. (1999).
[2] C. M. Bishop, *Pattern Recognition and Machine Learning* (Springer, 2006).
[3] S. Ji, B. Krishnapuram, and L. Carin, IEEE Trans. Pattern Anal. Mach. Intell. **28**, 522 (2006).
[4] J. D. Hunter, Comput. Sci. Eng. **9**, 90 (2007).
[5] S. van der Walt, S. Colbert, and G. Varoquaux, Comput. Sci. Eng. **13**, 22 (2011).
[6] E. Jones, T. Oliphant, P. Peterson, et al., *SciPy: Open source scientific tools for Python* (2001–), URL `http://www.scipy.org/`.
[7] F. Pérez and B. E. Granger, Comput. Sci. Eng. **9**, 21 (2007), URL `http://ipython.org`.
[8] F. Pedregosa, G. Varoquaux, A. Gramfort, V. Michel, B. Thirion, O. Grisel, M. Blondel, P. Prettenhofer, R. Weiss, V. Dubourg, et al., J. Mach. Learn. Res. **12**, 2825 (2011).
[9] S. Behnel, R. Bradshaw, C. Citro, L. Dalcin, D. Seljebotn, and K. Smith, Comput. Sci. Eng. **13**, 31 (2011).
[10] M. Fitzpatrick and D. Marchev, Stat. and Comput. **23**, 467 (2013).
[11] E. B. Fox, E. B. Sudderth, M. I. Jordan, and A. S. Willsky, in *Proc. International Conference on Machine Learning* (2008).
[12] K. Hines, J. Bankston, and R. Aldrich, Biophys. J. **108**, 540 (2015).
[13] A. J. Rinaldi, P. E. Lund, M. R. Blanco, and N. G. Walter, Nat. Commun. **7**, 1 (2016).
[14] B. J. Frey and D. Dueck, Science **315**, 972 (2007).

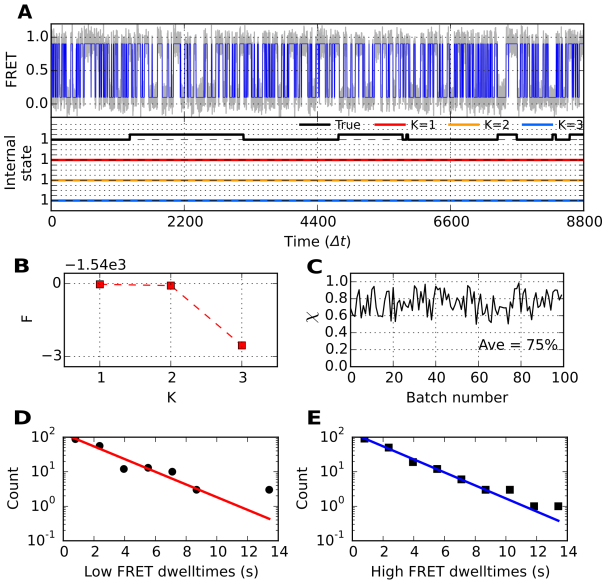

S1 Fig: VB-DCMM analysis on synthetic data generated with the following parameters: $K^{\rm true} = 2$, $\gamma^{(1)\to(2)}\Delta t = \gamma^{(2)\to(1)}\Delta t = 0.001, k^{(1)}_{L\to H}\Delta t = k^{(1)}_{H\to L}\Delta t = 0.05, k^{(2)}_{L\to H}\Delta t = k^{(2)}_{H\to L}\Delta t = 0.0025$. (A) (Top) : Gray line is FRET trace and blue line is noise-filtered FRET obtained by using HMM. (Bottom) : True internal state trace (black) and estimated internal state traces by assuming the model with different K (red: $K = 1$, orange: $K = 2$, blue: $K = 3$). (B) $F(K)$ from the result of VB-DCMM analysis. (C) The accuracy of the model prediction on 100 traces generated under identical condition with the FRET trace shown in (A). (D) Low FRET dwell time histogram and (E) high FRET dwell time histogram obtained from the FRET trace in (A). The solid line denotes a single exponential fit.

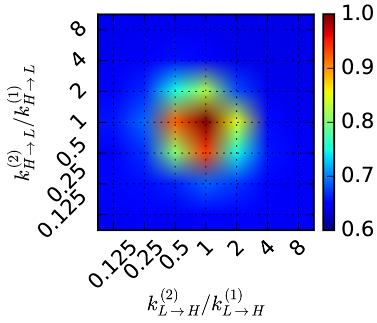

S2 Fig: Re-calculated accuracy of model prediction ($\langle\chi\rangle$) by assuming "$K = 1$" (i.e., assuming $x(t)^{\rm true} = 1$ for all t in Eq.(3)) for the same set of parameters used to calculate Fig. 4A.

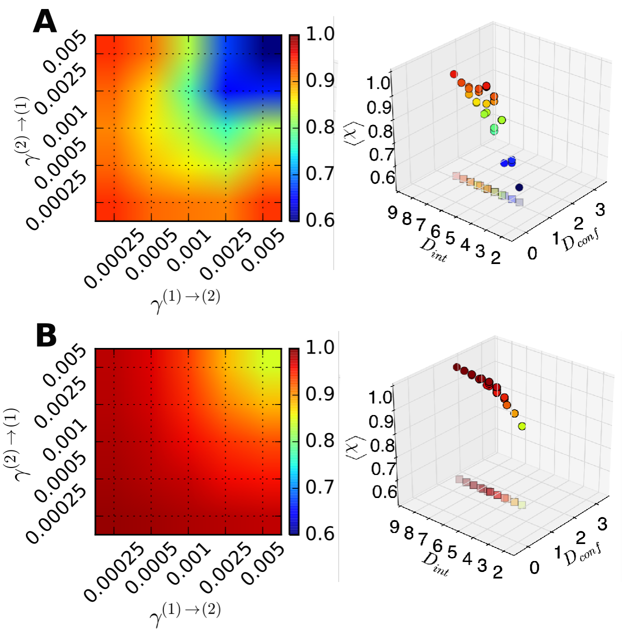

S3 Fig: Systematic validation of VB-DCMM on synthetic data generated under various conditions with $T_{obs}/\Delta t = 8800$. Color code denotes the accuracy of the model prediction in terms of $\langle \chi \rangle$, averaged over 100 traces for each condition. Same analysis were performed with Fig. 4B under different conditions. (A) $\langle \chi \rangle$ under varying $\gamma^{(1)\to(2)}$ and $\gamma^{(2)\to(1)}$ with $K^{\text{true}} = 2$, $k^{(1)}_{L\to H}\Delta t = k^{(1)}_{H\to L}\Delta t = 0.05$, $k^{(2)}_{L\to H}\Delta t = 0.025$, $k^{(2)}_{H\to L}\Delta t = 0.1$. (B) $\langle \chi \rangle$ under varying $\gamma^{(1)\to(2)}$ and $\gamma^{(2)\to(1)}$ with $K^{\text{truetrue}} = 2$, $k^{(1)}_{L\to H}\Delta t = k^{(1)}_{H\to L}\Delta t = 0.05$, $k^{(2)}_{L\to H}\Delta t = 0.1$, $k^{(2)}_{H\to L}\Delta t = 0.2$.

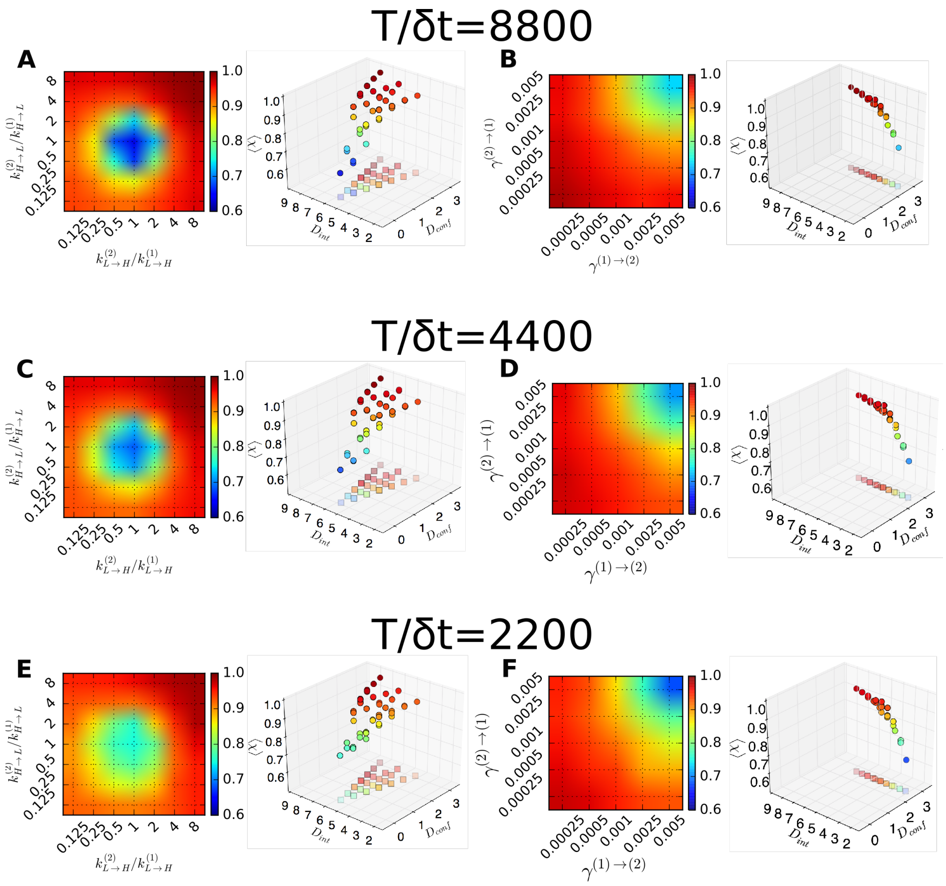

S4 Fig: Accuracy of VB-DCMM on synthetic data with $T_{obs}/\Delta t = 4400$ and 2200. Color code denotes the accuracy of the model prediction in terms of $\langle \chi \rangle$, averaged over 100 traces for each condition, under varying $k^{(2)}_{L \to H}, k^{(2)}_{H \to L}$. (A-B) Results with $T_{obs}/\Delta t = 8800$. Same graphs from Fig. 4 are showed again for clarity. (C-D) Results with $T_{obs}/\Delta t = 4400$ and (E-F) Results with $T_{obs}/\Delta t = 2200$. Same analysis with Fig. 4 were performed.

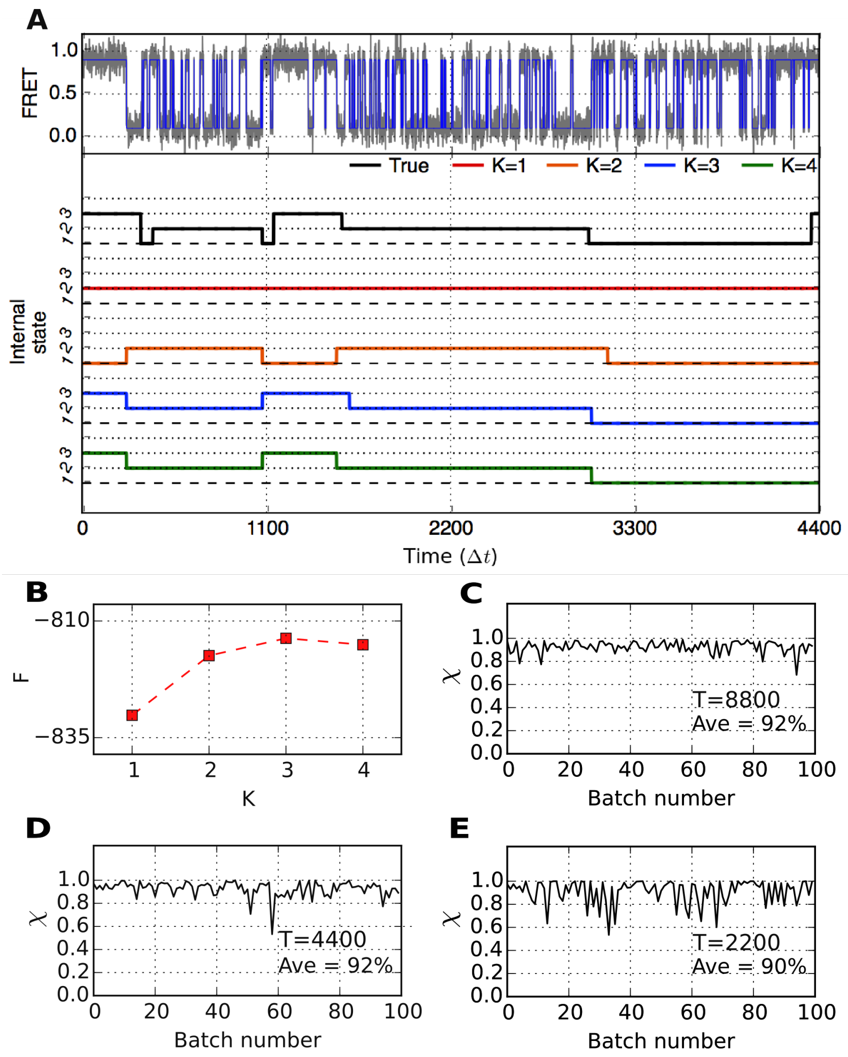

S5 Fig: VB-DCMM analysis on synthetic data having three internal states generated with the following parameters: $K^{\mathrm{true}} = 3$, $\gamma^{(1)\to(2)}\Delta t = \gamma^{(1)\to(3)}\Delta t = \gamma^{(2)\to(3)}\Delta t = \gamma^{(3)\to(2)}\Delta t = \gamma^{(3)\to(1)}\Delta t = \gamma^{(2)\to(1)}\Delta t = 0.0005, k^{(1)}_{L\to H}\Delta t = 0.1, k^{(1)}_{H\to L}\Delta t = 0.04, k^{(2)}_{L\to H}\Delta t = 0.04, k^{(2)}_{H\to L}\Delta t = 0.1, k^{(3)}_{L\to H}\Delta t = 0.008, k^{(3)}_{H\to L}\Delta t = 0.012$. (A) (Top) : Gray line indicates FRET trace and blue line is noise-filtered FRET obtained by using HMM. (Bottom) : True internal state trace (Black) and estimated internal state traces (red: $K = 1$, orange: $K = 2$, blue: $K = 3$, green: $K = 4$). (B) $F(K)$ from VB-DCMM analysis. (C) χ on 100 traces with $T_{obs}/\Delta t$= 8800, (D) with $T_{obs}/\Delta t$= 4400, and (E) with $T_{obs}/\Delta t$= 2200.

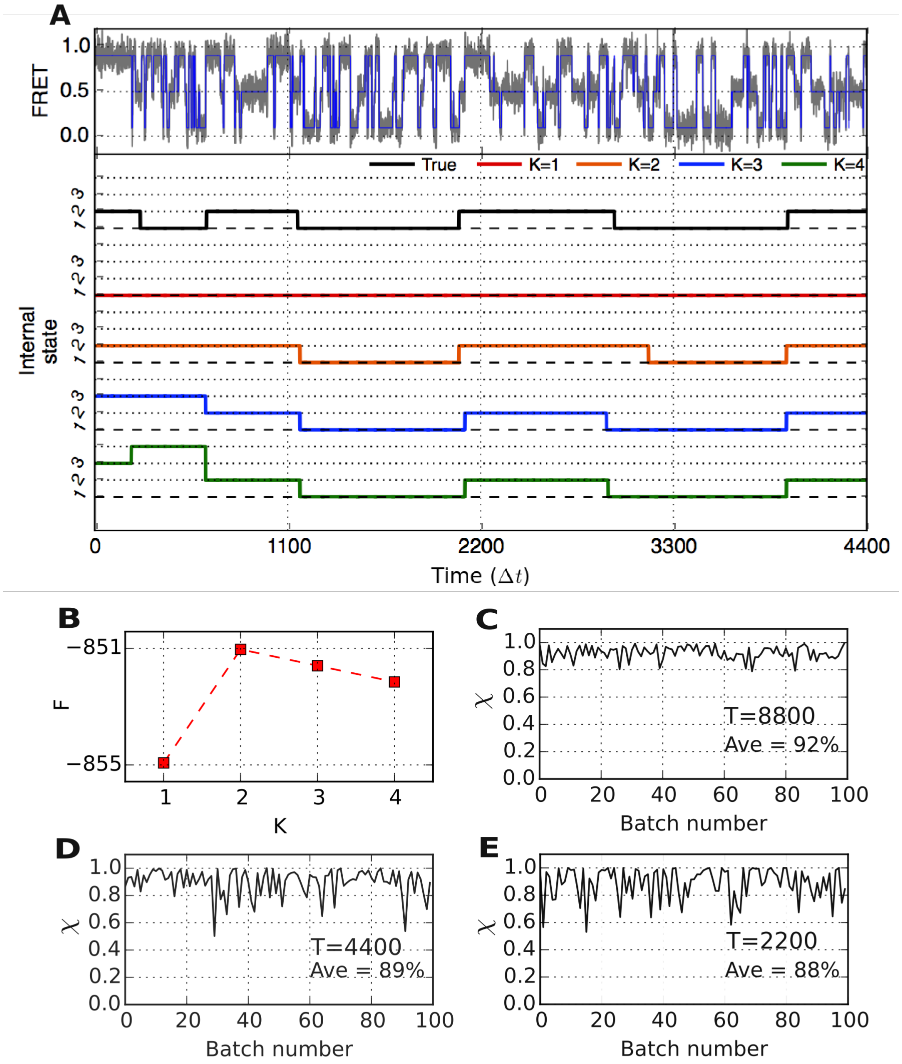

S6 Fig: VB-DCMM analysis on synthetic data having 3 observables generated with following parameters: $K^{\text{true}} = 2$, $\gamma^{(1)\to(2)}\Delta t = \gamma^{2\to 2}\Delta t = 0.001, k^{(1)}_{L\to M}\Delta t = 0.015, k^{(1)}_{L\to H}\Delta t = 0.023, k^{(1)}_{M\to L}\Delta t = 0.032, k^{(1)}_{M\to H}\Delta t = 0.05, k^{(1)}_{H\to L}\Delta t = 0.03, k^{(1)}_{H\to M}\Delta t = 0.014, k^{(2)}_{L\to M}\Delta t = 0.058, k^{(2)}_{L\to H}\Delta t = 0.065, k^{(2)}_{M\to L}\Delta t = 0.021, k^{(2)}_{M\to H}\Delta t = 0.004, k^{(2)}_{H\to L}\Delta t = 0.0093, k^{(2)}_{H\to M}\Delta t = 0.014$. (A) (Top) : Gray line indicates FRET trace and blue line is noise-filtered FRET obtained by using HMM. (Bottom) : True internal state trace (Black) and estimated internal state traces (red: $K = 1$, orange: $K = 2$, blue: $K = 3$, green: $K = 4$). (B) $F(K)$ from VB-DCMM analysis. (C) χ on 100 traces with $T_{obs}/\Delta t$= 8800, (D) with $T_{obs}/\Delta t$= 4400, and (E) with $T_{obs}/\Delta t$= 2200.

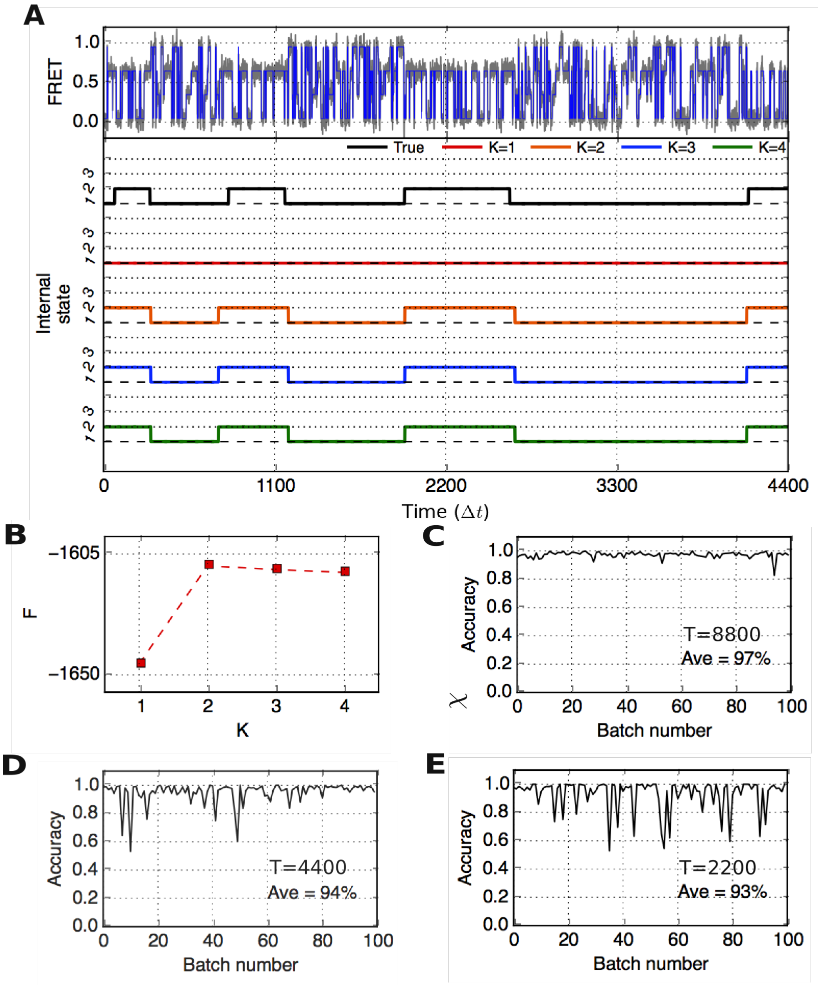

S7 Fig: VB-DCMM analysis on synthetic data having 4 observables (o=1, 2, 3, and 4) when internal state x=1, or having 2 (o=1, 3) observables when x=2. Following parameters are used to generate synthetic data: $K^{\text{true}} = 2$, $\gamma^{(1)\to(2)}\Delta t = \gamma^{2\to 2}\Delta t = 0.001, k^{(1)}_{1\to 2}\Delta t = 0.015, k^{(1)}_{1\to 3}\Delta t = 0.023, k^{(1)}_{1\to 4}\Delta t = 0.05, k^{(1)}_{2\to 1}\Delta t = 0.032, k^{(1)}_{2\to 3}\Delta t = 0.05, k^{(1)}_{2\to 4}\Delta t = 0.01, k^{(1)}_{3\to 1}\Delta t = 0.03, k^{(1)}_{3\to 2}\Delta t = 0.014, k^{(1)}_{3\to 4}\Delta t = 0.03, k^{(1)}_{4\to 1}\Delta t = 0.1, k^{(1)}_{4\to 2}\Delta t = 0.02, k^{(1)}_{4\to 3}\Delta t = 0.01, k^{(2)}_{1\to 3}\Delta t = 0.085, k^{(2)}_{3\to 1}\Delta t = 0.063$. To make only o=1, 3 appears when $x = 2$, the transition rates $k^{(2)}_{i\to j}$ set to zero when i or j is 2 or 4. (A) (Top) : Gray line indicates FRET trace and blue line is noise-filtered FRET obtained by using HMM. (Bottom) : True internal state trace (Black) and estimated internal state traces (red: $K = 1$, orange: $K = 2$, blue: $K = 3$, green: $K = 4$). (B) $F(K)$ from VB-DCMM analysis. (C) χ on 100 traces with $T_{obs}/\Delta t$= 8800, (D) with $T_{obs}/\Delta t$= 4400, and (E) with $T_{obs}/\Delta t$= 2200.

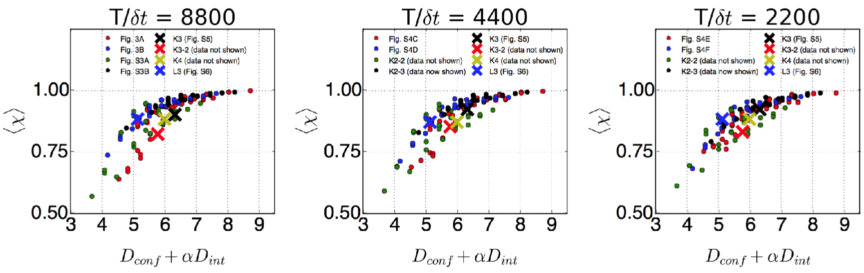

S8 Fig: Average accuracy of the model prediction $\langle\chi\rangle$ versus $D_{\text{tot}} = D_{\text{conf}} + 0.8D_{\text{int}}$ from various synthetic data, where results are shown in Fig. 4, S3 Fig ($T_{obs}/\Delta t$=8800), S4 Fig ($T_{obs}/\Delta t$=4400, 2200).

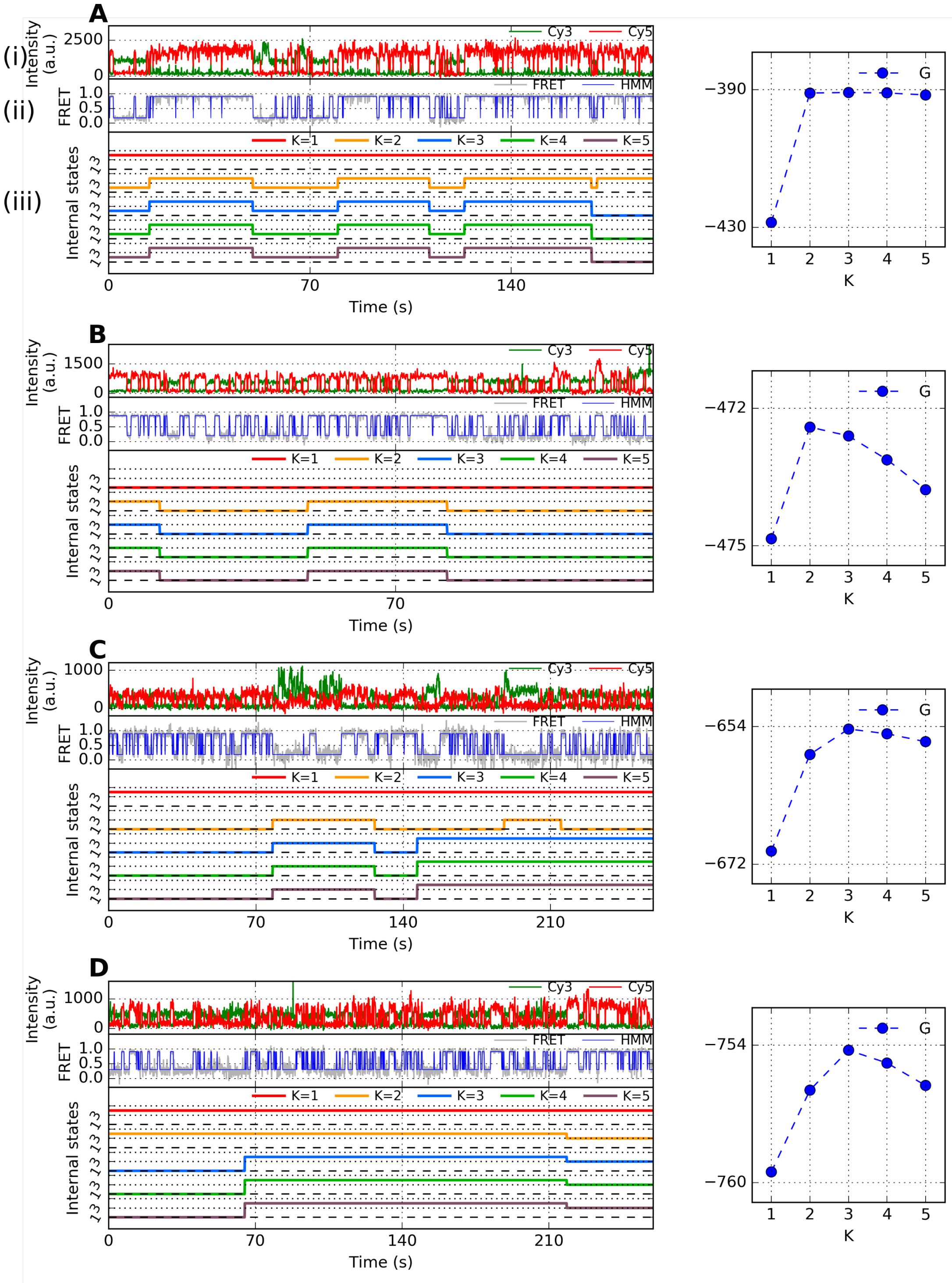

S9 Fig: Representative time traces of H-DNA dynamics and their analysis using VB-DCMM at [Na$^+$]= 50 mM. (A) (i) Representative fluorescence signal and (ii) their FRET state. (iii) Internal states estimated for $K = 1, 2, \ldots 5$. Right panel shows $G(K)$ (blue circle). (B, C, D) Other representative time traces and their lower bound obtained under the same experimental condition.

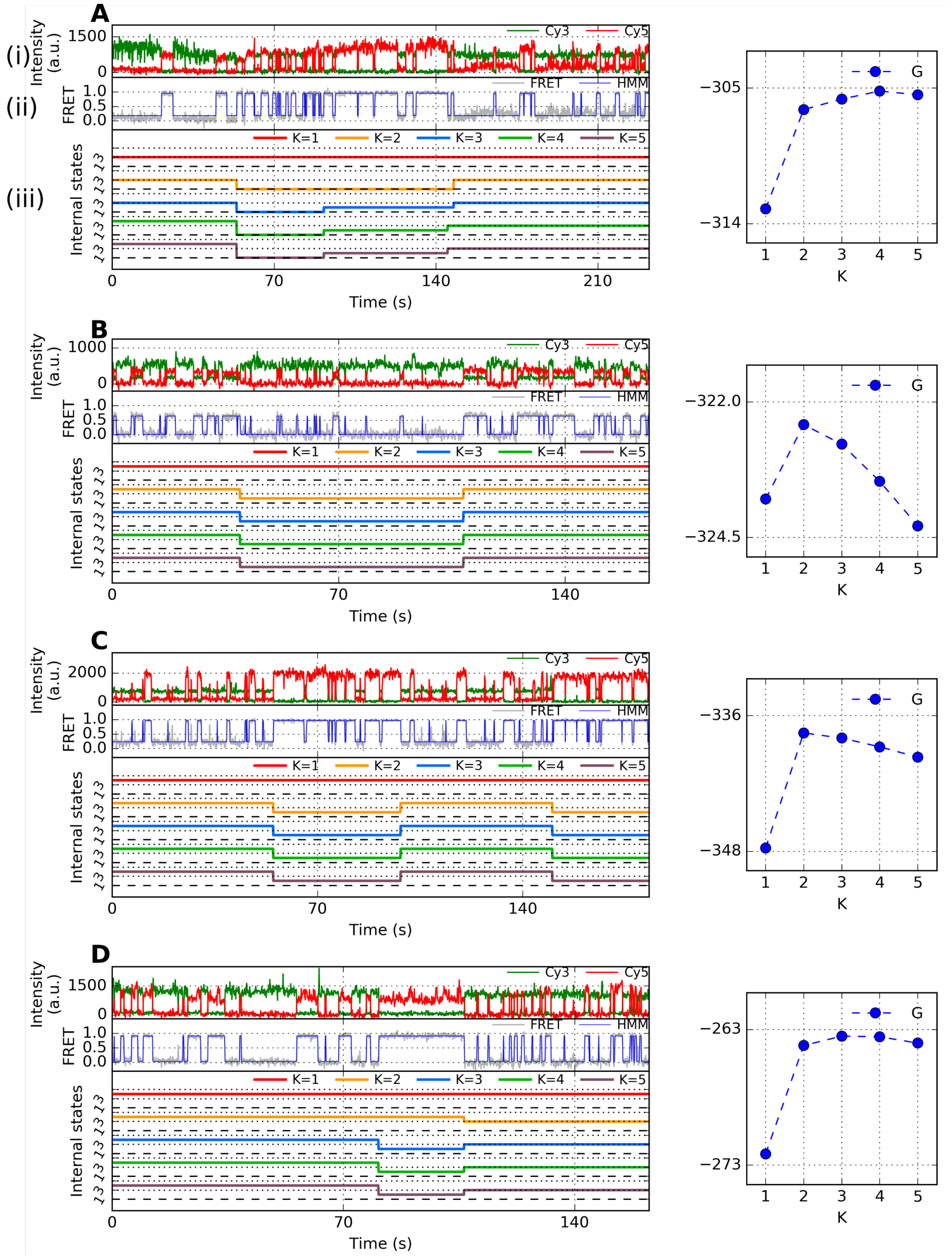

S10 Fig: Representative time traces of H-DNA dynamics and their analysis using VB-DCMM at [Na^+]= 26 mM. (A) (i) Representative fluorescence signal and (ii) their FRET state. (iii) Internal states estimated for $K = 1, 2, \ldots 5$. Right panel shows $G(K)$ (blue circle).. (B, C, D) Other representative time traces and their lower bound obtained under the same experimental condition.

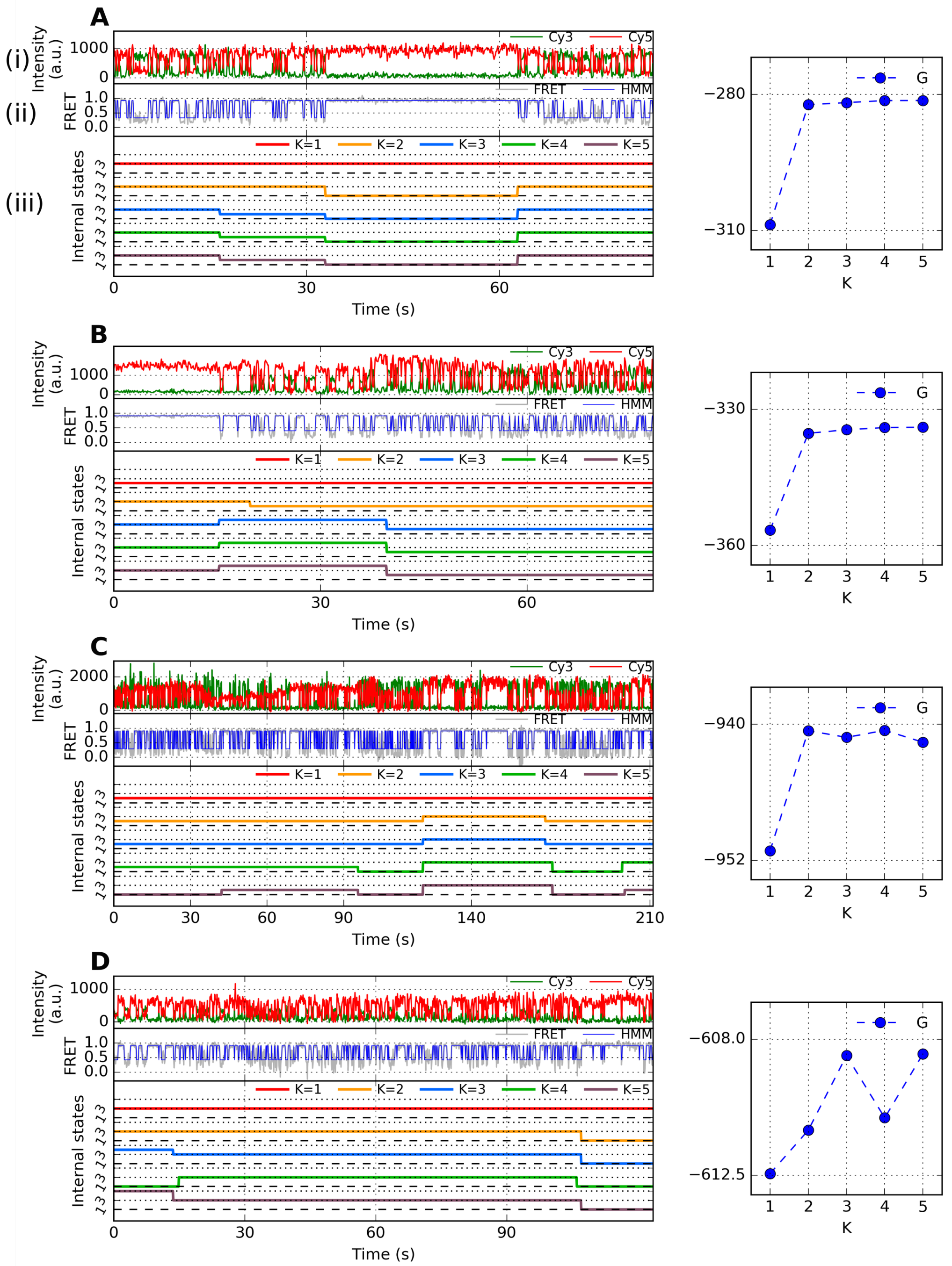

S11 Fig: Representative time traces of H-DNA which display more than two internal states within the trace ($K^* > 2$) and their analysis using VB-DCMM at [Na^+]= 100 mM. (A) (i) Representative fluorescence signals and (ii) their FRET state. (iii) Internal states estimated for $K = 1, 2, \ldots 5$. Right panel shows $G(K)$ (blue circle). (B-D) Other representative time trace and their lower bound obtained under the same experimental condition. Decrease of $G(K)$ at $K = 3$ in (C) and at $K = 4$ in (D) is due to trapping in local minimum (In the analysis of H-DNA, the best solution was selected after applying VB-DCMM 20 times with random initial conditions for each K).

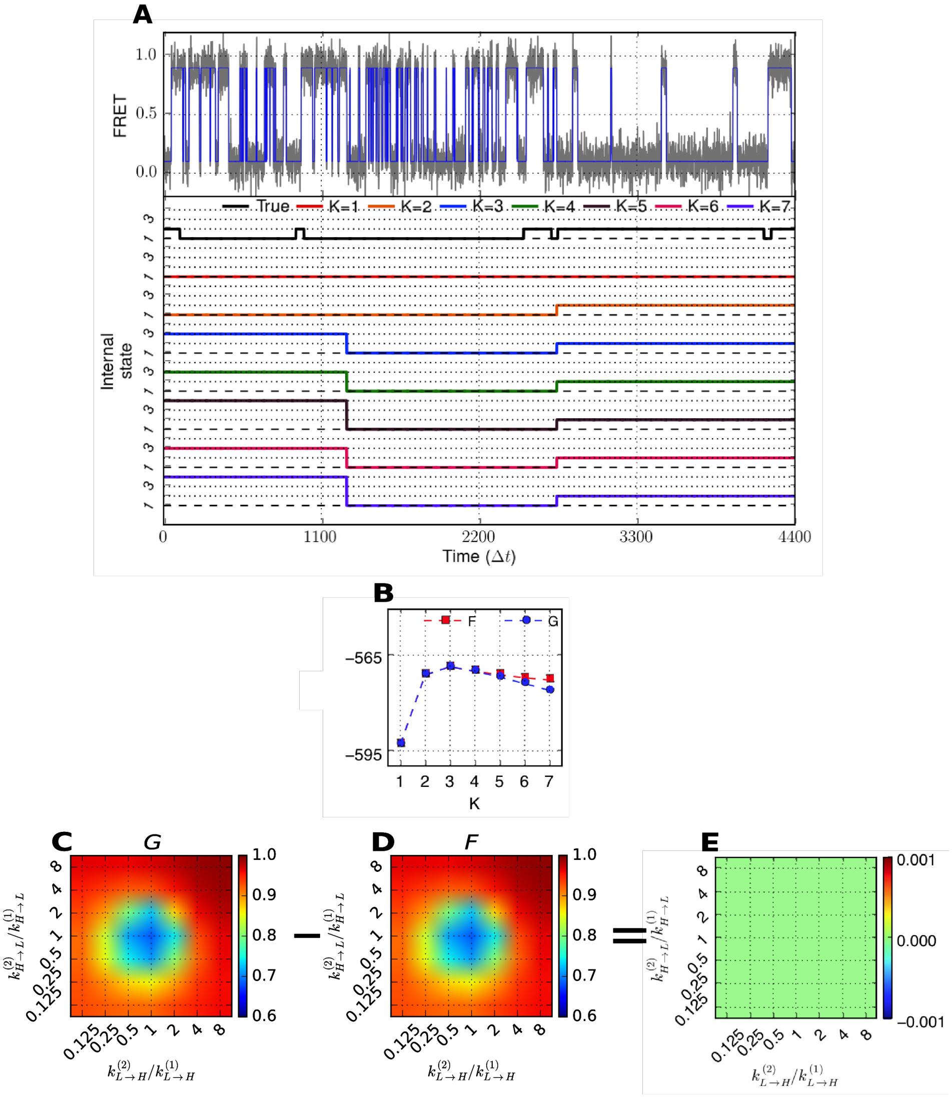

S12 Fig: VB-DCMM analysis on synthetic data generated with following parameters: $T_{obs}/\Delta t$= 4400, $K^{\text{true}} = 2$, $\gamma^{(1)\to(2)}\Delta t = \gamma^{(2)\to(1)}\Delta t = 0.001, k^{(1)}_{L\to H}\Delta t = k^{(1)}_{H\to L}\Delta t = 0.05, k^{(2)}_{L\to H}\Delta t = 0.00625, k^{(2)}_{H\to L}\Delta t = 0.0125$. (A) (Top): Gray line indicates FRET trace and blue line is noise-filtered FRET obtained after HMM analysis. (Bottom) : The traces of true internal state (Black) and estimated internal state (red: $K = 1$, orange: $K = 2$, blue: $K = 3$), green: $K = 4$, brown: $K = 5$, pink: $K = 6$, and purple: $K = 7$). (B) $F(K)$ (red, square) and $G(K)$ (blue, circle) from the results of VB-DCMM analysis. Accuracy of the model prediction using $\langle\chi\rangle$. In (C), the final model is selected using $G(K)$ whereas $F(K)$ is used in (D). (E) The difference between $\langle\chi\rangle$s from (C) and (D) is practically zero for all parameter range.

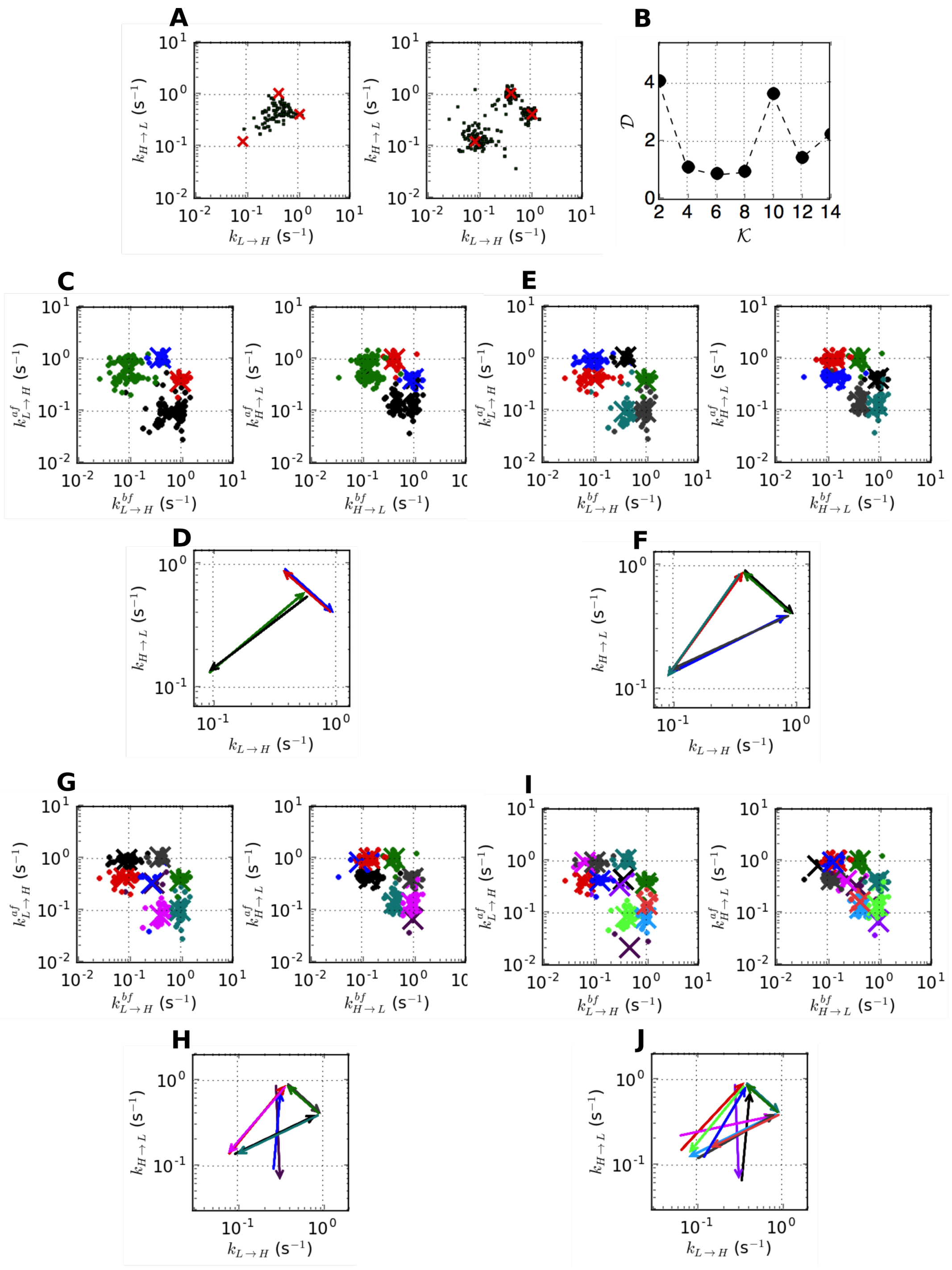

S13 Fig: Clustering synthetic data with three internal states ($K = 3$) with two observable states ($N = 2$). (A) The scatter plots of ($k_{L\to H}$, $k_{H\to L}$) before (left) and after (right) applying VB-DCMM from synthetic data generated with $K = 3$ (data from S5 FigA, D). Red crosses are the observable transition rates used to generate synthetic data which demonstrates how reliably VB-DCMM can recover the input transition rates. (B) The sum of pairing distances as a function of the number of centroids, $\mathcal{K}$ (See Methods). The clustering analysis was performed for $\mathcal{K} = 4$ (C-D), or $\mathcal{K} = 6$ (E-F).

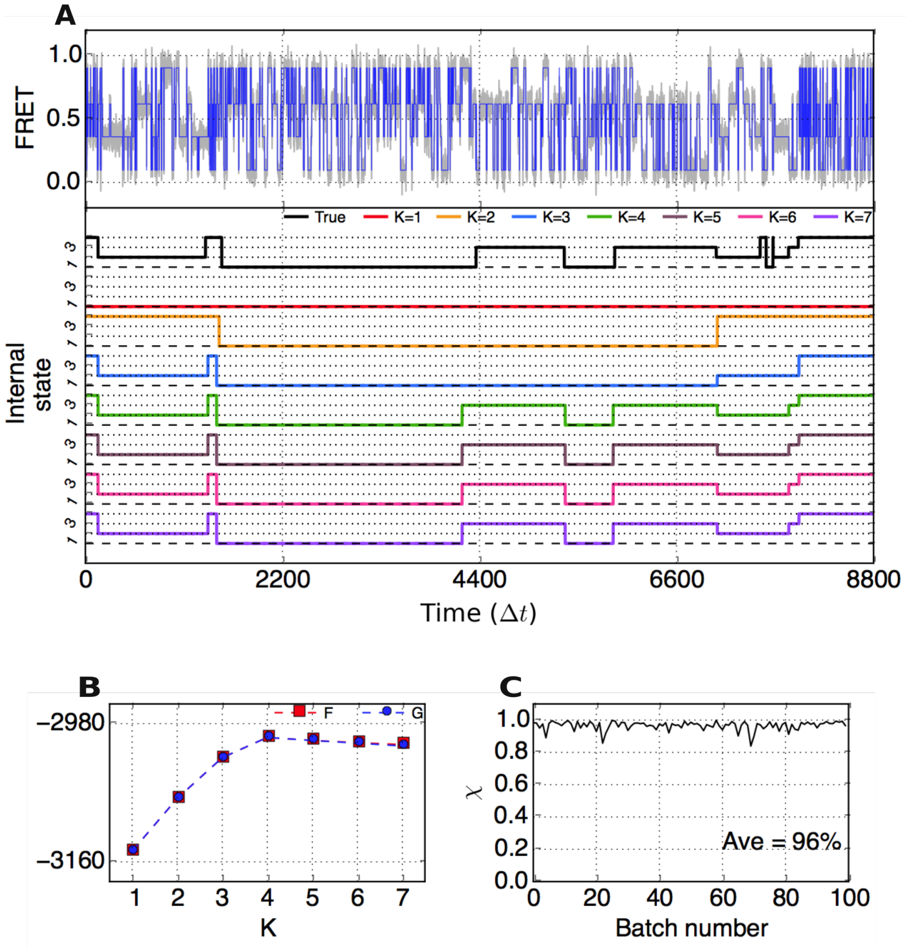

S14 Fig: VB-DCMM analysis on synthetic data having 4 internal states generated with the following parameters: $K^{\rm true} = 4$, $\gamma^{(1)\to(2)}\Delta t = \gamma^{(1)\to(3)}\Delta t = \gamma^{(2)\to(3)}\Delta t = \gamma^{(3)\to(2)}\Delta t = \gamma^{(3)\to(1)}\Delta t = \gamma^{(2)\to(1)}\Delta t = 0.00033, k^{(1)}_{1\to2}\Delta t = 0.015, k^{(1)}_{1\to3}\Delta t = 0.023, k^{(1)}_{1\to4}\Delta t = 0.015, k^{(1)}_{2\to1}\Delta t = 0.032, k^{(1)}_{2\to3}\Delta t = 0.05, k^{(1)}_{2\to4}\Delta t = 0.025, k^{(1)}_{3\to1}\Delta t = 0.03, k^{(1)}_{3\to2}\Delta t = 0.014, k^{(1)}_{3\to4}\Delta t = 0.06, k^{(1)}_{4\to1}\Delta t = 0.058, k^{(1)}_{4\to2}\Delta t = 0.065, k^{(1)}_{4\to3}\Delta t = 0.058, k^{(2)}_{1\to2}\Delta t = 0.058, k^{(2)}_{1\to3}\Delta t = 0.065, k^{(2)}_{1\to4}\Delta t = 0.058, k^{(2)}_{2\to1}\Delta t = 0.011, k^{(2)}_{2\to3}\Delta t = 0.004, k^{(2)}_{2\to4}\Delta t = 0.004, k^{(2)}_{3\to1}\Delta t = 0.0093, k^{(2)}_{3\to2}\Delta t = 0.014, k^{(2)}_{3\to4}\Delta t = 0.003, k^{(2)}_{4\to1}\Delta t = 0.06, k^{(2)}_{4\to2}\Delta t = 0.002, k^{(2)}_{4\to3}\Delta t = 0.02, k^{(3)}_{1\to2}\Delta t = 0.001, k^{(3)}_{1\to3}\Delta t = 0.05, k^{(3)}_{1\to4}\Delta t = 0.003, k^{(3)}_{2\to1}\Delta t = 0.07, k^{(3)}_{2\to3}\Delta t = 0.08, k^{(3)}_{2\to4}\Delta t = 0.01, k^{(3)}_{3\to1}\Delta t = 0.03, k^{(3)}_{3\to2}\Delta t = 0.01, k^{(3)}_{3\to4}\Delta t = 0.005, k^{(3)}_{4\to1}\Delta t = 0.004, k^{(3)}_{4\to2}\Delta t = 0.04, k^{(3)}_{4\to3}\Delta t = 0.002, k^{(4)}_{1\to2}\Delta t = 0.08, k^{(4)}_{1\to3}\Delta t = 0.002, k^{(4)}_{1\to4}\Delta t = 0.02, k^{(4)}_{2\to1}\Delta t = 0.011, k^{(4)}_{2\to3}\Delta t = 0.043, k^{(4)}_{2\to4}\Delta t = 0.11, k^{(4)}_{3\to1}\Delta t = 0.026, k^{(4)}_{3\to2}\Delta t = 0.07, k^{(4)}_{3\to4}\Delta t = 0.045, k^{(4)}_{4\to1}\Delta t = 0.1, k^{(4)}_{4\to2}\Delta t = 0.07, k^{(4)}_{4\to3}\Delta t = 0.03$, Here, sub-indexes i, j in $k^{(\mu)}_{i\to j}$ indicate observables o's with following FRET values: $o = 1$, FRET=0.1; $o = 2$, FRET=0.36; $o = 3$, FRET=0.62; $o = 4$, FRET=0.9. (A) (Top) : Gray line depicts FRET trace, and blue line is noise-filtered FRET obtained by using HMM. (Bottom) : True internal state trace (Black) and estimated internal state traces (red: $K = 1$, orange: $K = 2$, blue: $K = 3$, green: $K = 4$, brown: $K = 5$, pink: $K = 6$, and purple: $k = 7$). (B) $F(K)$ and $G(K)$ (Eq. S24) from VB-DCMM analysis. (C) χ on 100 traces with $T_{obs}/\Delta t$= 8800,

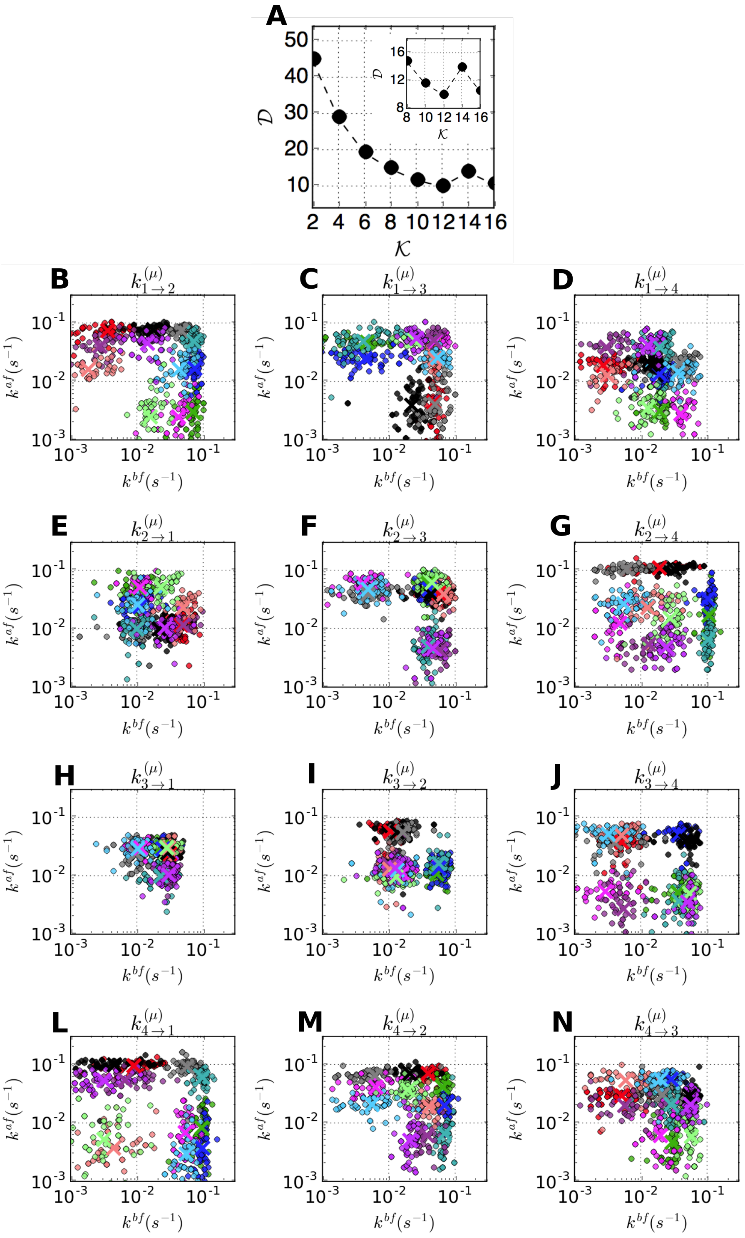

S15 Fig: Clustering synthetic data with four internal states ($K = 4$), and four observable states ($N = 4$). (A) The sum of pairing distances as a function of $\mathcal{K}$ the number of centroids, $\mathcal{K}$ (See Methods). The inset shows the region around $\mathcal{K} = 12$ of the same graph. (B) The result of clustering analysis performed when $\mathcal{K} = 12$ (B-N).

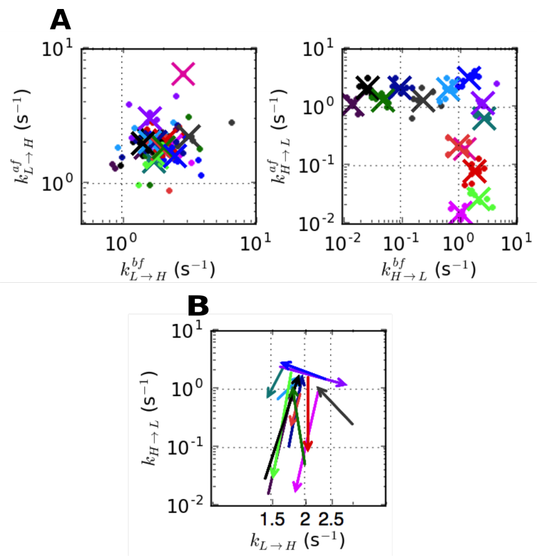

S16 Fig: Clustering H-DNA data ([Na$^+$] = 100 mM). (A-B) The clustering analysis used in Fig. 7C, D was performed for $\mathcal{K} = 14$.

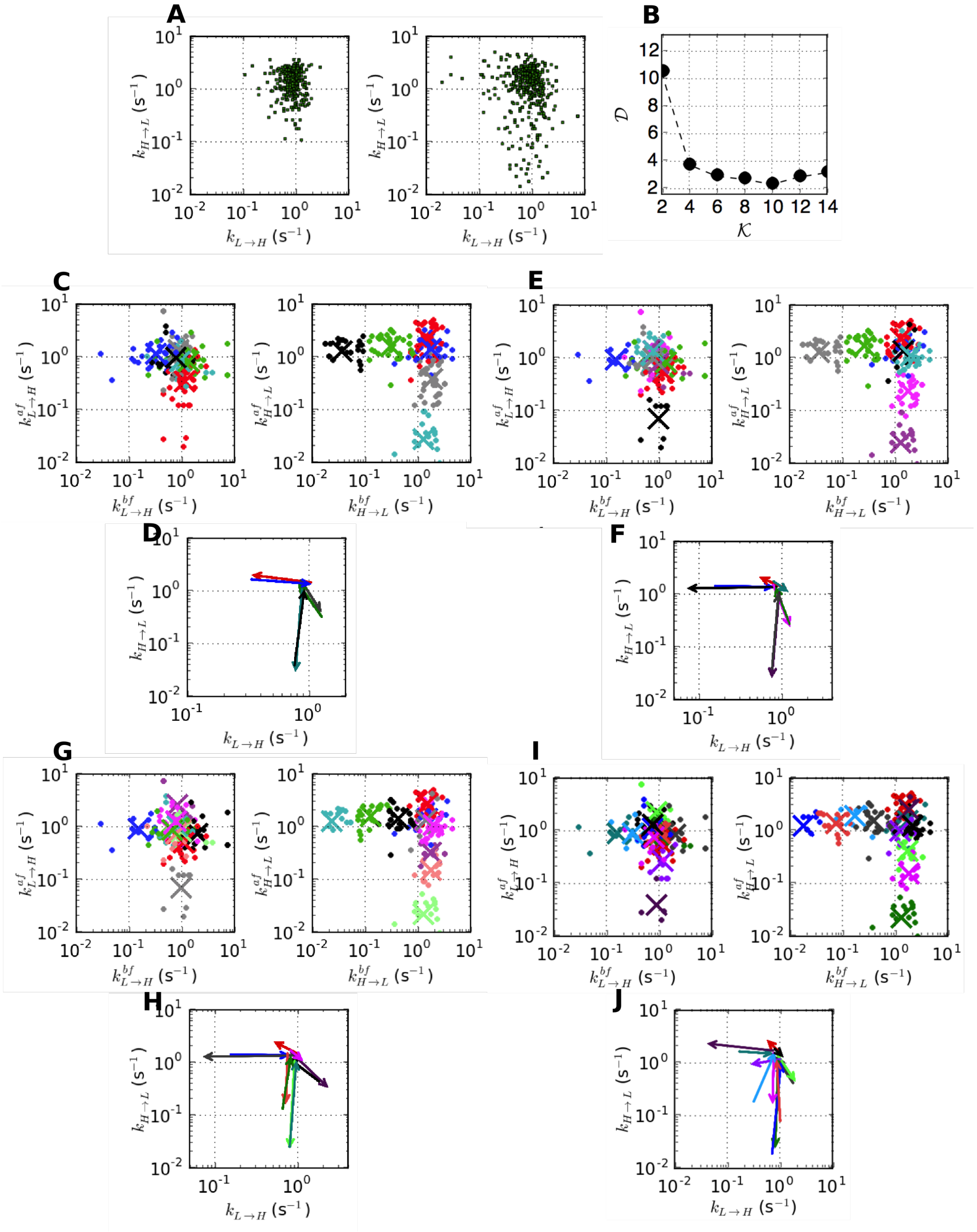

S17 Fig: Clustering H-DNA data ([Na$^+$] = 50 mM). (A) The scatter plots of ($k_{L\to H}$, $k_{H\to L}$) before (left) and after (right) applying VB-DCMM from [Na$^+$] = 50 mM H-DNA data. (B) The sum of pairing distances as a function of the number of centroids (See Methods). The clustering analysis used in Fig. 7C, D was performed for $\mathcal{K} = 6$ (C-D), $\mathcal{K} = 8$ (E-F), $\mathcal{K} = 10$ (G-H), or $\mathcal{K} = 12$ (I-J). Total 186 data points were used in each clustering analysis.

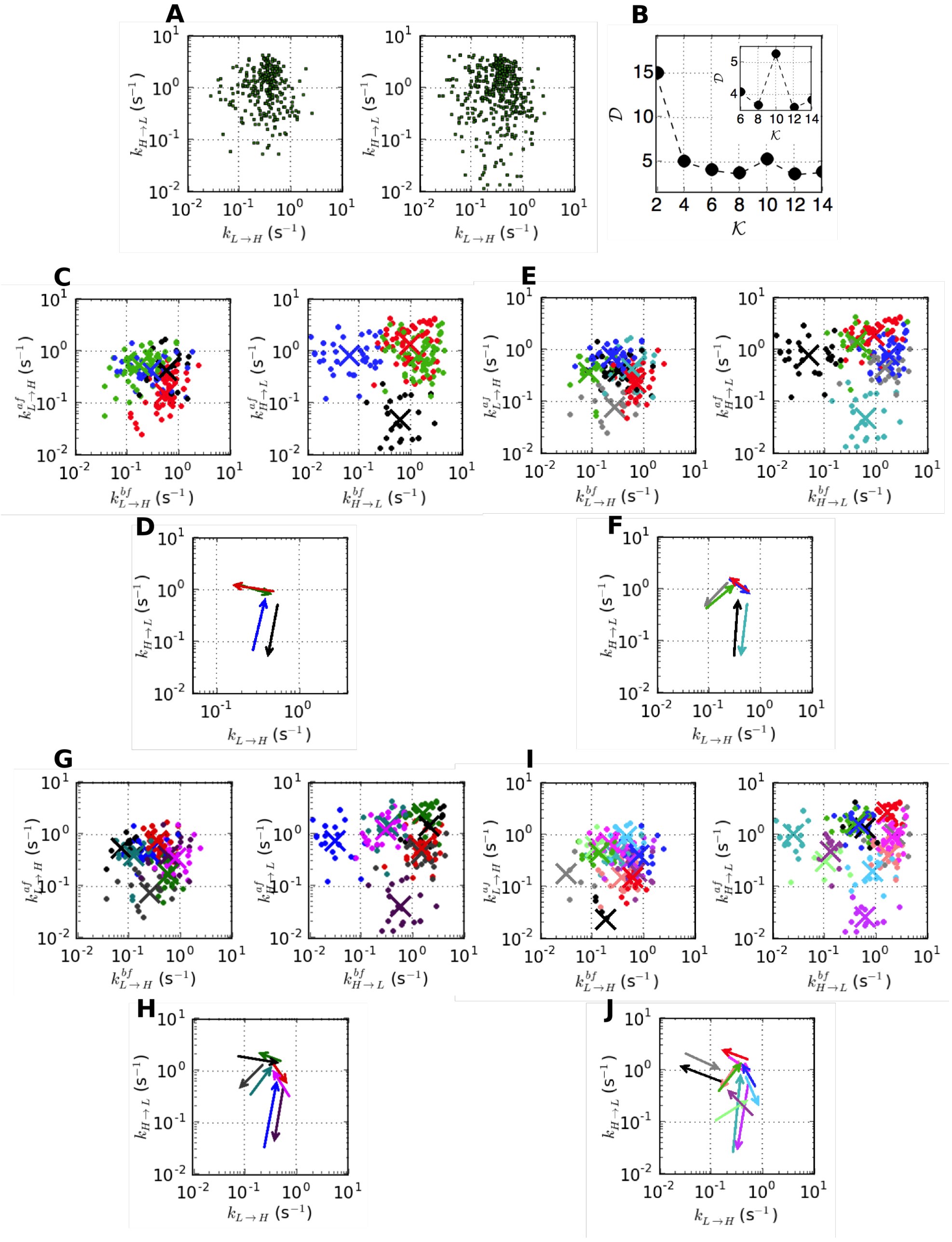

S18 Fig: Clustering H-DNA data ([Na$^+$] = 26 mM). (A) The scatter plots of ($k_{L\to H}$, $k_{H\to L}$) before (left) and after (right) applying VB-DCMM from [Na$^+$] = 26 mM H-DNA data. The inset shows the region around $\mathcal{K} = 12$ of the same graph. (B) The sum of pairing distances as a function of the number of centroids (See Methods). The clustering analysis used in Fig. 7C, D was performed by assuming $\mathcal{K} = 4$ (C-D), $\mathcal{K} = 6$ (E-F), $\mathcal{K} = 8$ (G-H), or $\mathcal{K} = 12$ (I-J). Total 185 data points were used in each analysis.

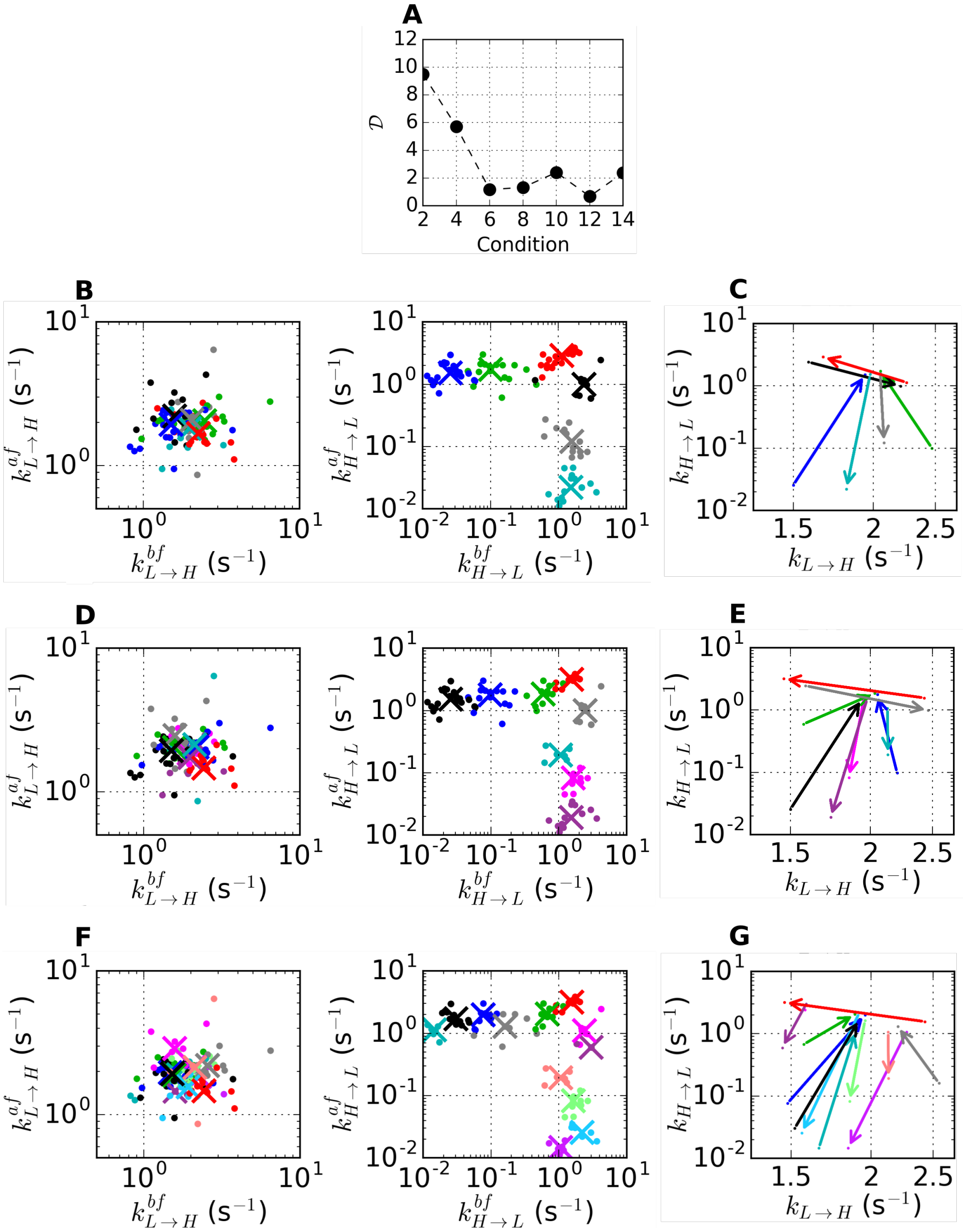

S19 Fig: H-DNA data ([Na$^+$] = 100 mM) analyzed with k-means clustering algorithm using "city block" distance ($L1$-distance). (A) The sum of pairing distances as a function of the number of centroids (See Methods). The clustering analysis done in Fig. 7C, D was performed again, but using "city block" distance. The clustering results are presented for $\mathcal{K} = 6$ (B-C), $\mathcal{K} = 8$ (D-E), and $\mathcal{K} = 12$ (F-G). Although $\mathcal{D}$ is minimized at $\mathcal{K} = 12$, 10 clusters out of 12 contain less than 10 data points, which is statistically not significant. Thus, $\mathcal{K} = 6$, corresponding to the suboptimal point of $\mathcal{D}$, could still be considered as the best solution.

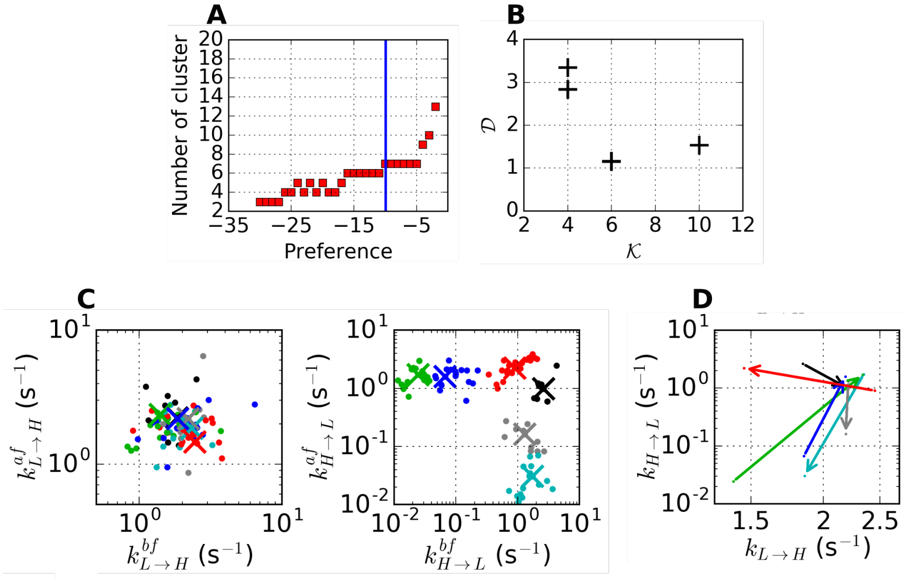

S20 Fig: Clustering results of H-DNA data ([Na$^+$] = 100 mM) from "affinity propagation" [14] that uses negative square-euclidean distance as the similarity metric. (A) The number of clusters calculated with varying "preference" parameter, where the preference denotes an input parameter (self-similarity) in the "affinity propagation" method. All the points were set to have the same preference value. Blue vertical line denotes median value of similarities between data points. (B) The sum of pairing distances as a function of $\mathcal{K}$. Only the results with even number of clusters in (A) are plotted. The clustering results at $\mathcal{K} = 6$ are shown in (C-D).

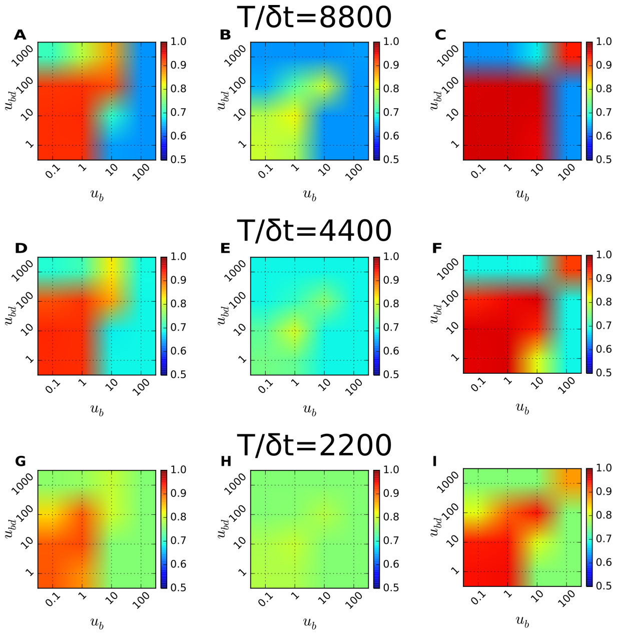

S21 Fig: Accuracy of the model prediction in terms of $\langle\chi\rangle$ under varying prior parameters u_b, and u_{bd}. To calculate the diagram 100 time traces were analyzed at each condition. $\langle\chi\rangle$ of each graph was evaluated for the data generated with the fixed parameters $\gamma^{(1)\to(2)}\Delta t = \gamma^{(2)\to(2)}\Delta t = 0.001, k^{(1)}_{L\to H}\Delta t = k^{(1)}_{H\to L}\Delta t = 0.05$, and (A) $k^{(2)}_{L\to H}\Delta t = 0.00625, k^{(2)}_{H\to L}\Delta t = 0.0125$, $T_{obs}/\Delta t$= 8800. (B) $k^{(2)}_{L\to H}\Delta t = 0.025, k^{(2)}_{H\to L}\Delta t = 0.1$, $T_{obs}/\Delta t$= 8800. (C) $k^{(2)}_{L\to H}\Delta t = 0.1, k^{(2)}_{H\to L}\Delta t = 0.2$, $T_{obs}/\Delta t$= 8800. (D-F) Identical conditions with (A-C) except $T_{obs}/\Delta t$= 4400. (G-I) Identical conditions with (A-C) except $T_{obs}/\Delta t$= 2200.

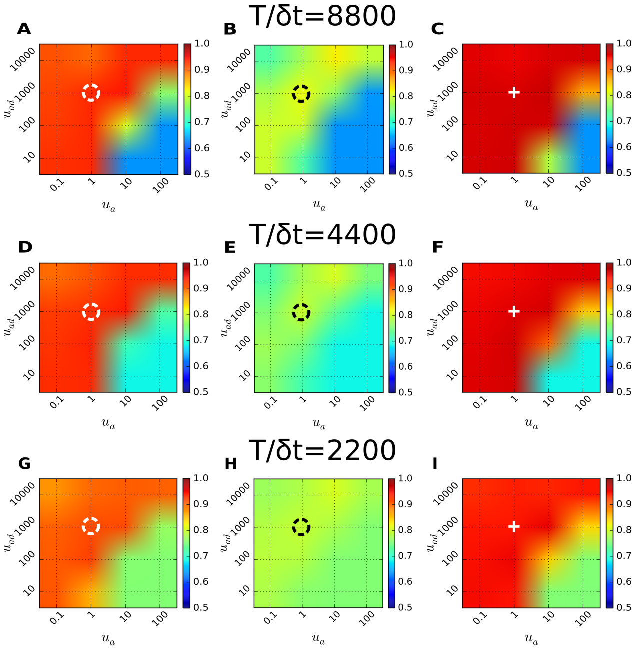

S22 Fig: Accuracy of the model prediction in terms of $\langle\chi\rangle$ under varying prior parameters u_a, and u_{ad}. To calculate the diagram 100 time traces were analyzed at each condition. $\langle\chi\rangle$ of each graph was evaluated for the data generated with the fixed parameters $\gamma^{(1)\to(2)}\Delta t = \gamma^{(2)\to(2)}\Delta t = 0.001, k^{(1)}_{L\to H}\Delta t = k^{(1)}_{H\to L}\Delta t = 0.05$, and (A) $k^{(2)}_{L\to H}\Delta t = 0.00625, k^{(2)}_{H\to L}\Delta t = 0.0125$, $T_{obs}/\Delta t$= 8800. (B) $k^{(2)}_{L\to H}\Delta t = 0.025, k^{(2)}_{H\to L}\Delta t = 0.1$, $T_{obs}/\Delta t$= 8800. (C) $k^{(2)}_{L\to H}\Delta t = 0.1, k^{(2)}_{H\to L}\Delta t = 0.2$, $T_{obs}/\Delta t$= 8800. (D-F) Identical conditions with (A-C) except $T_{obs}/\Delta t$= 4400. (G-I) Identical conditions with (A-C) except $T_{obs}/\Delta t$= 2200. Black and white circles, and white cross in figure indicate the standard choice of $u_a = 1$ and $u_{ad} = 1000$ used for other analyses.

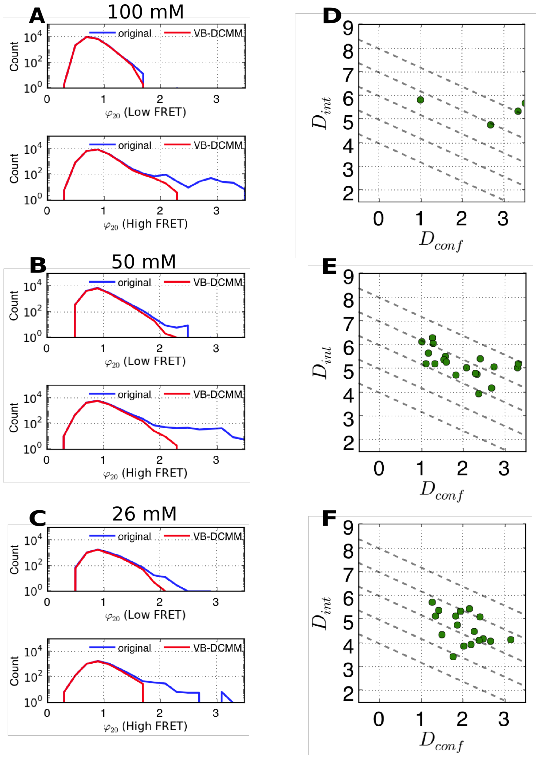

S23 Fig: Effect of decomposing the original H-DNA time traces into its homogeneous Markov components. (A) (Top): Comparison of $\varphi_{20} = \sigma_{20}/\mu_{20}$ histograms from low FRET dwell time data before (blue) and after removing dynamic disorder (red) by VB-DCMM. (Bottom): Comparison of $\varphi_{20} = \sigma_{20}/\mu_{20}$ histograms for high FRET dwell time data. $[Na^+]$=100 mM data were used. Same analyses for $[Na^+]$=50 mM and $[Na^+]$=26 mM are shown in (B) and (C) respectively. (D-F) $D_{\rm conf}$ and $D_{\rm int}$ of (D) $[Na^+]$=100 mM data, (E) $[Na^+]$=50 mM data, and (F) $[Na^+]$=26 mM data. Each data point denotes the values of $D_{\rm conf}$ and $D_{\rm int}$ of individual time traces. Only the time traces exhibiting more than three transition events between internal states are depicted.

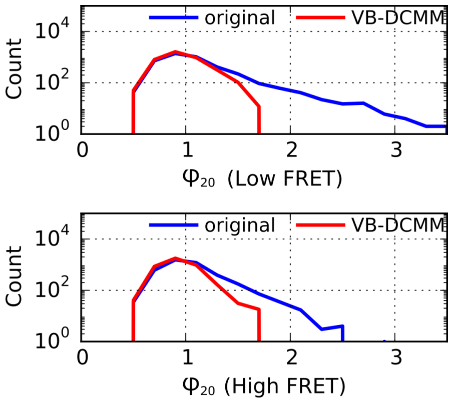

S24 Fig: Comparison of $\varphi_{20} = \sigma_{20}/\mu_{20}$ histograms on synthetic data before and after removing dynamic heterogeneity (red) by decomposing the original traces into the pieces according to estimated internal state trace. The data used in S5 Fig was analyzed. (Top): Comparison of $\varphi_{20} = \sigma_{20}/\mu_{20}$ histograms for low FRET dwell time data. (Bottom): Comparison of $\varphi_{20} = \sigma_{20}/\mu_{20}$ histograms for high FRET dwell time data.

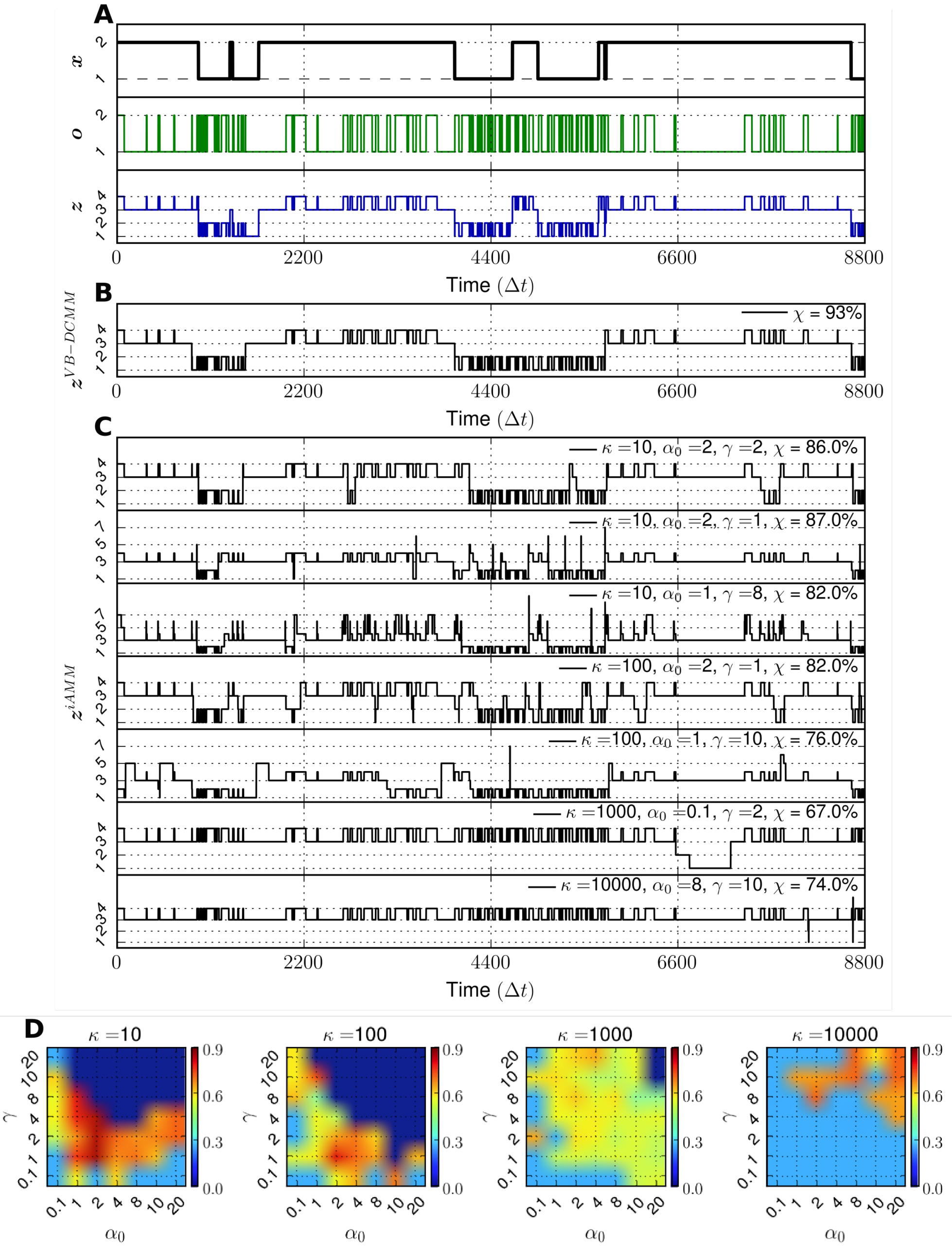

S25 Fig: Comparison between VB-DCMM and sticky-iAMM. (A) Top, middle: Sequence of internal states ($\boldsymbol{x}$) and corresponding observable sequence ($\boldsymbol{o}$) from the same synthetic data from Fig. 3A-(i), (ii). Bottom: Flattened version of $\boldsymbol{x}$ and $\boldsymbol{o}$ ($\boldsymbol{z}$). (B) Estimated $\boldsymbol{z}^{\text{VB-DCMM}}$ using VB-DCMM. $\chi = \frac{1}{T}\sum_{t=1}^{T} \delta_{z(t), z^{\text{model}}(t)} = 0.93$. (C) Examples of estimated $\boldsymbol{z}^{\text{iAMM}}$ using sticky-iAMM (the code in Ref. [12] was used) after 2000 iterations under various prior parameters (κ, α_0, and γ). In all the results from iAMM, χ values are lower than the one obtained from VB-DCMM in (B). (D) Result of sticky-iAMM analysis by varying the prior parameters (κ, α_0, γ) against the time trace ($\boldsymbol{o}$) shown in the middle panel in (A). The values of χ between $\boldsymbol{z}$ and $\boldsymbol{z}^{\text{iAMM}}$ under various conditions are color-coded. When the number of states identified from iAMM is greater than 10, we set $\chi = 0$ because in this case the agreement between $\boldsymbol{z}^{\text{iAMM}}$ and $\boldsymbol{z}$ is practically very low.
