# Supplementary figures and images for: Decoding Single Molecule Time Traces with Dynamic Disorder
Source: PLoS Comput Biol. 2016 Dec 27;12(12):e1005286. doi: 10.1371/journal.pcbi.1005286 (PMC5226833; doi:10.1371/journal.pcbi.1005286)

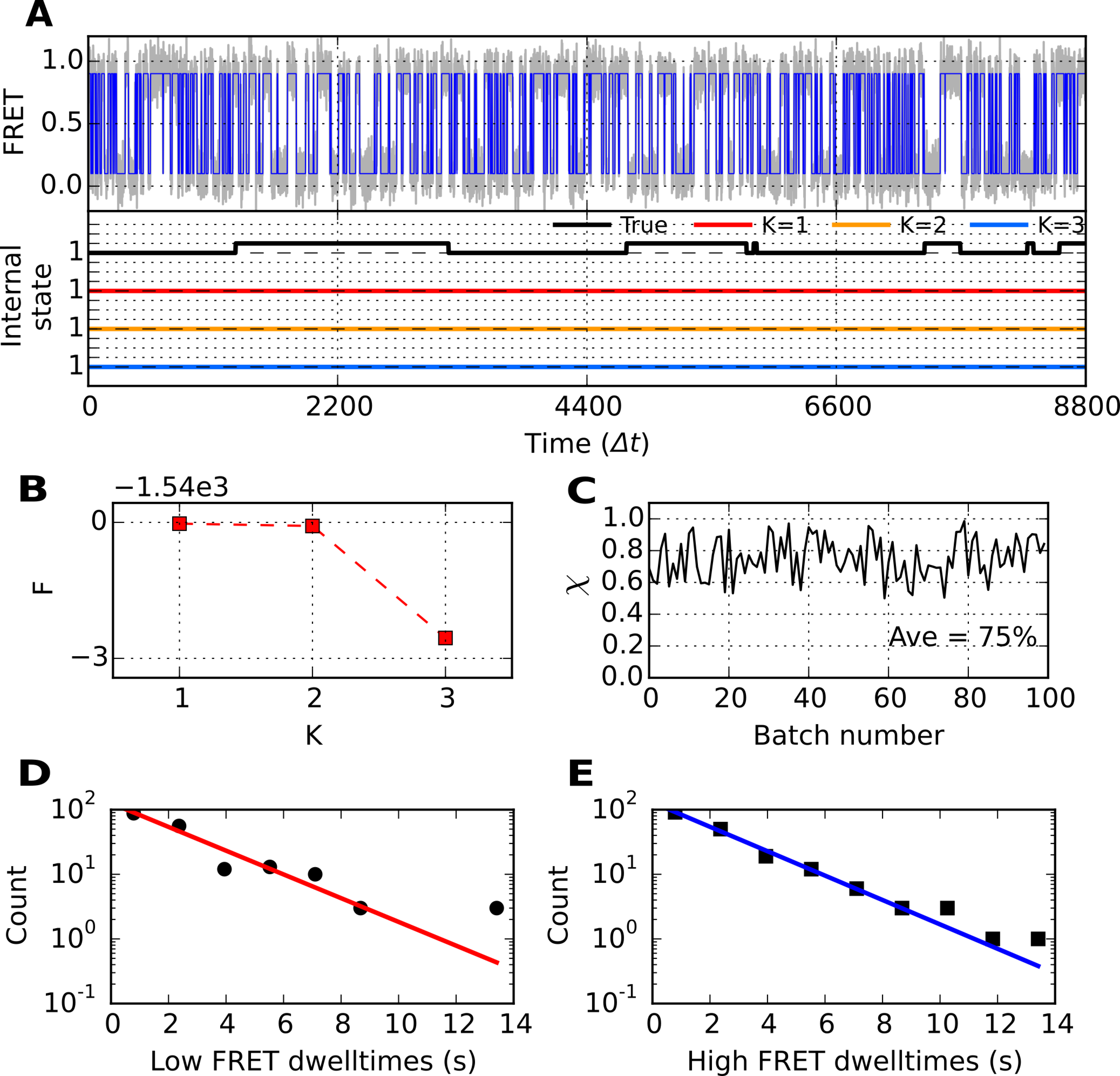

Supplement: S1 Fig — (TIF) [file pcbi.1005286.s002.tif]

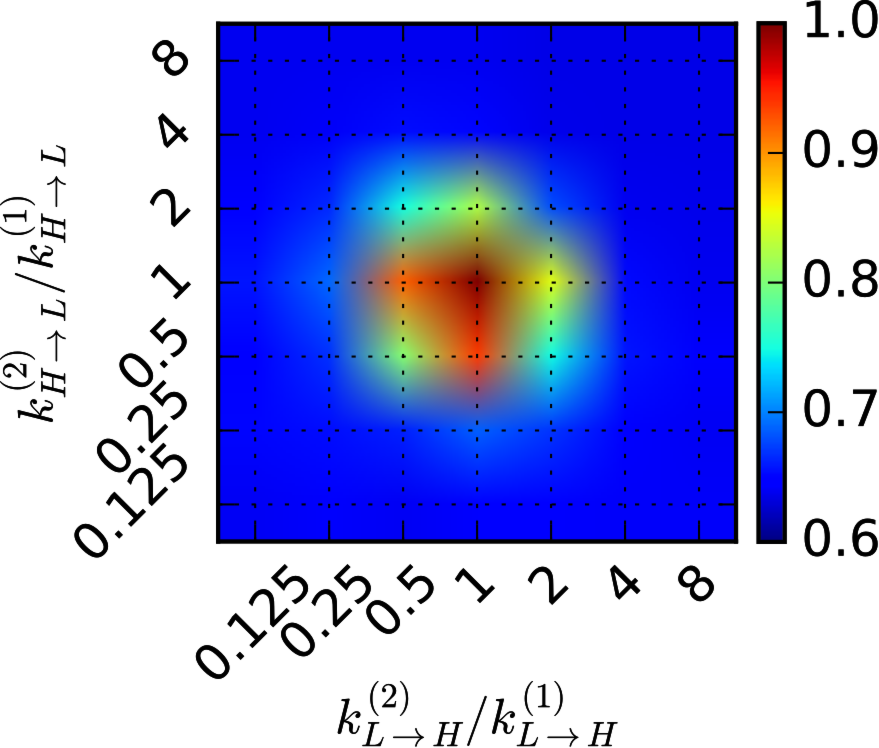

Supplement: S2 Fig — (TIF) [file pcbi.1005286.s003.tif]

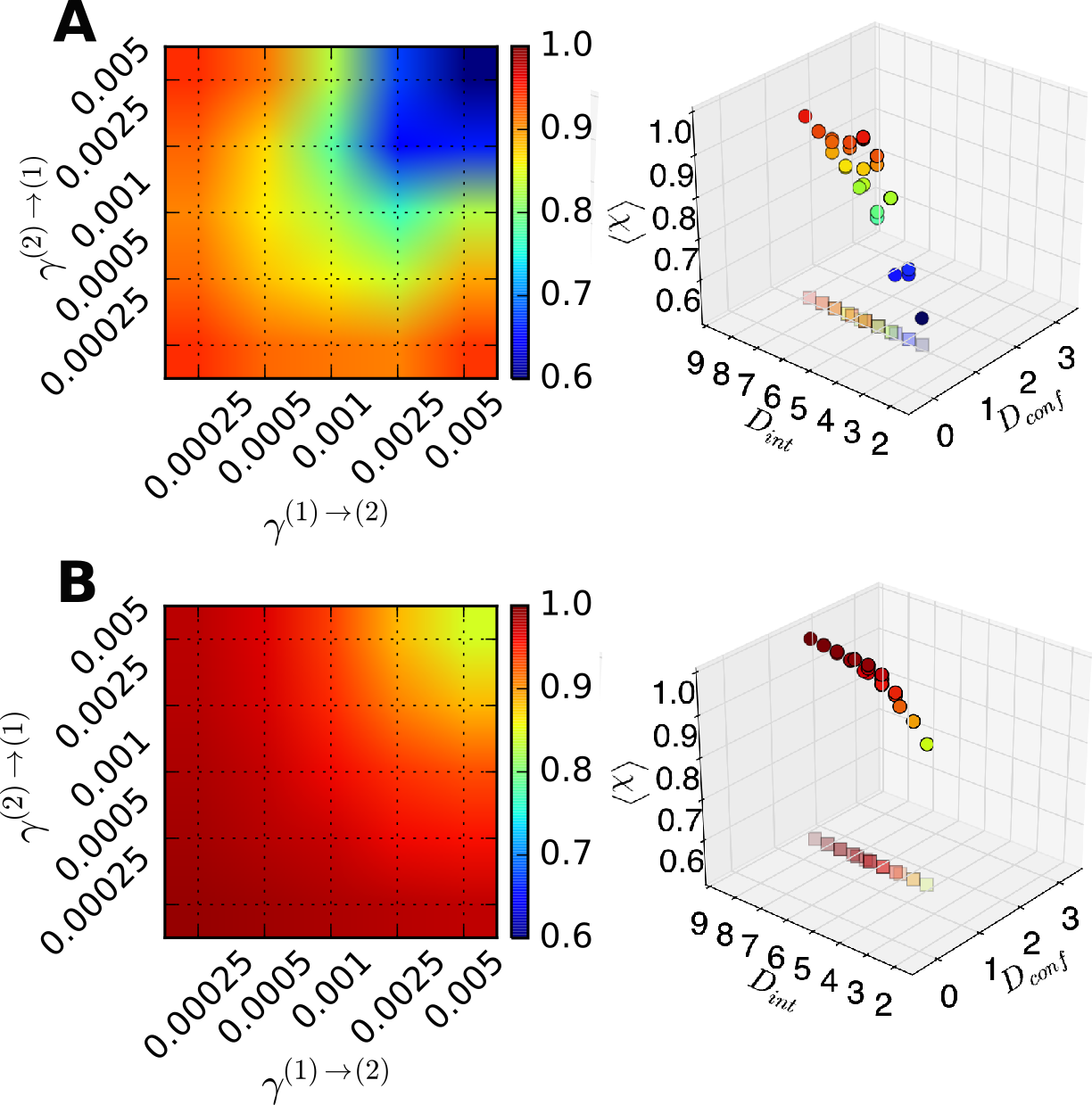

Supplement: S3 Fig — (TIF) [file pcbi.1005286.s004.tif]

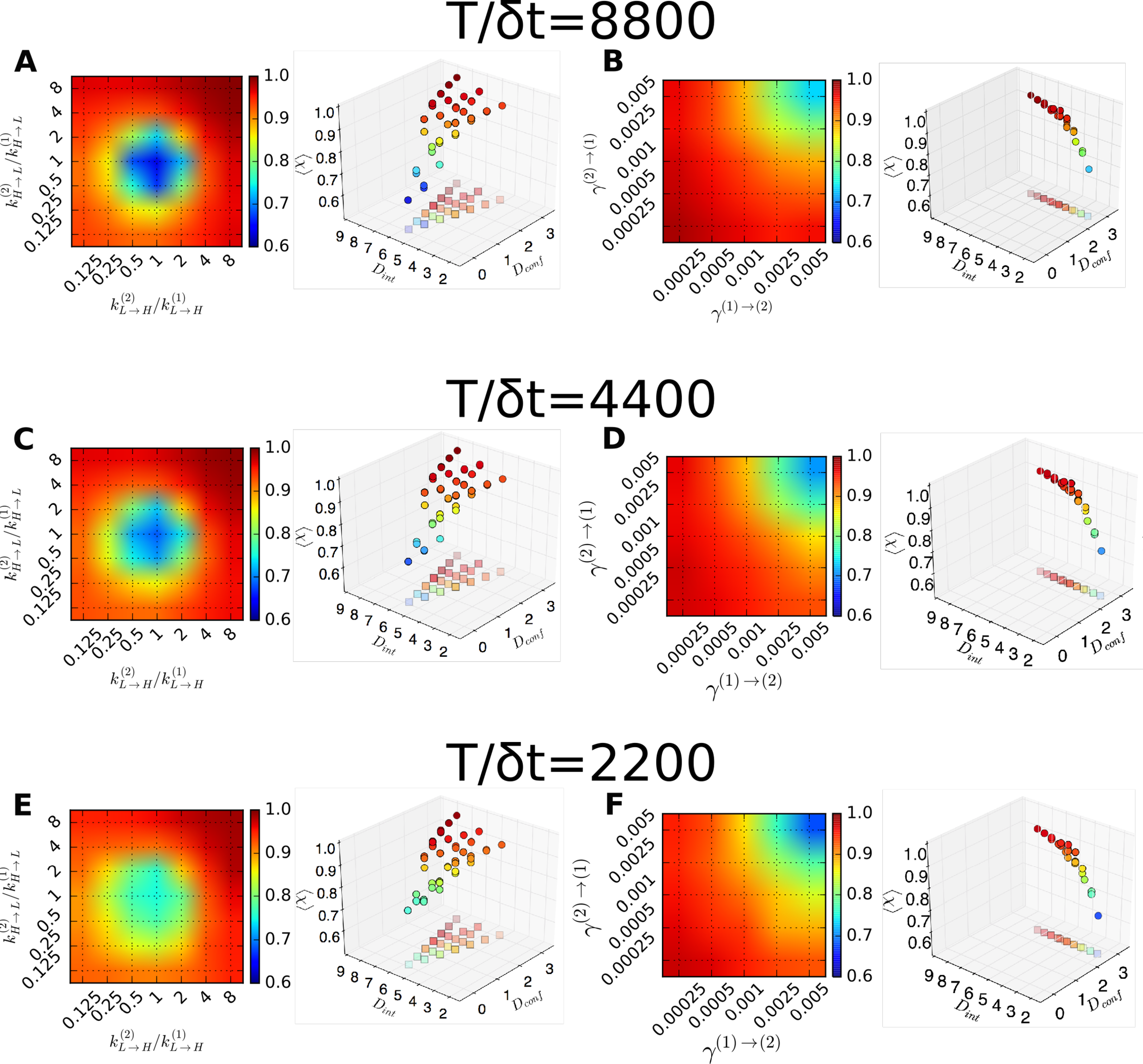

Supplement: S4 Fig — (TIF) [file pcbi.1005286.s005.tif]

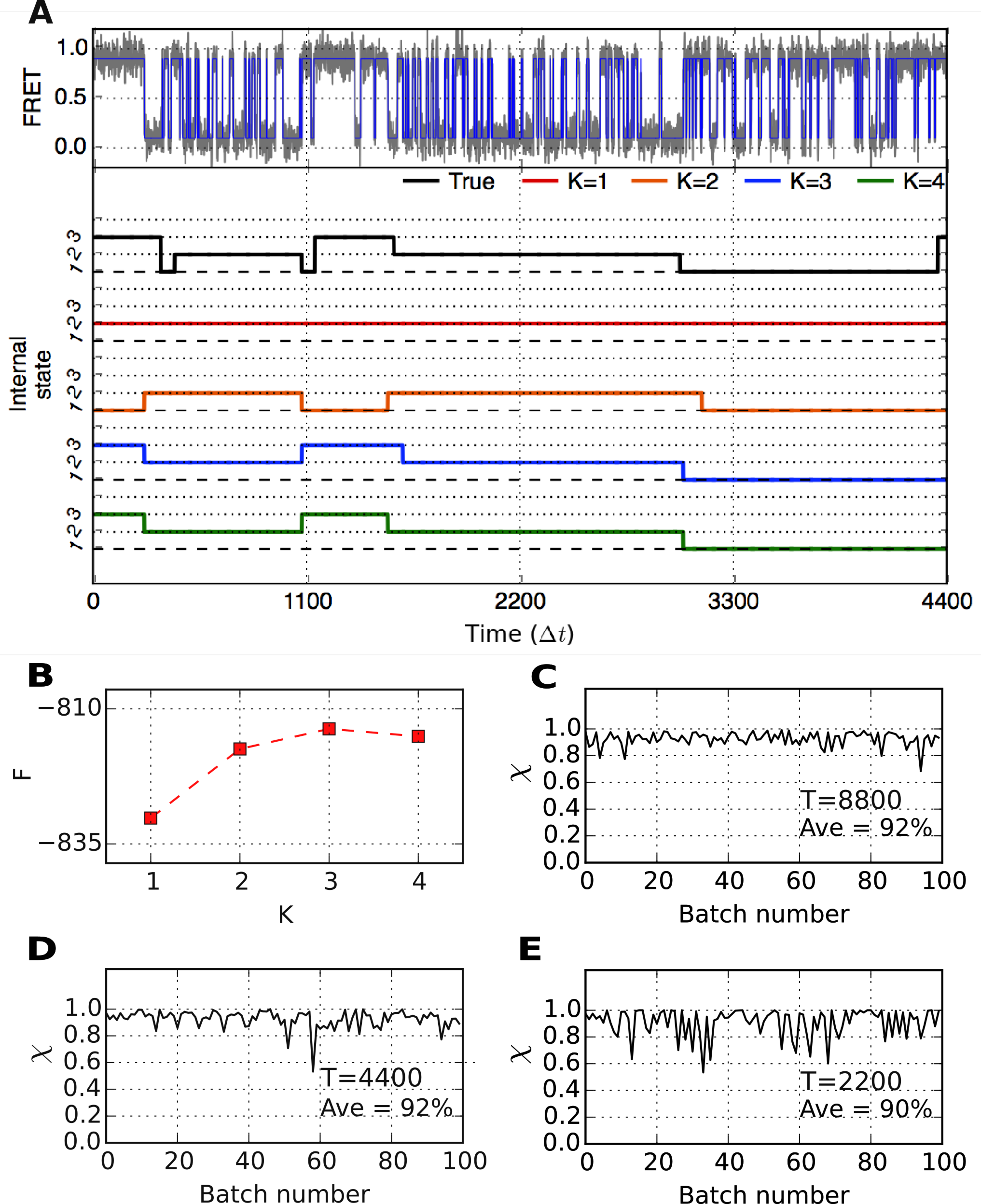

Supplement: S5 Fig — (TIF) [file pcbi.1005286.s006.tif]

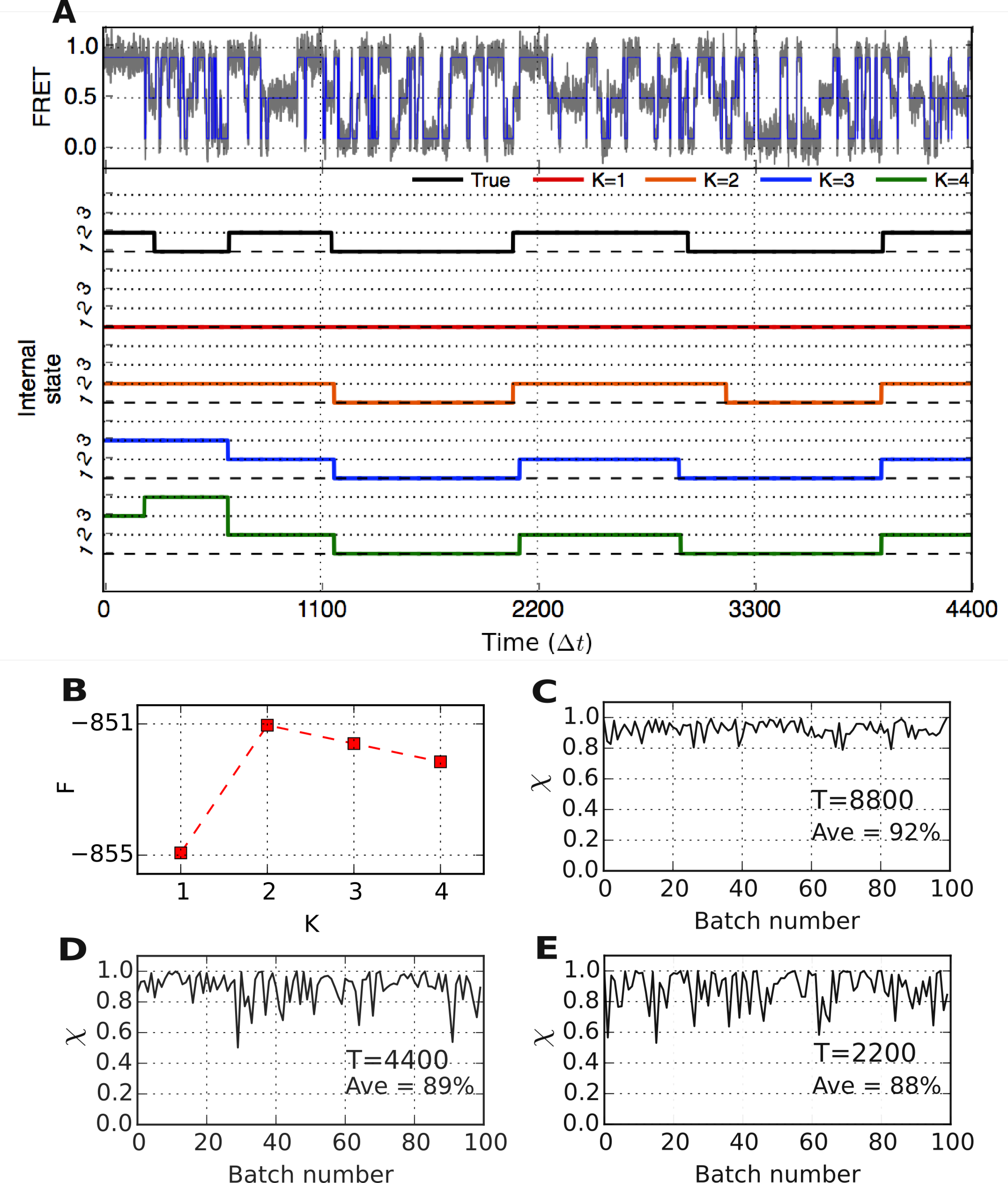

Supplement: S6 Fig — (TIF) [file pcbi.1005286.s007.tif]

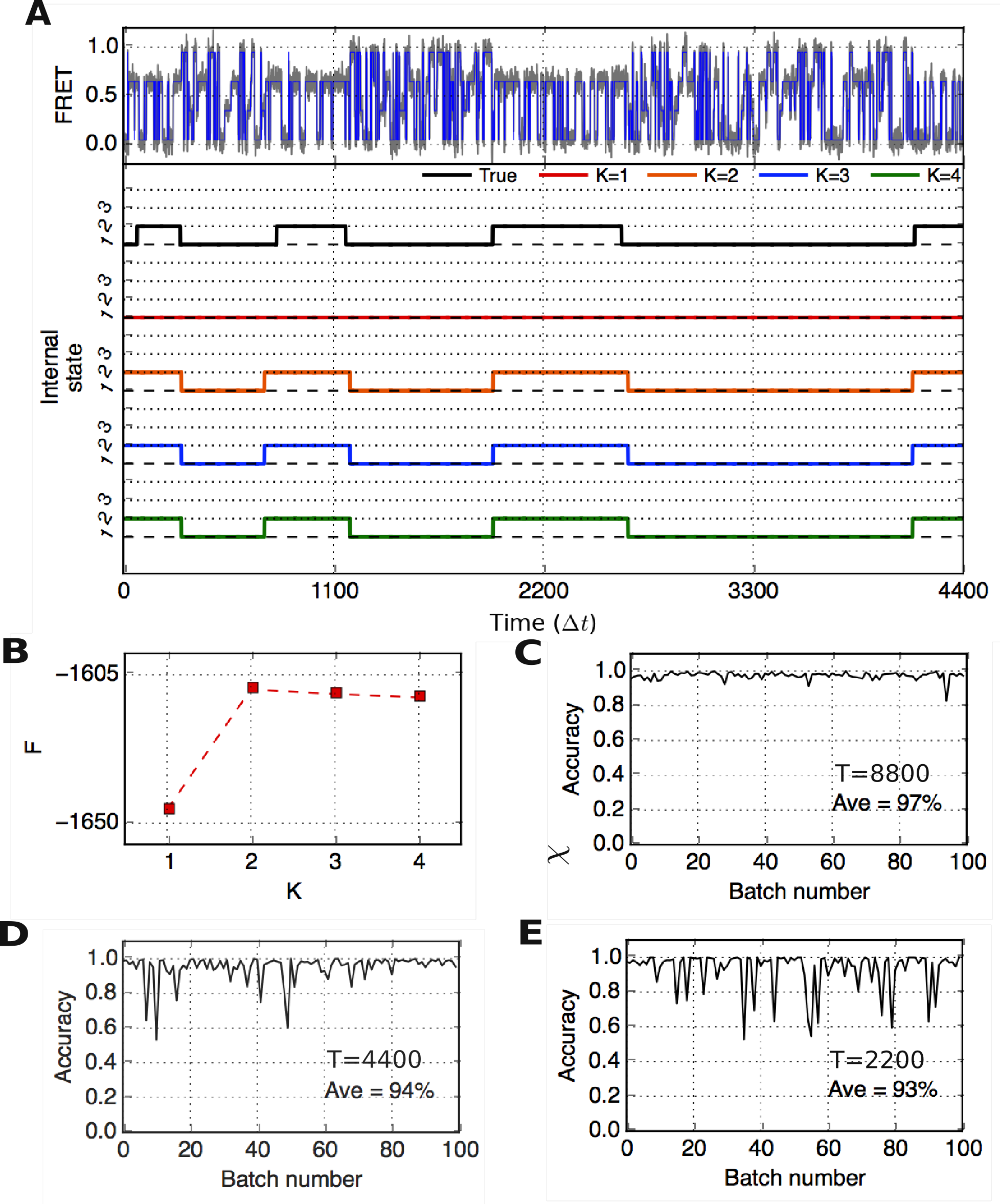

Supplement: S7 Fig — (TIF) [file pcbi.1005286.s008.tif]

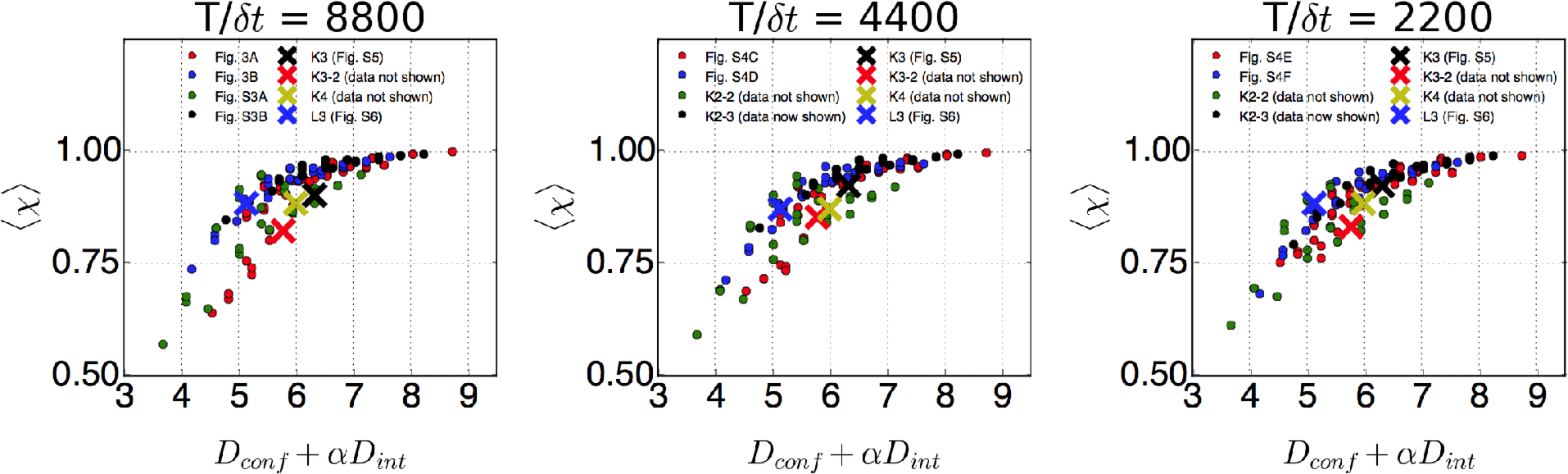

Supplement: S8 Fig — (TIF) [file pcbi.1005286.s009.tif]

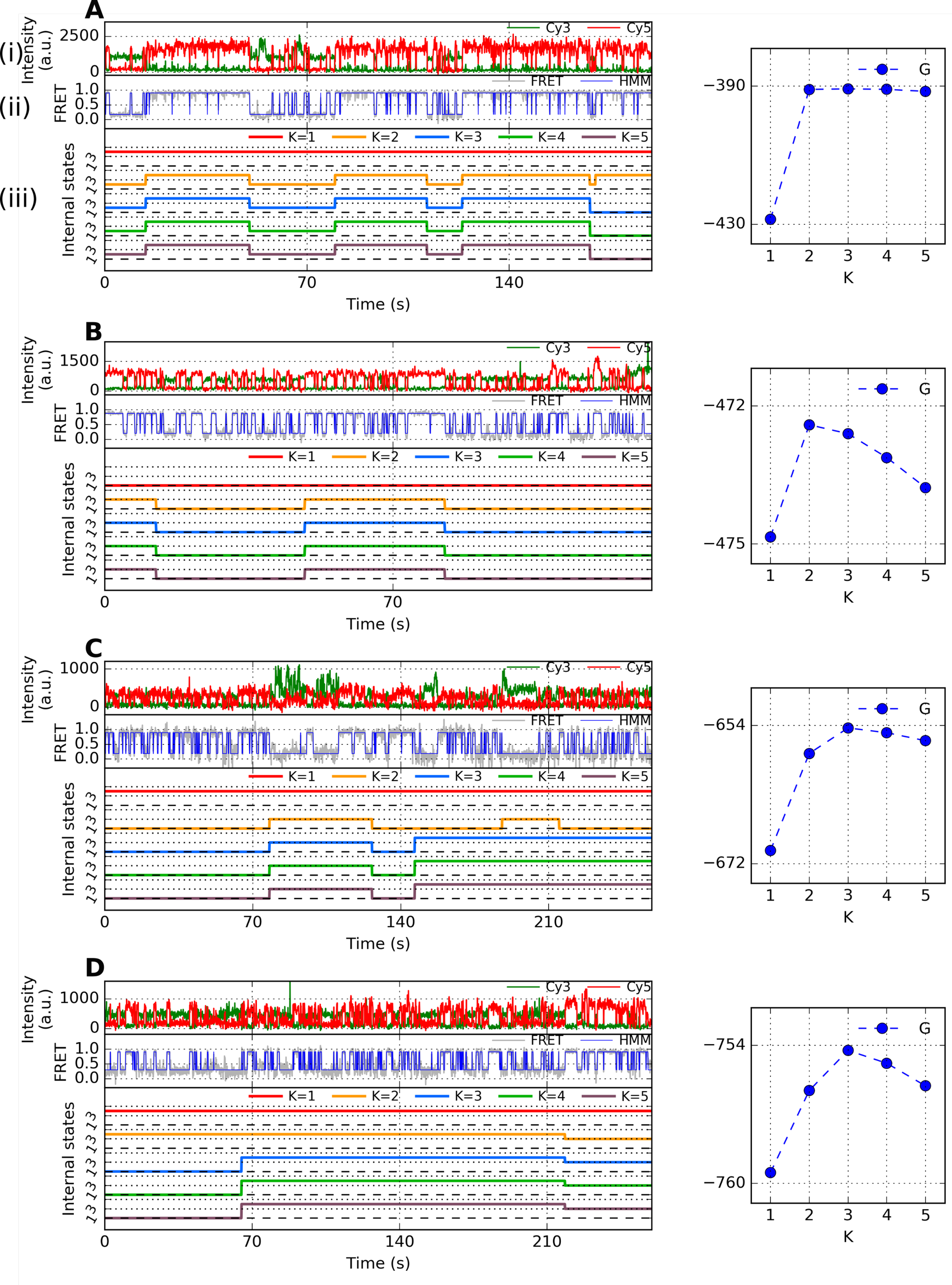

Supplement: S9 Fig — (TIF) [file pcbi.1005286.s010.tif]

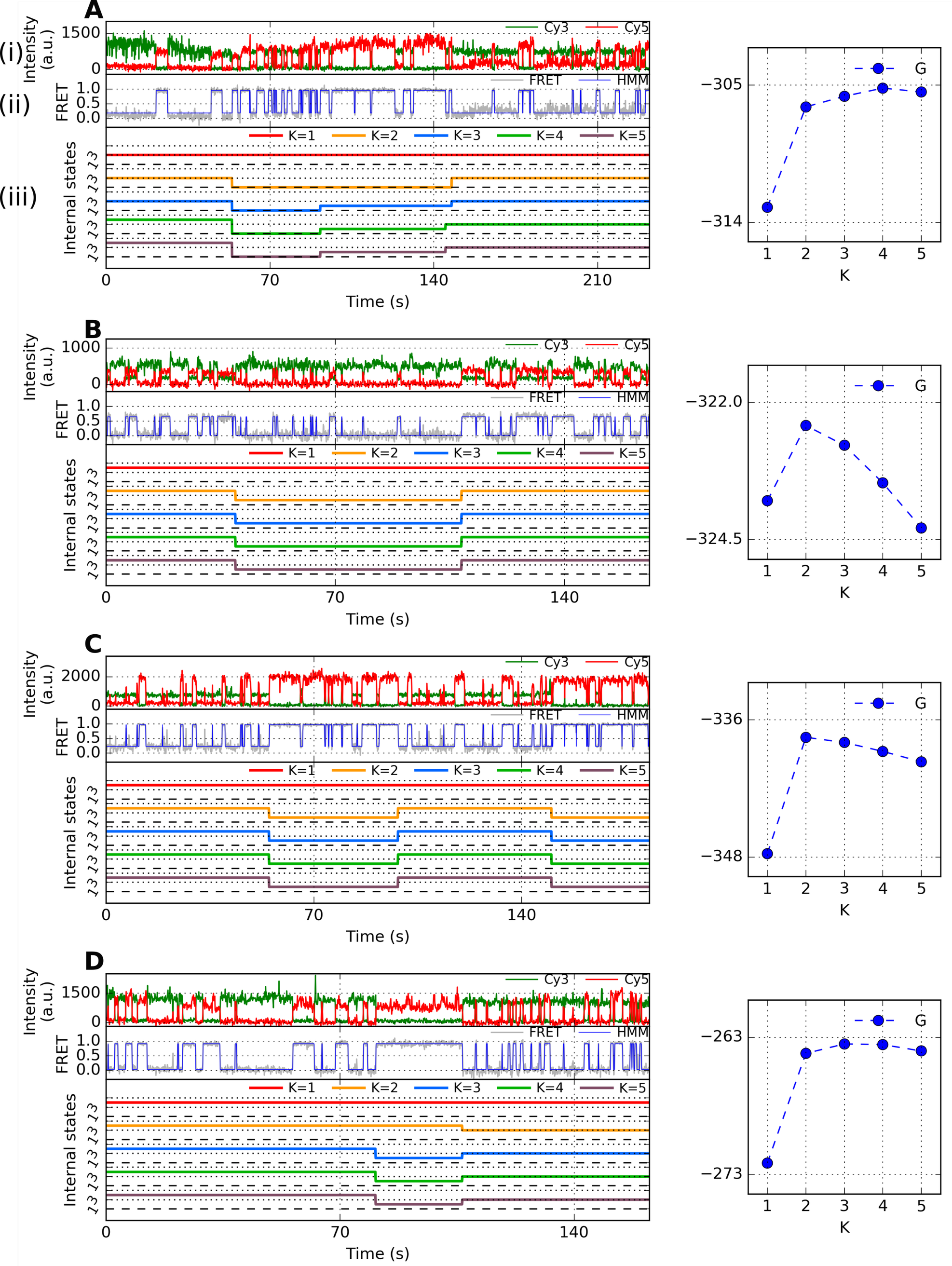

Supplement: S10 Fig — (TIF) [file pcbi.1005286.s011.tif]

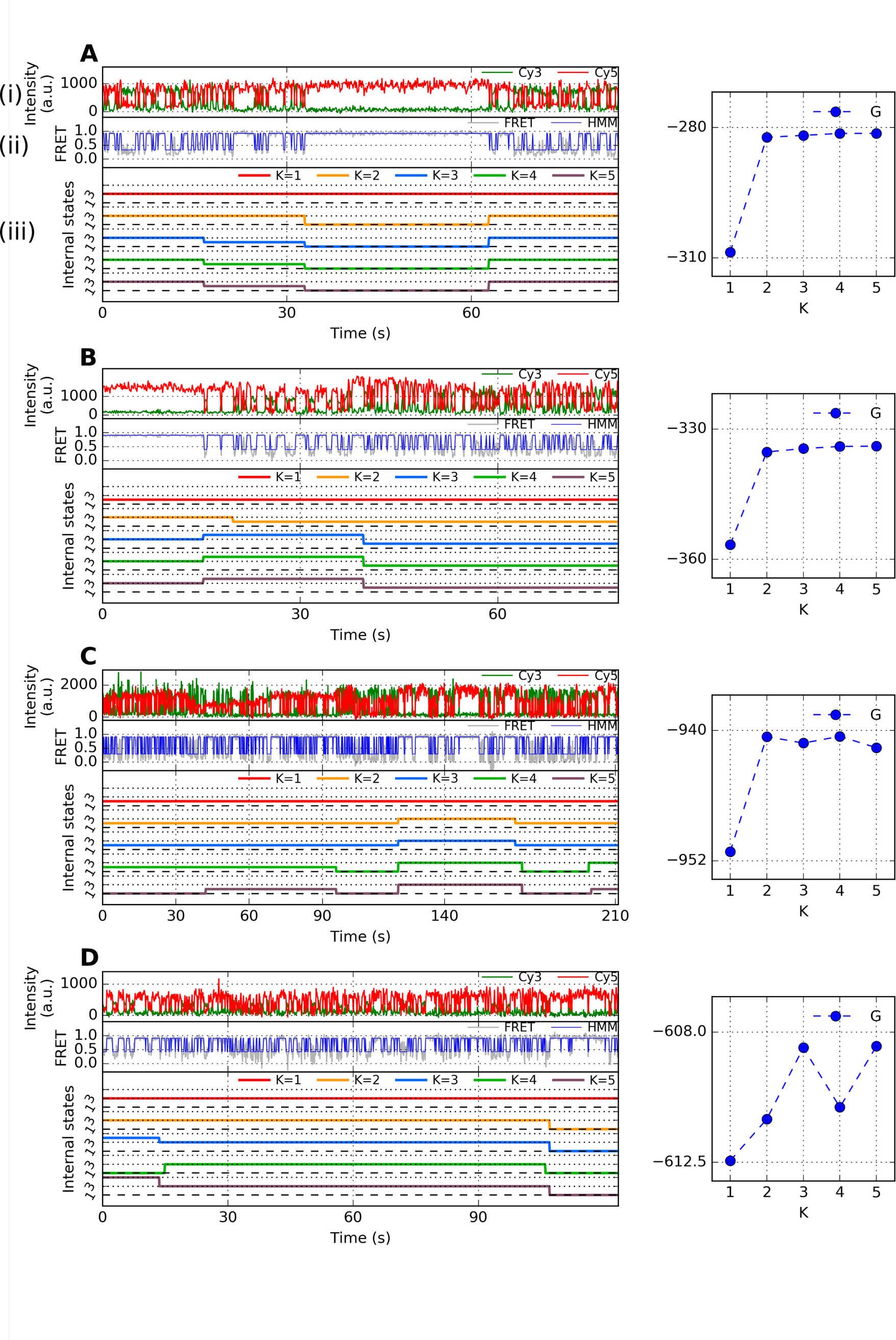

Supplement: S11 Fig — (TIF) [file pcbi.1005286.s012.tif]

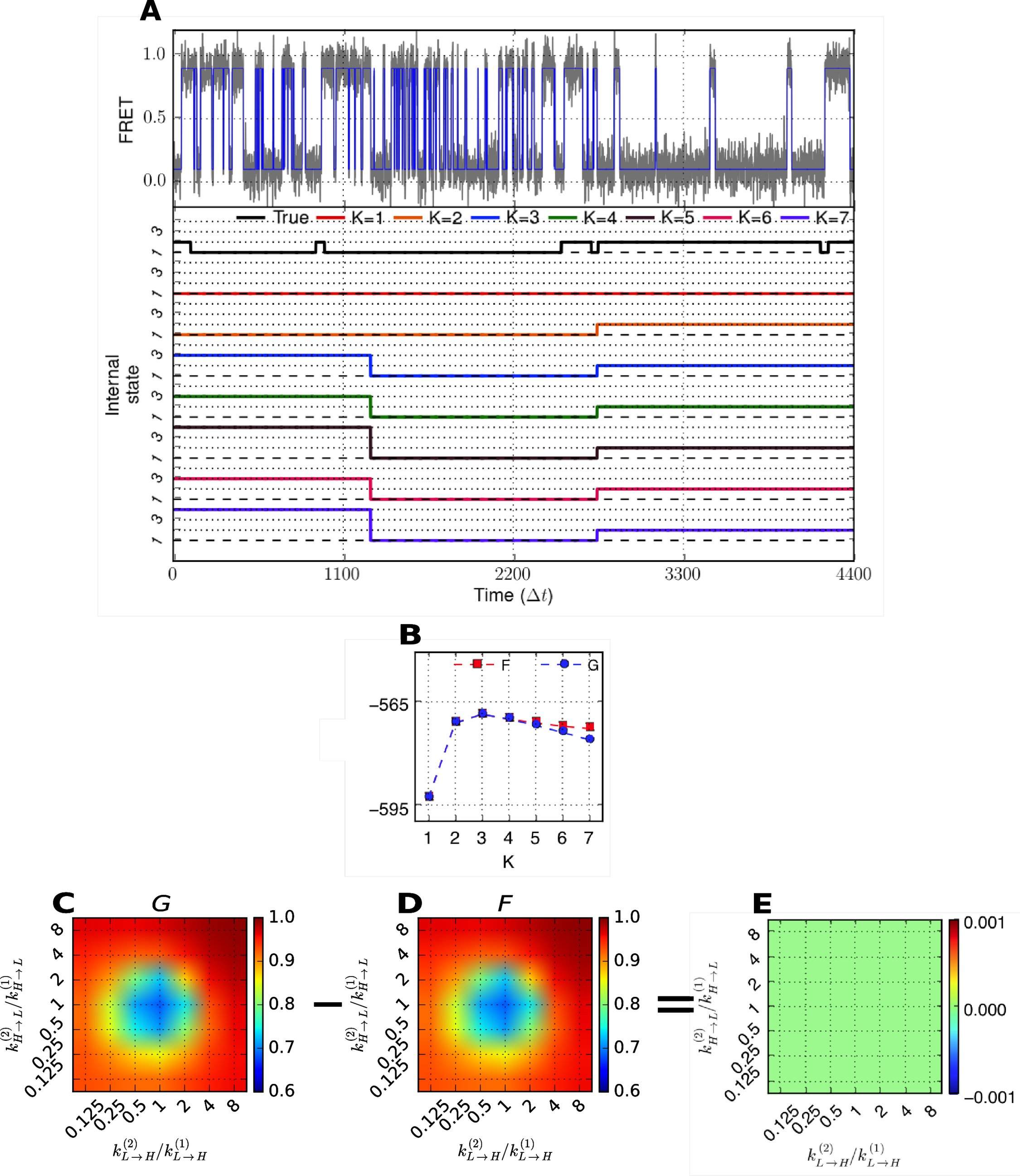

Supplement: S12 Fig — (TIF) [file pcbi.1005286.s013.tif]

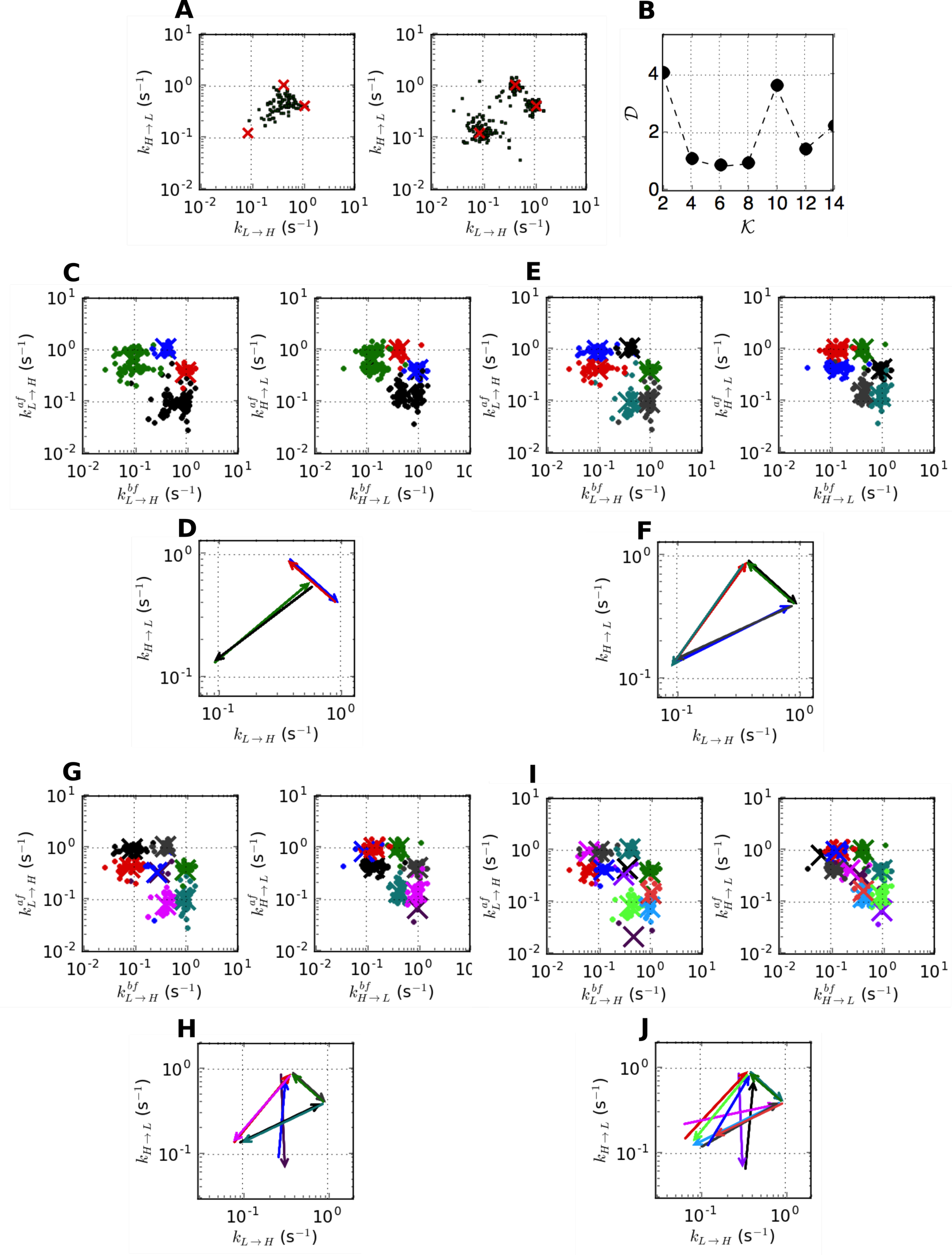

Supplement: S13 Fig — (TIF) [file pcbi.1005286.s014.tif]

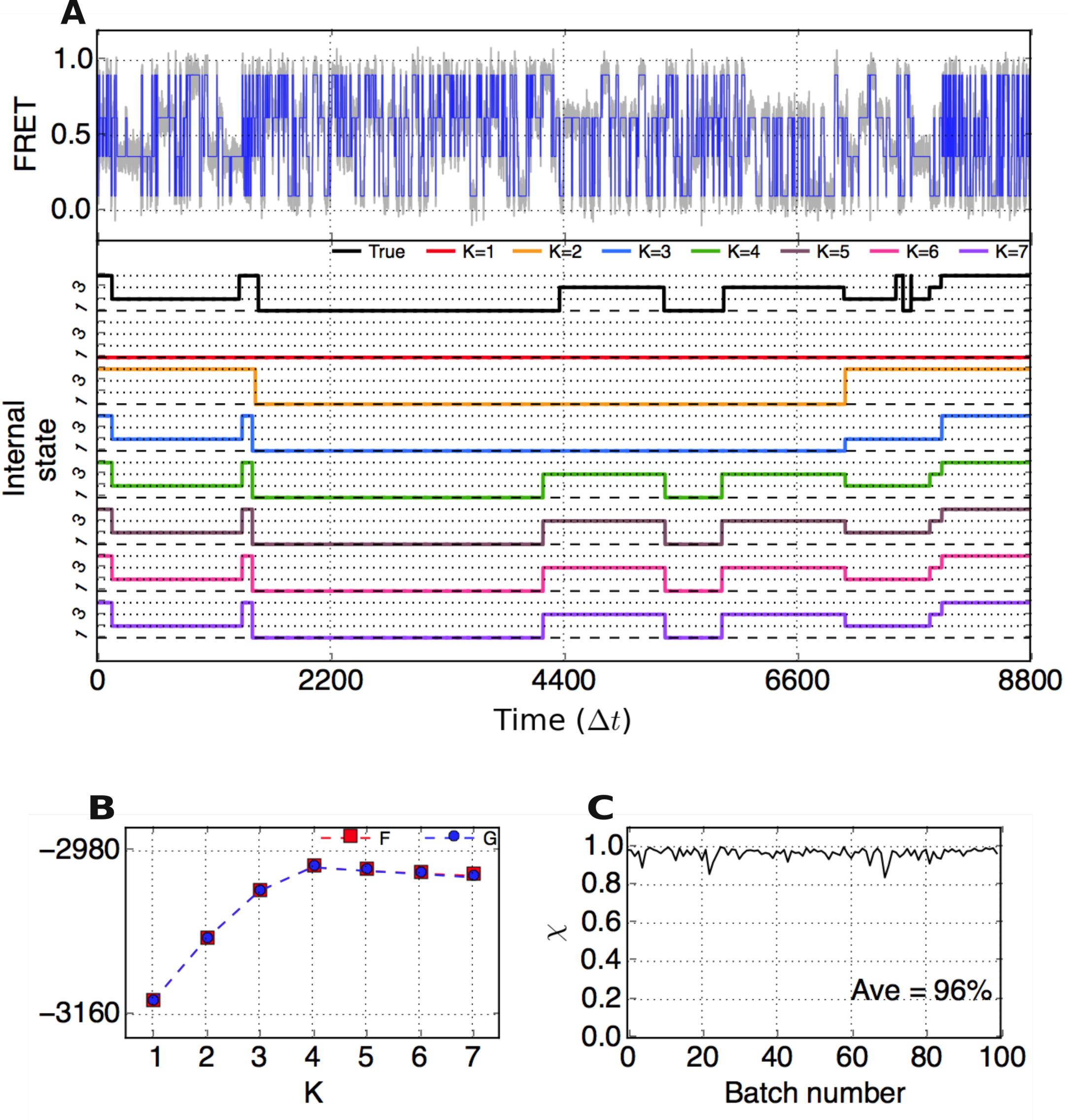

Supplement: S14 Fig — (TIF) [file pcbi.1005286.s015.tif]

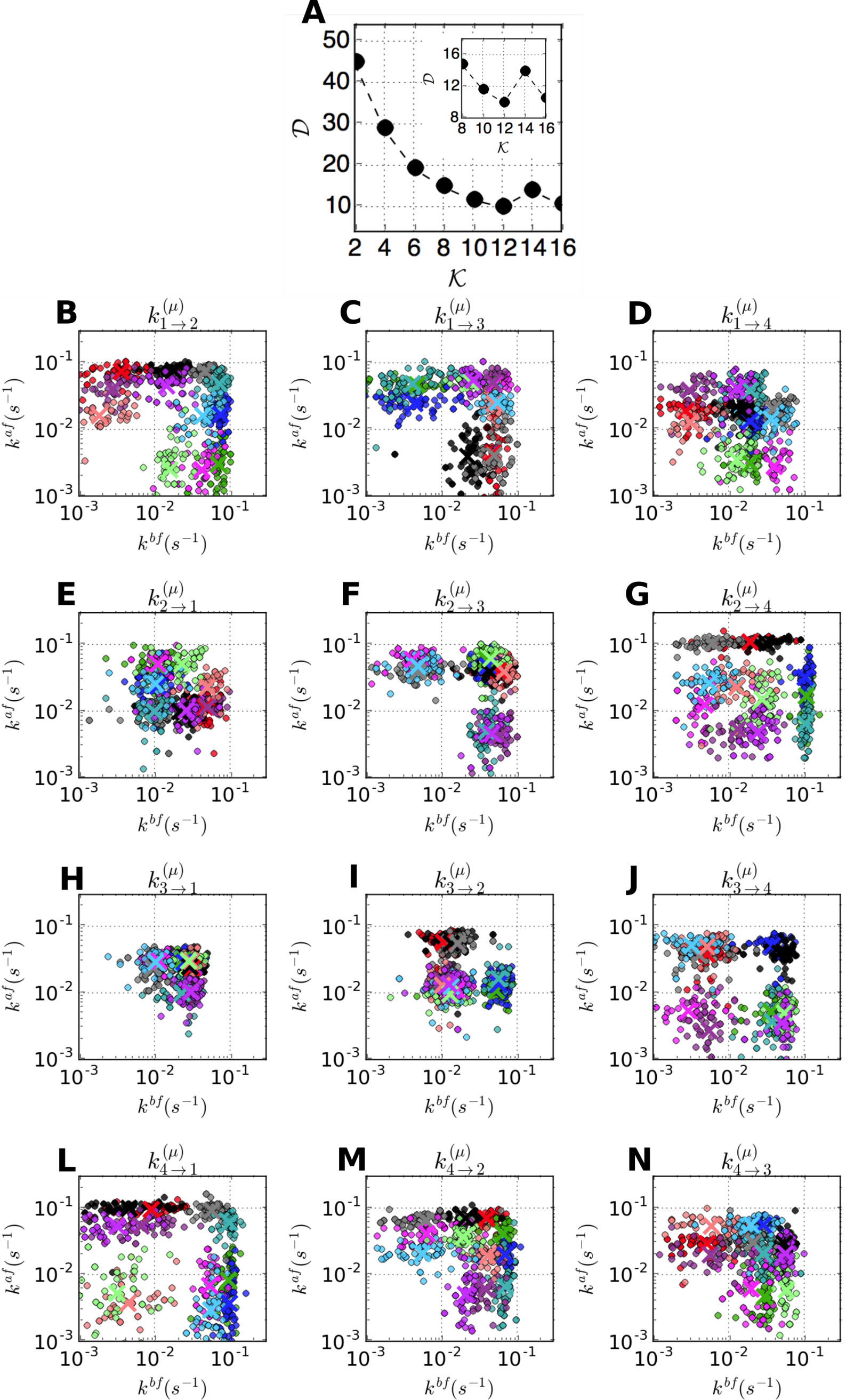

Supplement: S15 Fig — (TIF) [file pcbi.1005286.s016.tif]

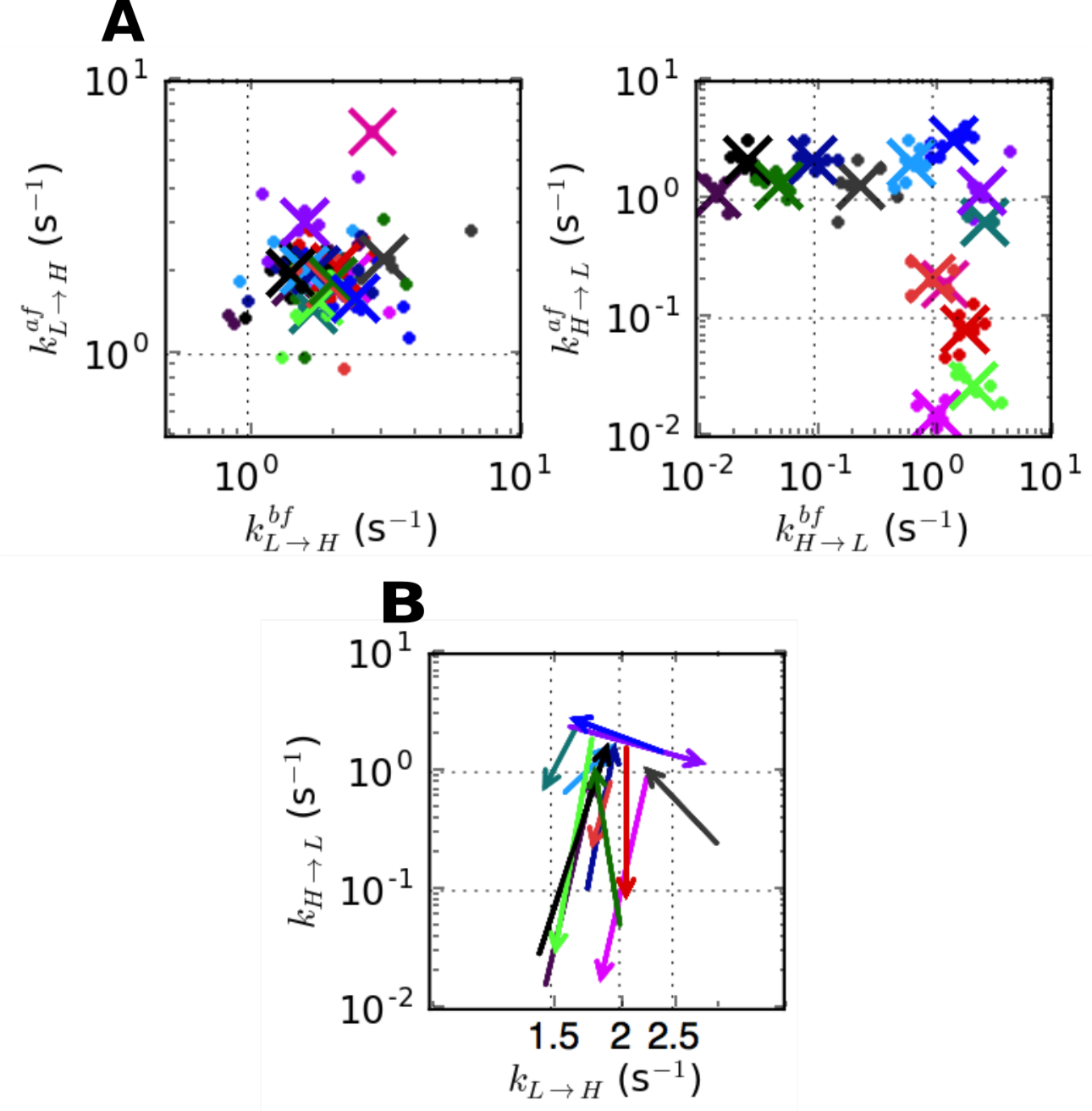

Supplement: S16 Fig — (TIF) [file pcbi.1005286.s017.tif]

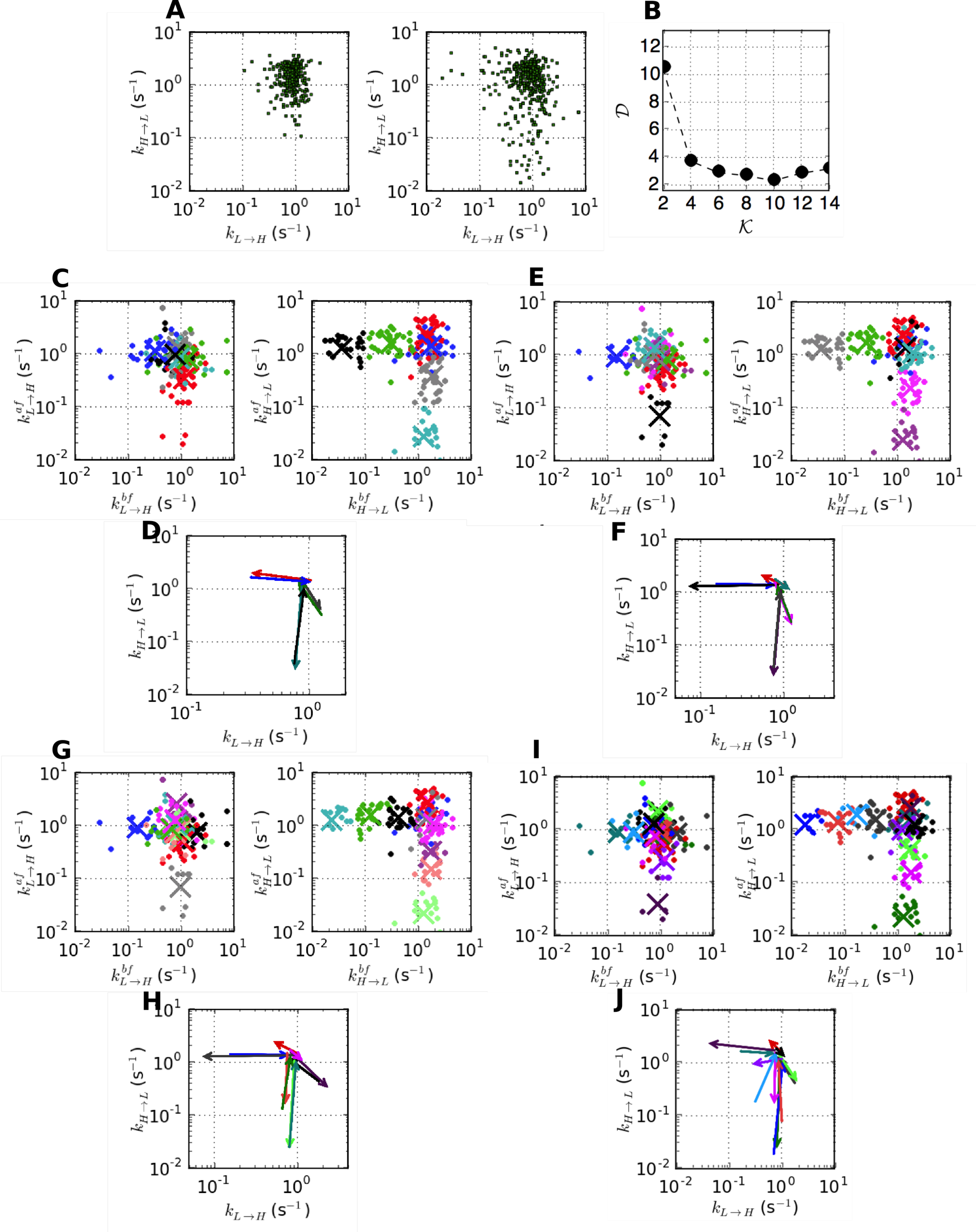

Supplement: S17 Fig — (TIF) [file pcbi.1005286.s018.tif]

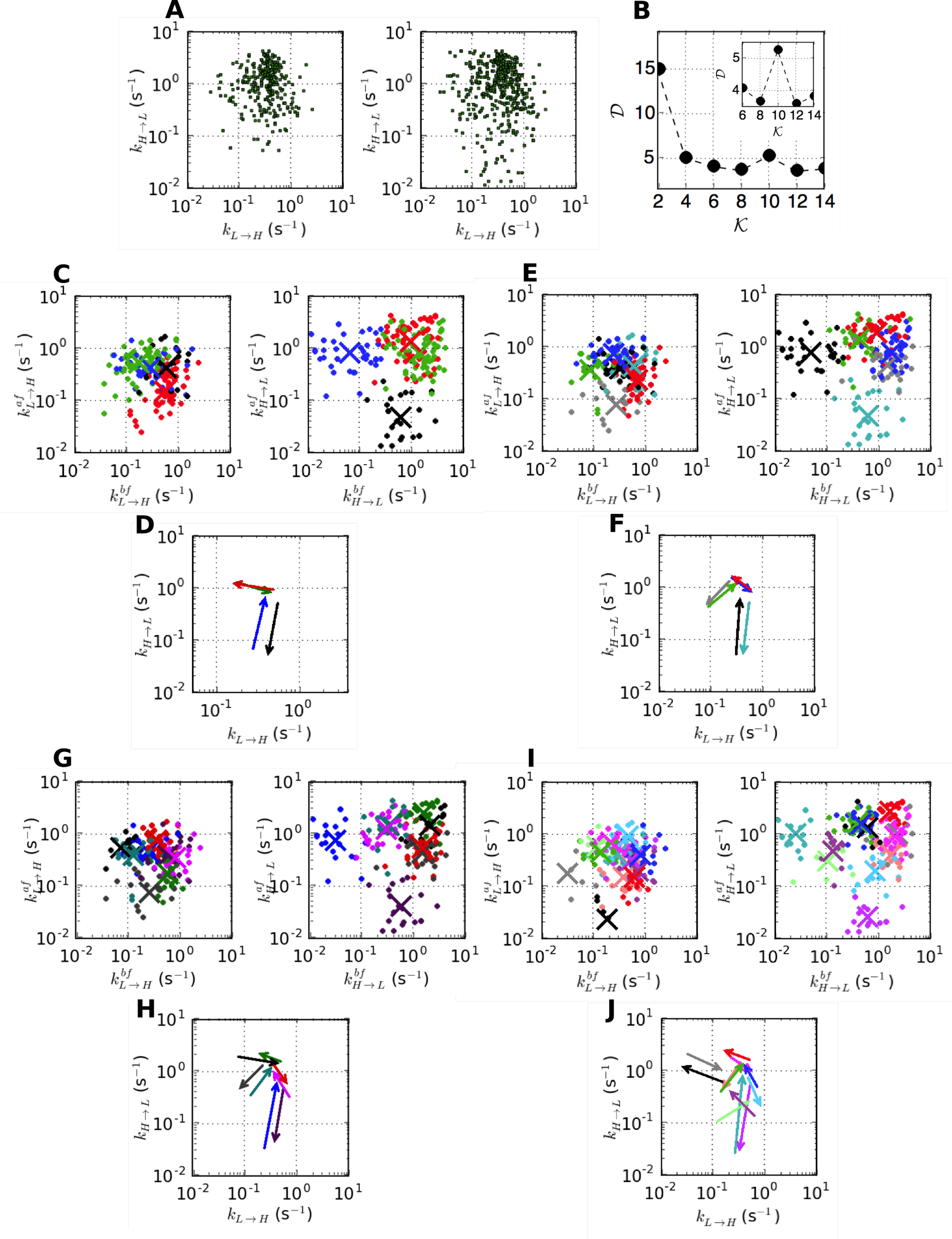

Supplement: S18 Fig — (TIF) [file pcbi.1005286.s019.tif]

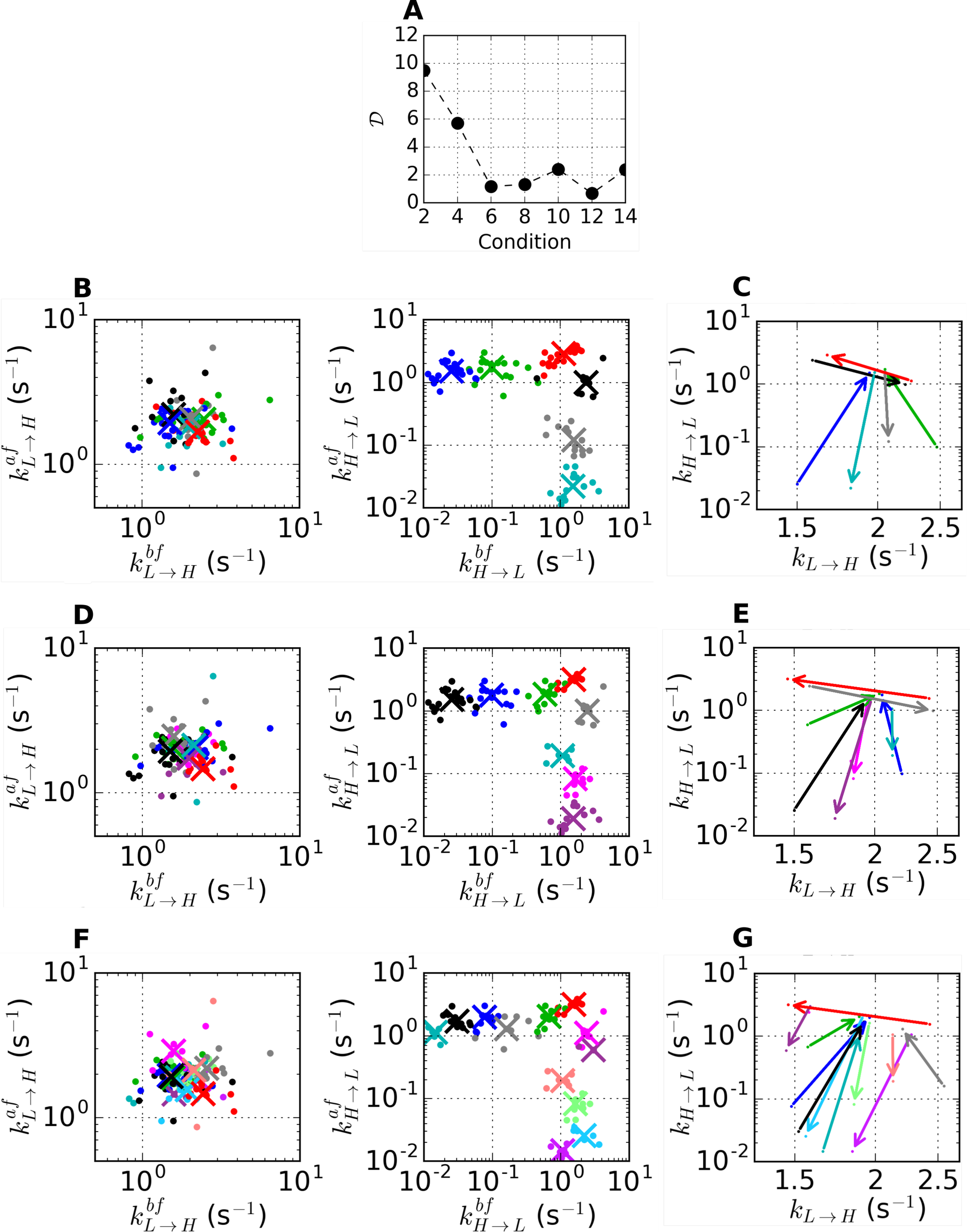

Supplement: S19 Fig — (TIF) [file pcbi.1005286.s020.tif]

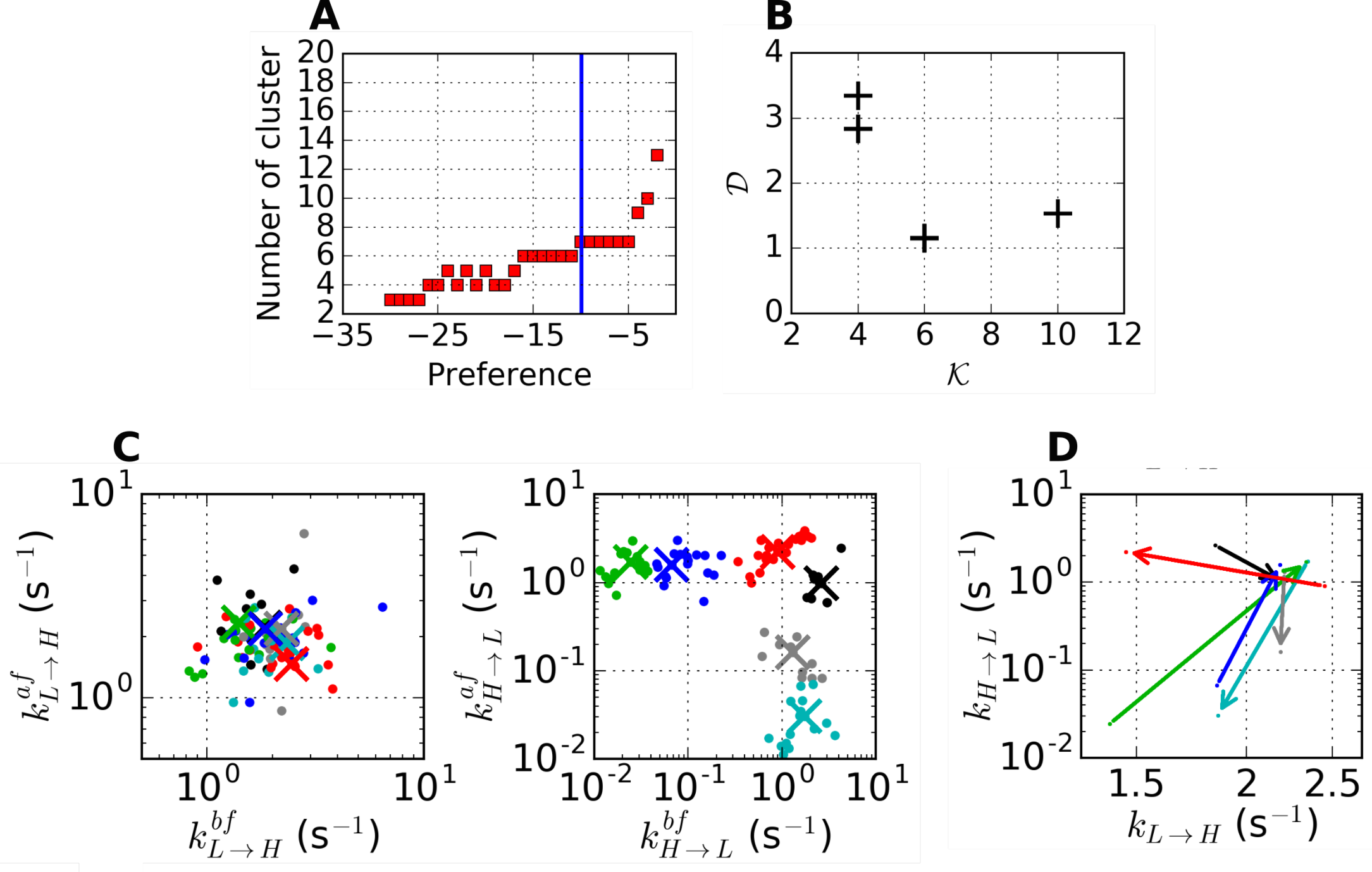

Supplement: S20 Fig — (TIF) [file pcbi.1005286.s021.tif]

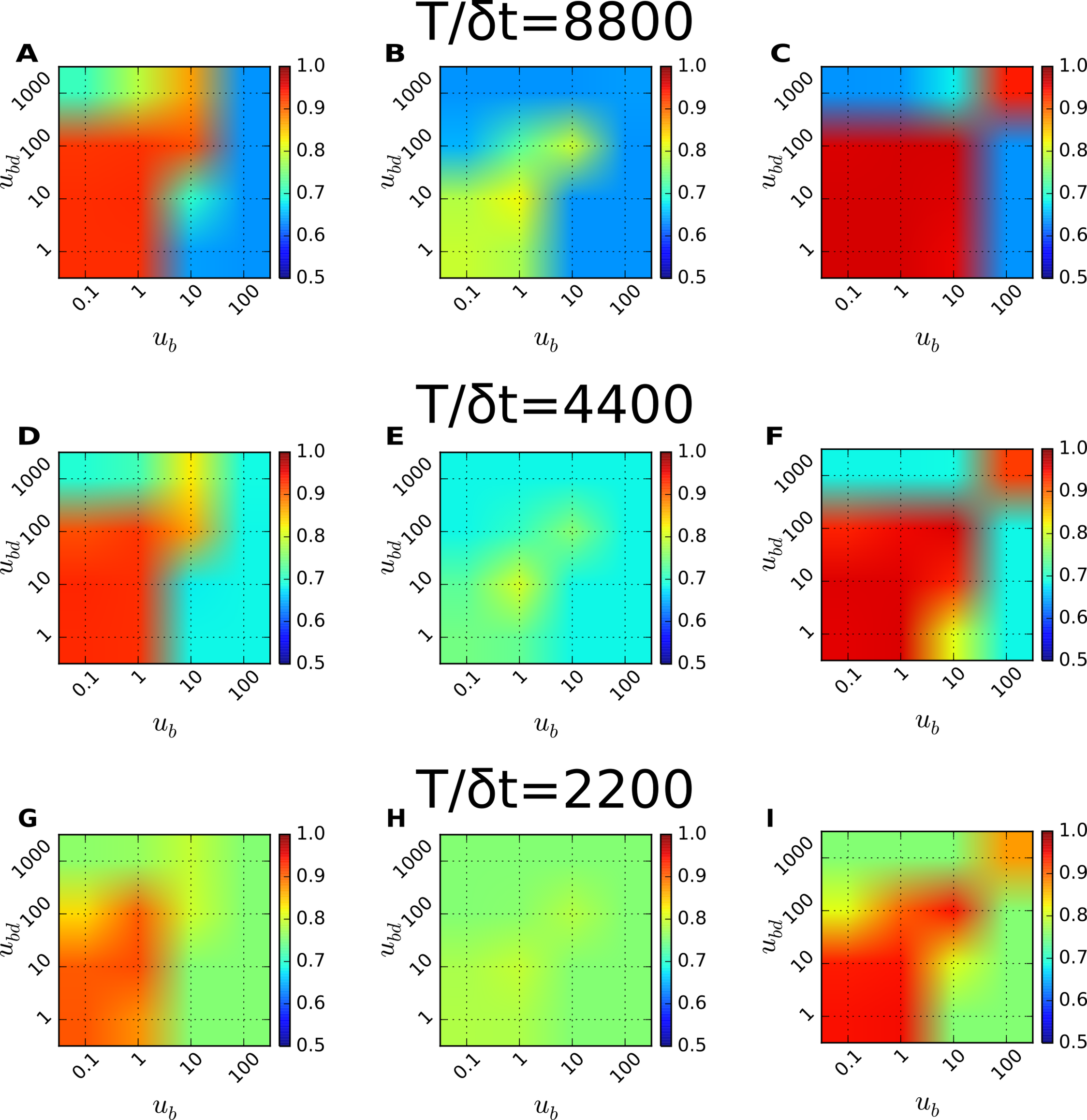

Supplement: S21 Fig — (TIF) [file pcbi.1005286.s022.tif]

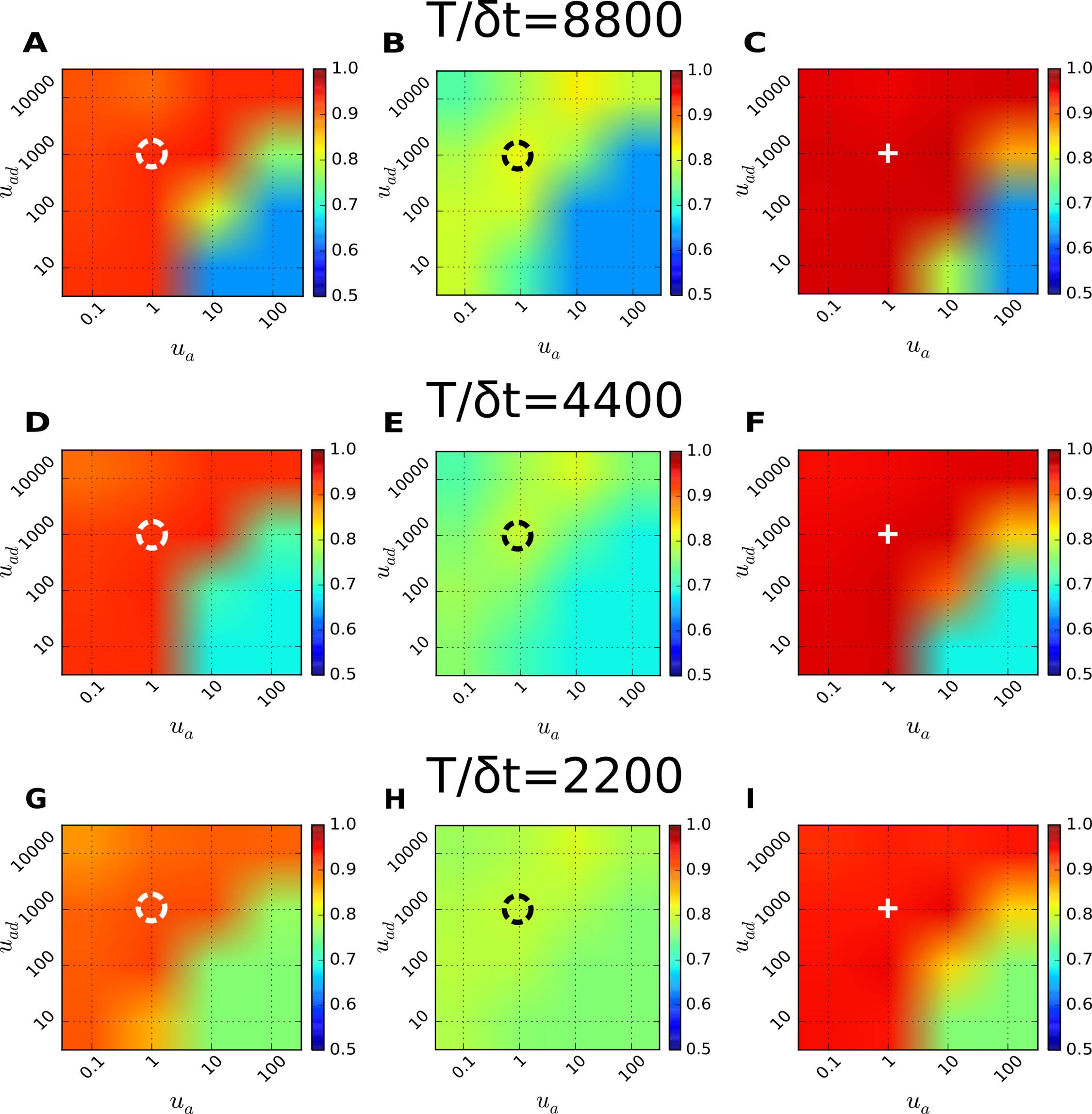

Supplement: S22 Fig — (TIF) [file pcbi.1005286.s023.tif]

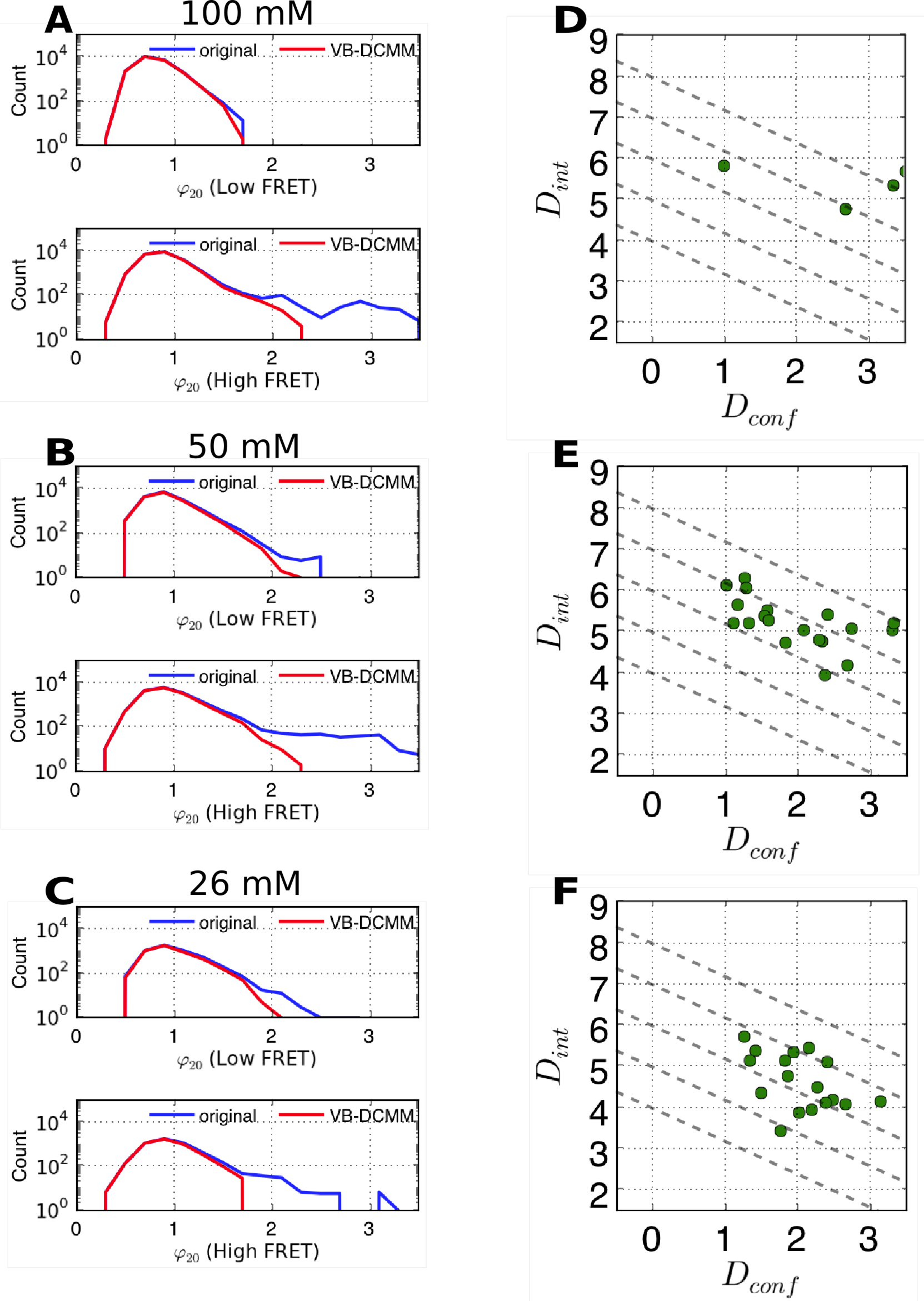

Supplement: S23 Fig — (TIF) [file pcbi.1005286.s024.tif]

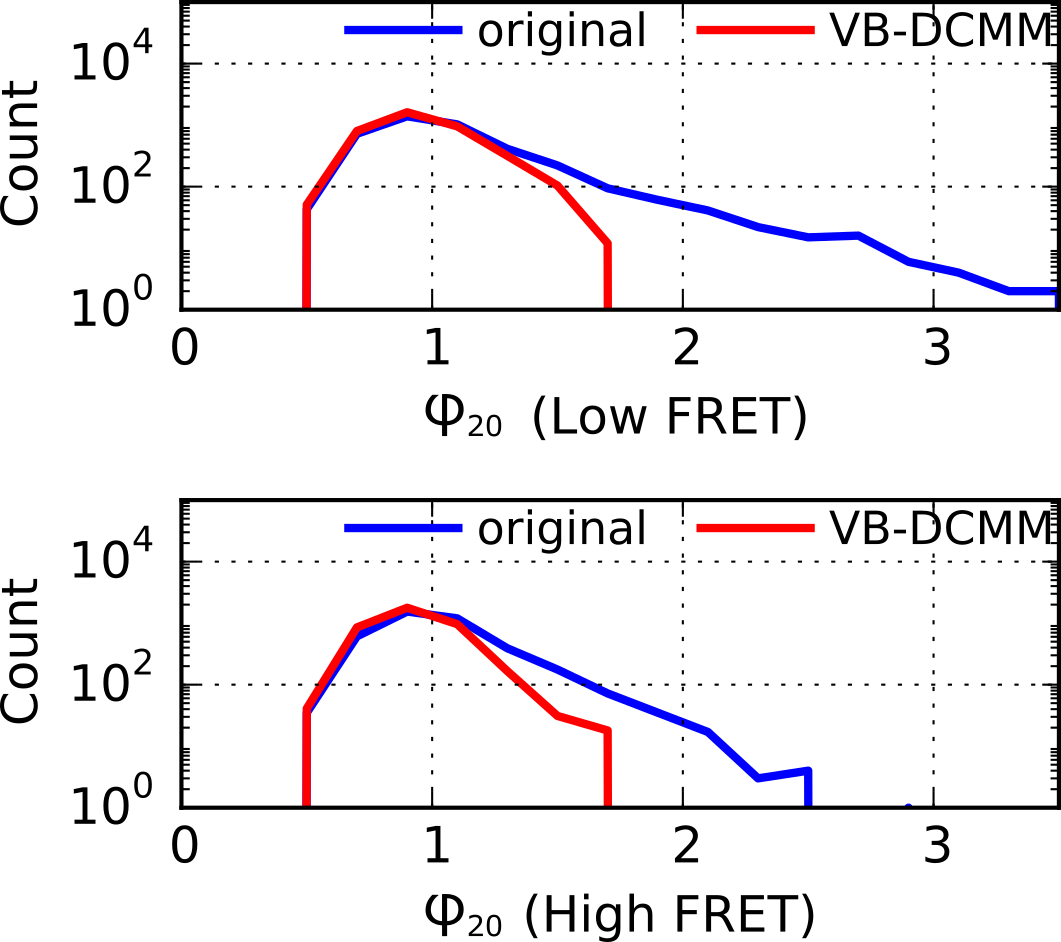

Supplement: S24 Fig — (TIF) [file pcbi.1005286.s025.tif]

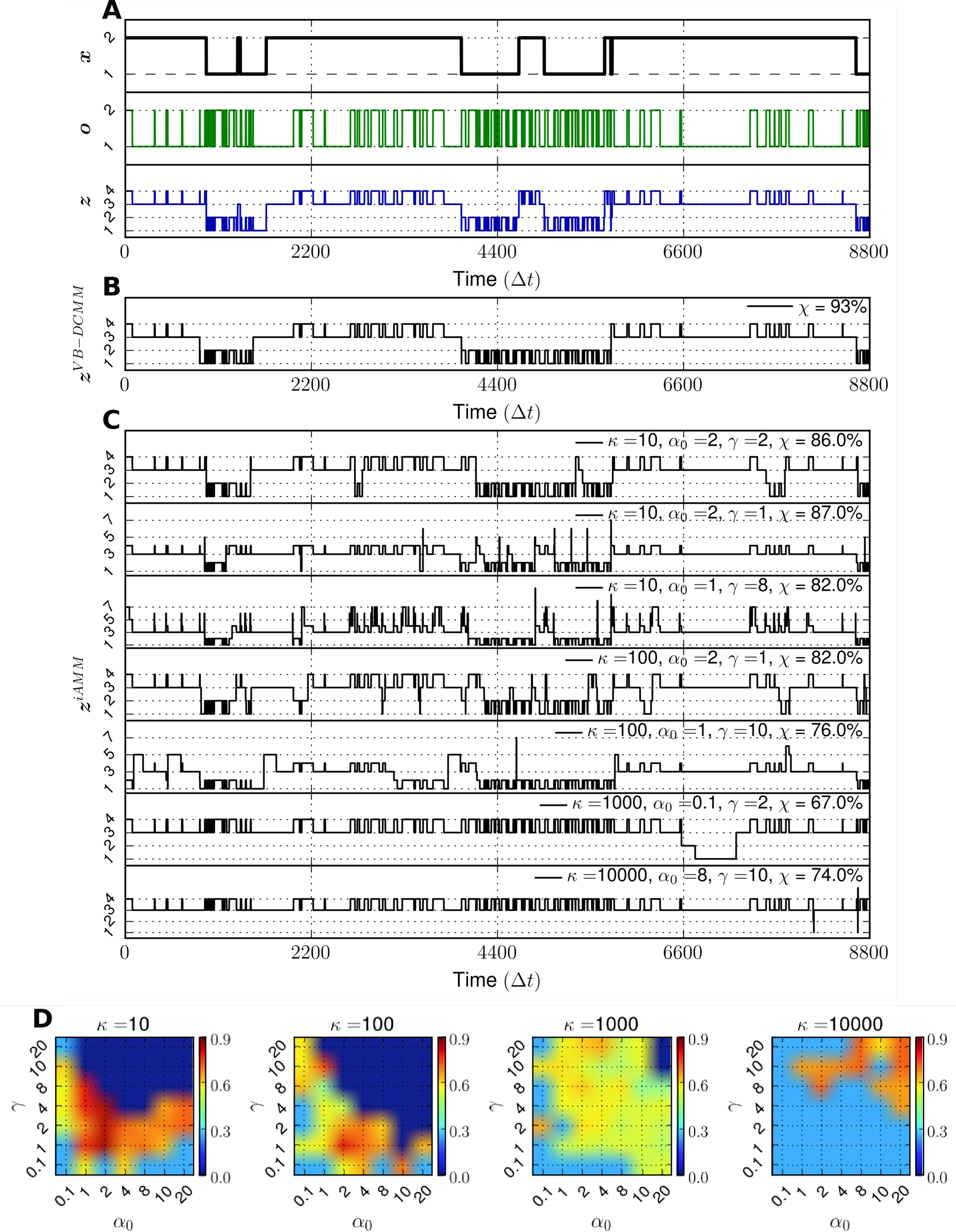

Supplement: S25 Fig — (TIF) [file pcbi.1005286.s026.tif]
